# Catalyst Activation and Speciation Involving DyadPalladate Precatalysts in Suzuki–Miyaura and Buchwald–Hartwig Cross-Couplings

**DOI:** 10.1021/acs.organomet.4c00486

**Published:** 2025-02-24

**Authors:** Neil W. J. Scott, Paula Chirila, Christopher S. Horbaczewskyj, Eric D. Slack, Adrian C. Whitwood, Ian J. S. Fairlamb

**Affiliations:** † Department of Chemistry, University of York, Heslington, York, North Yorkshire YO10 5DD, United Kingdom; ‡ Johnson Matthey PLC, 28 Cambridge Science Park, Milton Road, Cambridge CB4 0FP, United Kingdom; § Johnson Matthey, 2001 Nolte Drive, West Deptford, New Jersey 08066, United States

## Abstract

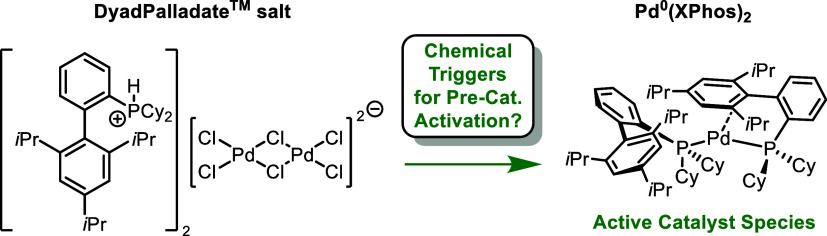

Understanding mechanisms underpinning Pd precatalyst activation and formation of active species is important in maximizing catalyst activity and lifetime. DyadPalladate precatalysts, represented by the general formula [R_3_PH^+^]_2_[Pd_2_Cl_6_]^2–^ (R_3_P = tertiary alkylphosphine/arylphosphines), have recently emerged as sustainable, active Pd precatalysts for cross-couplings (e.g., Suzuki–Miyaura {SMCC} and Buchwald–Hartwig aryl amination {BHA}). This study investigates the activation of the [HXPhos]_2_[Pd_2_Cl_6_] **1**, as a model precatalyst from the DyadPalladate class, against BHA and SMCC reactions. It was found that BHA and SMCC reactions reached the same active Pd^0^ catalyst, [Pd^0^(XPhos)_2_]. This species is generated efficiently through a reductive activation step involving a dual base/nucleophile chemical trigger. However, the mechanistic path of each is somewhat different based on the selected nucleophile. The active Pd complex participates in oxidative addition with aryl halides, the first committed step in many cross-coupling reactions. The activation pathway and catalytic efficiency of [HXPhos]_2_[Pd_2_Cl_6_] **1** were compared with those of known Pd^II^ precatalysts, possessing the XPhos ligand, through both stoichiometric and catalytic studies. Investigating the activation triggers and characterizing the active Pd^0^ catalyst, under catalytically relevant conditions, provide valuable insight into future catalyst design, targeting optimal efficiency in specific reactions, *i.e*., knowing that the precatalyst has been fully activated.

## Introduction

**Scheme 1:**
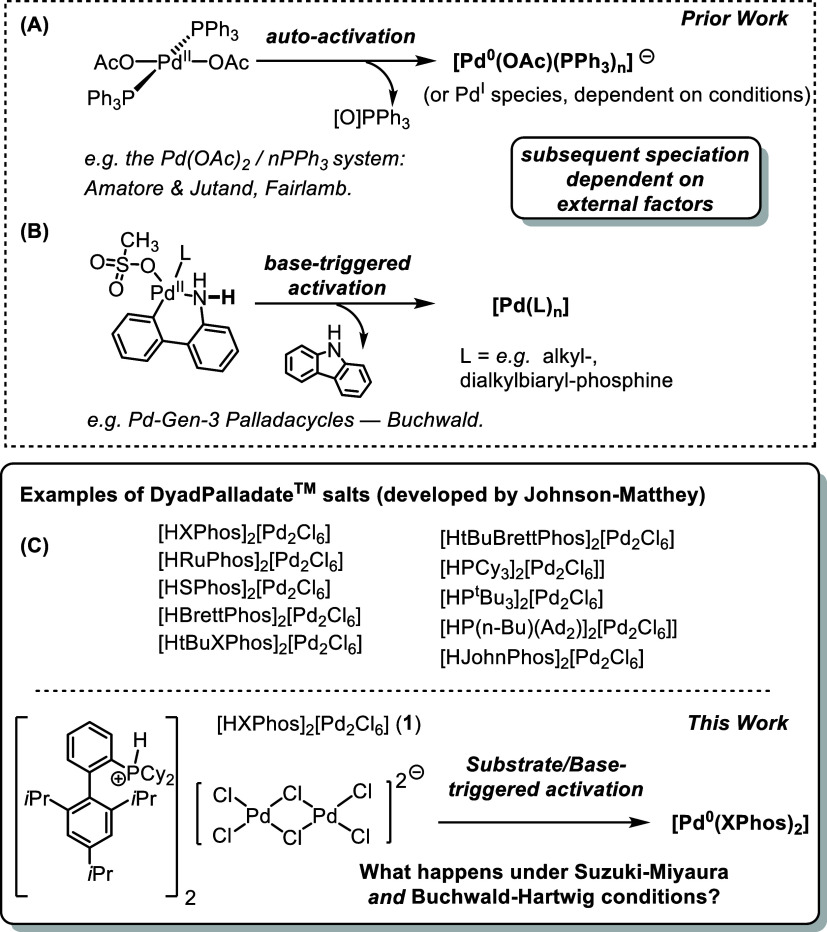
Top: Different Pd^II^ Precatalysts’ and Their Modes of Activation (A, B); Bottom: Examples of DyadPalladate Salt Precatalysts—The Activation of the XPhos Derivative **1** Is the Focus of This Study (C)

There is a continued need for economically viable Pd precatalysts that have greener and cleaner credentials. With many organizations targeting “net-zero” in their global operations, critical to that will be the redesign, refinement, and optimization of improved Pd precatalysts for their most used chemical transformations.^1^ With this in mind, a team from Johnson-Matthey have designed and developed a new class of Pd precatalysts, called the DyadPalladate precatalysts, which are easily handled Pd^II^ salts (Scheme 1).^2^ These precatalysts feature a dipalladate dianion bridged by two stabilizing chloride anions and four capping chloride anions, making up an approximately planar complex. The countercation is a protonated alkyl or aryl phosphine giving a general chemical formula [R_3_PH^+^]_2_[Pd_2_Cl_6_]^2–^. Due to the necessary Bronsted basicity of the phosphine to form the quaternary phosphonium species, a wide range of electron-rich alkyl- and dialkylbiaryl phosphines can form [R_3_PH^+^]_2_[Pd_2_Cl_6_]^2–^. In essence, the [R_3_PH^+^]_2_[Pd_2_Cl_6_]^2–^ precatalysts possess stable Pd^II^ centers and a masked phosphine, in a protonated form. This can be viewed as a natural evolution to the use of protonated phosphines like *t*-Bu_3_P.HBF_4_, developed by Fu and co-workers,^3^ which is deployed with other Pd precatalyst species. Due to a significantly larger Pd–P interatomic distance compared to the majority of Pd–L-containing precatalysts, significantly bulkier ligands can be incorporated. Comparable Pd precatalysts featuring such ligands include Buchwald palladacycles;^4^ Nolan/Hazari/Colacot π-allyl Pd catalysts,^5−8^ Pd^II^ acetates,^9,10^ and Pd^II^ chlorides.

Considerable work has gone into understanding the activation of Pd^II^ precatalysts. Common chemical triggers are the exogenous ligand (i.e., phosphine converted to phosphine oxide,^11−14^ vide infra or *N*-heterocyclic carbene converted to imidazolium salt^15^), water,^16−20^ amine^21^ (base), and/or heating. For simple nitrogen- or phosphorus-containing ligands, the activation can lead to the generation of Pd_n_ clusters and Pd nanoparticles, which are known to be competent catalyst species in a plethora of reactions.^22−24^ Use of larger phosphine ligands leads to the generation of well-defined mononuclear Pd^0^L*_n_* species.

The use of chloride salts of Pd^II^X_2_ is generally fraught with complex solubility issues and the formation of dimeric species. Use of palladium acetate with any exogenous ligand carries some risk in understanding the nature of the precatalyst activation. The chemical form of palladium acetate and the presence of contaminant anions such as nitrite may influence downstream chemistry.^25−28^ In addition, the exchange of acetate for hydroxide and alkoxides from water and alcoholic solvents can particularly convolute the active catalytic species.^29,30^ Furthermore, a hidden mechanistic complexity exists in the activation of Pd_3_(OAc)_6_ with exogenous ligands, which represents the most common practice in use today.^31−35^

Despite the tremendous success of the Buchwald palladacycles precatalysts, some drawbacks are associated with this distinct molecular scaffold. Suspected genotoxic reagents/byproducts are involved in the activation of Generation 2 and 3 palladacycle precatalysts, as well as issues with catalyst inhibition under certain conditions.^5,36^ Some of the bulkiest ligands are incompatible with precatalyst Generations 3 and 4. Furthermore, these Pd precatalysts require costly, multistep syntheses, which prevents application in cost-sensitive industrial processes.

For π-allyl Pd precatalysts, catalyst inhibition and harmful byproducts are reduced, but a costly multistep synthesis is still required to prepare them. In addition, certain π-allyl Pd precatalysts comproportionate to a less active Pd^I^-dinuclear form.^6−8^

Within the first disclosure^2^ of the [R_3_PH^+^]_2_[Pd_2_Cl_6_]^2–^ precatalysts, an efficient single-step synthesis with a low E-factor was described. The precatalysts are compatible with an eclectic array of bulky phosphine ligands (Scheme 1).

The majority of Pd^II^ precatalysts deployed for cross-coupling generally require a mandatory activation step, i.e., formal reduction from Pd^II^ to Pd^0^, at which point such species can enter into a catalytic cycle through the typical sequence of steps–oxidative addition involving the organohalide coupling partner, transmetalation with the nucleophilic component, liberating the product, and regenerating the activated Pd^0^ catalyst species following reductive elimination.^37^

Three potential mechanisms by which initial reductive activation at Pd^II^ can occur are outlined in Scheme 1. With autoactivation (A, Scheme 1), some precatalytic Pd^II^ complexes do not rely on external chemical triggers to activate; they are inherently unstable to reductive activation under ambient conditions. In this case, they require readily oxidizable functional groups embedded into the core structure. For example, *trans*-[Pd(OAc)_2_(PPh_3_)_2_] may autoactivate via formal *O*-transfer from acetate to phosphorus followed by reductive elimination at the Pd center. In this case, subsequent complex speciation is determined by external factors, such as available phosphine ligand.^11−14^ This process can also be sensitive to temperature and the time scale of activation, degrading into Pd black or forming nanoparticles. *cis*-[Pd(CH_2_Si(CH_3_)_3_)_2_(1,5-cyclooctadiene)] and Pd(η^3^-cinnamyl)(η^5^-C_5_H_5_) are other examples of precatalysts which can autoactivate. In these cases, however, activation is driven by reductive elimination at Pd with concomitant oxidative C–C bond formation.^38−40^ The role of capture of the Pd^0^ species by the 1,5-cyclooctadiene ligand is likely key from a thermodynamic standpoint.

An alternative method to reach the active Pd catalyst is via base-induced activation (B, Scheme 1), whereby deprotonation of acidic protons on the precatalyst initiates a reductive elimination process. In the case of Buchwald-Gen-1-3 palladacycles, deprotonation of the aminobiphenyl moiety followed by reductive elimination delivers [Pd^0^(L)*_n_*] complexes,^20^ along with a carbazole-containing heterocycle which can act as an inhibitory ligand.^5,36^ A third scenario, which is less well investigated and is exhibited in this work, involves substrate and base participation (C, Scheme 1), whereby nucleophilic substrate and bases can act in tandem to activate the catalyst.^29^ A slightly more nuanced variant of this mode is the allylic chloride precatalysts [PdCl(η^3^-allyl)(L)] complex studied by the groups led by Nolan, Hazari, and Colacot. In this case, the reaction base can act as a nucleophile or in tandem with a substrate nucleophile to trigger the catalyst activation via reductive elimination.^6,7^ Furthermore, alcoholic solvents have been shown to have a role in inducing catalyst activation in these systems.^8,41^ It is worth noting that although a Pd precatalyst has been delineated to activate in a particular way, it does not necessitate they exclusively activate via that mechanism under all working conditions. Thus, it is feasible that multiple activation mechanisms could simultaneously be at play in each reaction, potentially increasing the complexity of a given process.

Like many Pd^II^ chloride precatalysts, the DyadPalladate precatalysts are bench-stable, and thus not prone to autoactivation. That is critically important for stability, storage, and ultimate application in the intended environment. To understand how to best harness the reactivity of these precatalysts, we wished to investigate activation triggers and structural aspects of speciation stemming from activation. Herein, we describe the findings of our investigations examining the activation and reactivity of **1** under reaction conditions relevant to cross-coupling catalysis.

The goals of our study are listed below:

(1) Understand the activation pathway for [HXPhos]_2_Pd_2_Cl_6_ (**1**) employing chemical triggers.
(2) Evaluate mechanistic details concerning the action of **1** in topical SMCC and BHA reactions.
(3) Establish the active Pd^0^L*_n_* species derived from **1** under differing cross-coupling reaction conditions—is there a common species?

## Results and Discussion

### Studies Understanding the Behavior of **1** in Benchmark Suzuki–Miyaura Cross-Coupling (SMCC) Reactions

**Scheme 2:**
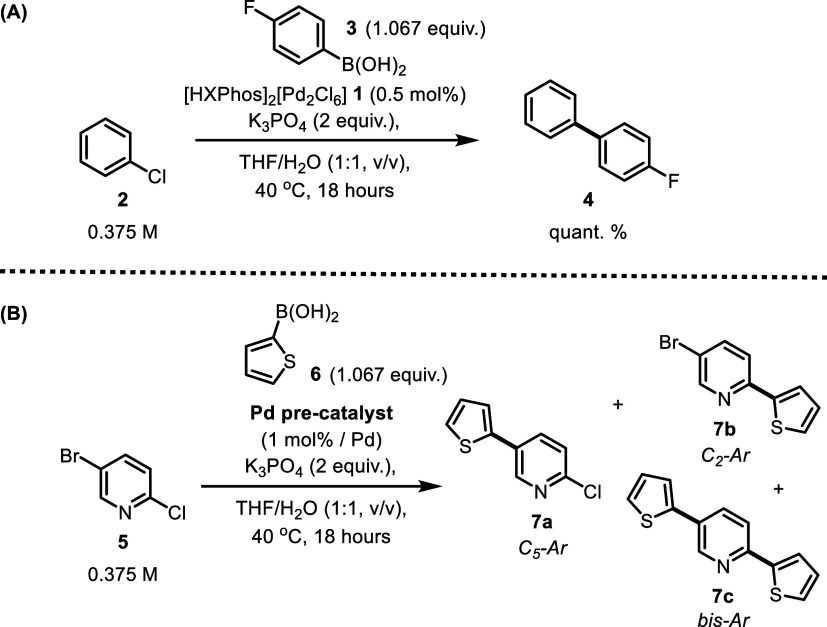
Examples of SMCC Reaction Conditions Catalyzed in the Presence of Pd Precatalysts (A) Coupling of 4-chlorobenzene with 4-fluorophenylboronic acid using precatalyst **1**. (B) Comparison between various XPhos-based Pd^II^ precatalysts for the coupling of 5-bromo-2-chloropyridine with 2-thienylboronic acid. Product yields (**4**; **7a**, **7b**, **7c**) were determined via ^1^H NMR spectroscopic analysis against a 1,3,5-trimethoxybenzene internal standard.

Being one of the top three most utilized reactions in the pharmaceutical sector, the SMCC reaction is undoubtedly the most widely used cross-coupling reaction.^42,43^ Thus, the SMCC represents an ideal reaction model to use for understanding the activation of the [HXPhos]_2_[Pd_2_Cl_6_] precatalyst **1** (Scheme 2). The XPhos variant was selected as a model ligand system due to the parent dialkylbiaryl ligand’s established success; its ubiquity and versatility as an electron-rich, sterically demanding ligand.^44−47^ These general conditions include a reaction medium consisting of tetrahydrofuran (THF)/H_2_O (1:1 v/v), with a K_3_PO_4_ base, which forms a biphasic mixture.^48^

**Figure 1:**
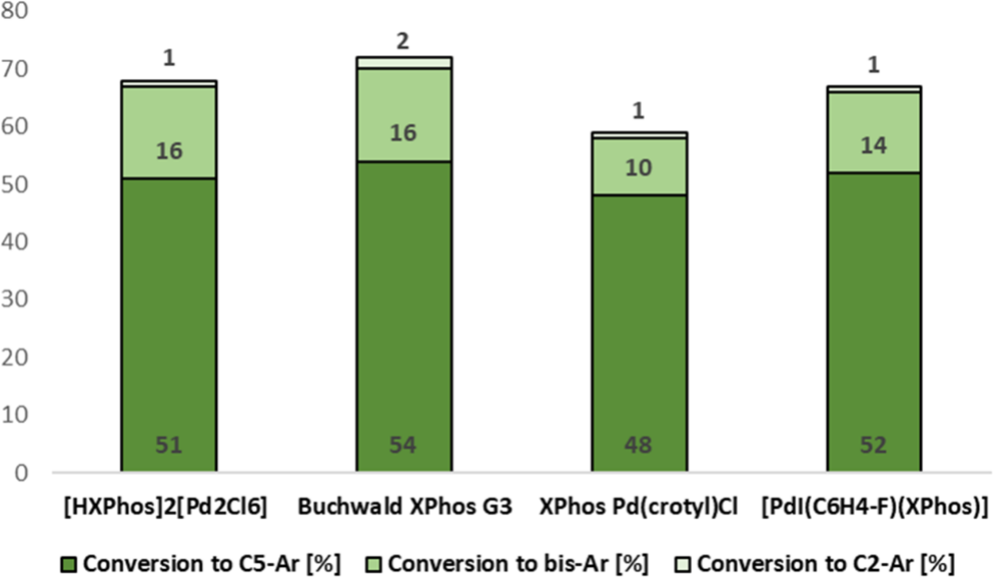
Graph showing the Pd precatalyst performance results of the chemoselective SMCC reaction as detailed in B, Scheme 2.

Two catalytic reactions were tested to assess the effectiveness of the proposed reaction conditions for SMCC reactions utilizing precatalyst (**1**) (Scheme 2A,B). The proposed reaction conditions facilitate the effective coupling of chlorobenzene **2** with 4-fluorophenylboronic acid **3** (A, Scheme 2), resulting in the formation of the desired biaryl product **4** in high yield. The same general conditions were successfully employed to couple a more challenging and synthetically versatile substrate, 5-bromo-2-chloropyridine **5**, with 2-thienylboronic acid **6**, thus creating a chemoselectivity question (B, Scheme 2). In this case, the activity of precatalyst **1** was evaluated against three other relevant XPhos-containing precatalysts: Buchwald XPhos G3, XPhos(crotyl)Cl, and the oxidative addition complex [PdI(C_6_H_4_-*p*-F)(XPhos)] **11** (Figure 1). As expected from the electronic analysis of the substrate 5-bromo-2-chloropyridine,^49^ the C_5_–Br bond activation was favored over C_2_–Cl bond activation, forming the C_5_-arylated product **7a** alongside the diarylated product **7c**.^50,51^ The monoarylated C_2_–Cl activation product **7b** was only formed in negligible amounts in each case. This trend remained consistent across all Pd^II^ precatalysts employed. Precatalyst **1** demonstrated similar activity to the other Pd^II^ precatalysts and the oxidative addition complex [PdI(C_6_H_4_-*p*-F)(XPhos)] **11**, further demonstrating the effectiveness of the DyadPalladate precatalysts as a valuable class of precatalysts. These examples, along with examples from the literature^2^ show readily that **1** can activate and couple (hetero)aromatic C–X bonds with arylboronic acids.

With operational SMCC reaction conditions in hand, the reaction conditions were altered to determine combinations of components that could initiate catalyst activation. Direct stoichiometric reactions between **1** and relevant individual components found in the SMCC reaction described above were tested.

**Figure 2:**
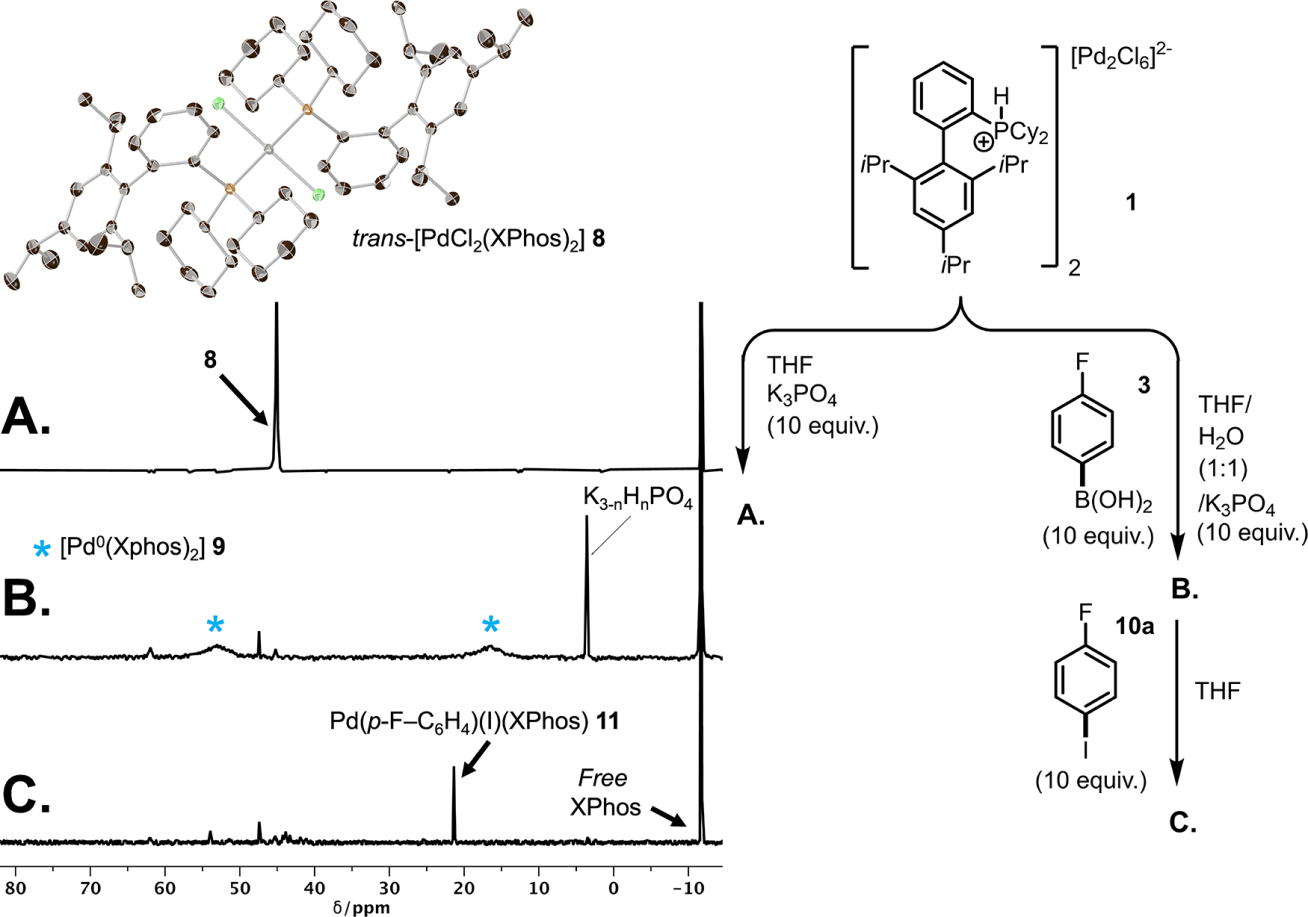
^31^P NMR stack (242 MHz, THF): experiments examining the reactivity of precatalyst 1 under three sets of conditions relevant to the Suzuki–Miyaura cross-coupling reaction: (A) Reaction with 10 equiv of K_3_PO_4_ in THF, sampled after 20 min. (B) Reaction with 10 equiv of *para*-fluorophenylboronic acid **3** and K_3_PO_4_ in THF/H_2_O sampled after 20 min sample taken from the upper (THF) layer. (C) Reaction after addition of 4-fluoro-iodobenzene **10a**, sampled after 10 min.

As the phosphonium dipalladate salts such as **1** are acidic residues, and cross-coupling reactions are carried out under basic or alkaline conditions, we first determined the reactivity with exogenous K_3_PO_4_ (5.0 equiv/Pd), in dry THF. This resulted in the formation of *trans*-[PdCl_2_(XPhos)_2_] complex **8** alongside liberated XPhos in the solution phase, as determined by ^31^P NMR spectroscopic analysis and single-crystal XRD analysis (Figure 2A). The reaction requires liberation of Y_2_PdX_4_ or PdX_2_ (where Y = H or K; X = Cl/KPO_4_/PO_4_ with the appropriate balance of anionic charge). The same reaction in THF/H_2_O (1:1), forms the same gross products; however, only a small quantity of **8** was observed by ^31^P NMR spectroscopic analysis due to the very low solubility of this particularly Pd^II^ complex in THF/H_2_O (1:1) (see the Supporting Information for further details). In both the above cases, no subsequent reactivity with 4-fluoroiodobenzene **10a** was observed (e.g., forming the oxidative addition of Pd^II^ complex **11**), providing further evidence that no reactive reduced species were present in the solution under these conditions. The activation of precatalyst **1** was also attempted using 4-fluorophenylboronic acid **3** (5.0 equiv/Pd) and K_3_PO_4_ (5.0 equiv/Pd) in dry THF, resulting again in the formation of *trans*-[PdCl_2_(XPhos)_2_] **8**, alongside liberated XPhos (see Figure S5).

**Figure 3:**
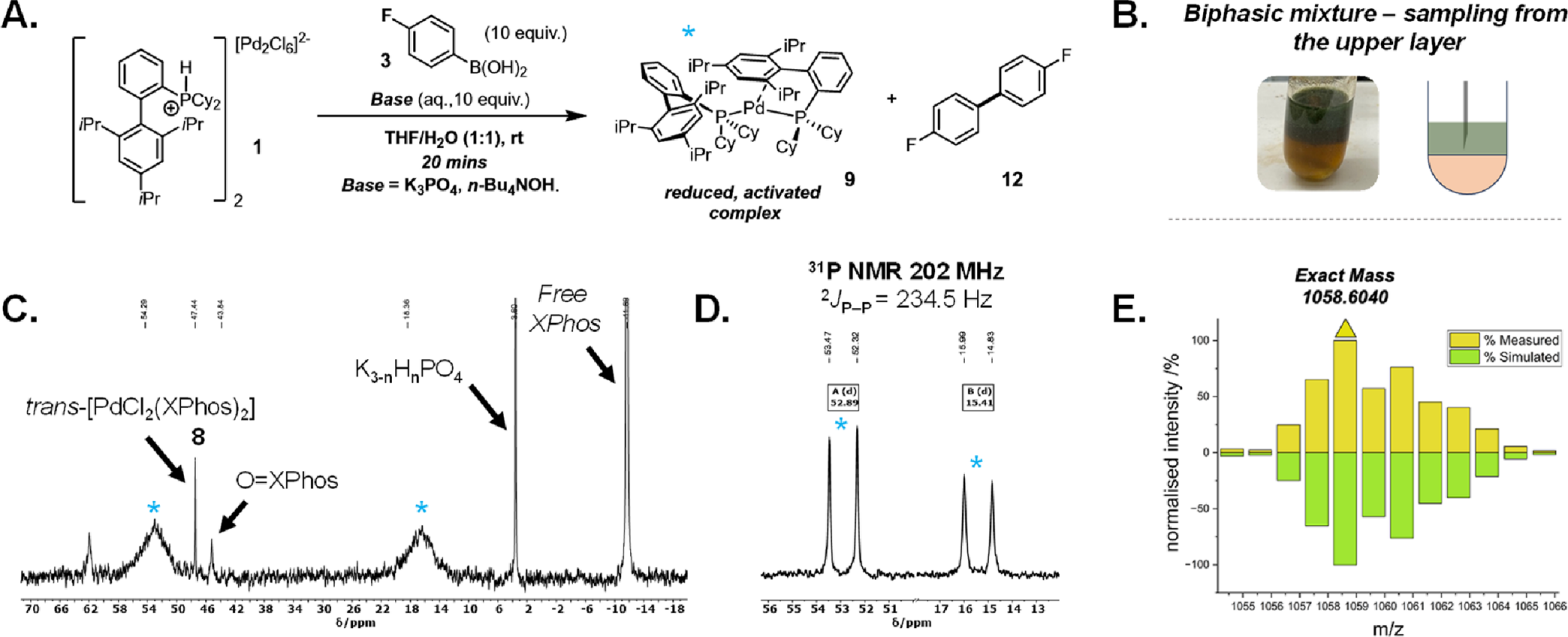
Formation of [Pd^0^(XPhos)_2_] (3) from precatalyst **1**. (A) Reaction scheme describing the range of conditions that have been found to form complex **9**, alongside 4,4′-difluorobiphenyl **12**. (B) Schematic of how sampling of the biphase was carried out, from which the reaction was sampled. (C) ^31^P NMR spectrum (242 MHz) of the upper phase (THF) of the reaction mixture, sampled after 20 min. (D) ^31^P NMR spectrum (202 MHz, 243 K) of an authentic sample of **9** generated by reaction of *cis*-[Pd(CH_2_Si(CH_3_)_3_)_2_(1,5-cyclooctadiene)] with XPhos at room temperature, showing peak resolution and the coupling constant; (E) LIFDI-MS data showing simulated and measured isotopic distribution patterns for complex **9** generated under these conditions.

Treating precatalyst **1** with 4-fluorophenylboronic acid **3** (5.0 equiv/Pd) and K_3_PO_4_ (5.0 equiv/Pd) in THF/H_2_O (1:1) led to a biphasic system. Sampling from the organic phase (B, Figure 2; according to the procedure in part B, Figure 3) revealed the formation of a novel Pd species. This species was characterized by ^31^P NMR as two broad resonances at **δ**_P_ 16.7 and 55.2 ppm corresponding to the reduced zerovalent complex [Pd^0^(XPhos)_2_] **9** (B, Figure 2; C, Figure 3). Low-temperature NMR in THF allowed for peak resolution, revealing a ^31^P–^31^P coupling constant of 235 Hz (D, Figure 3). This coupling constant is consistent with a mutual *trans*-relationship between two coordinated phosphines at Pd^0^.

The homocoupled biaryl product of the boronic acid (4,4′-difluorobiphenyl, **12**) was detected as a by-product, formed by the formal reduction process. The presence of this compound was further confirmed via a spiking experiment (see the Supporting Information for further details).

The generation of [Pd^0^(XPhos)_2_] **9** has been previously reported by Jutand, Grimaud, and co-workers, in this case formed by reacting “Pd(OAc)_2_” and XPhos (3.0 equiv) at room temperature, as characterized by NMR.^52^ Furthermore, Fink and co-workers have reported the CyJohnPhos analogue which can be generated from the autoactivation of [Pd(CH_3_)_2_(TMEDA)].^53^ To confirm the presence of the active Pd^0^-complex **9**, a sample of the same solution was analyzed by LIFDI-MS (a soft ionization method for characterizing organometallic ions).^54,55^ The mass spectrum revealed a peak at 1058.60 *m*/*z*, corresponding to the *m*/*z* of [Pd(XPhos)_2_]^+^ with the correct isotopic distribution (E, Figure 3). Further evidence to support these claims was provided by reaction with 4-fluoroiodobenzene **10a** at room temperature. Upon addition of the aryl halide the two broad phosphorus signals (**δ**_P_ 16.7 and 55.2) consistent with **9** (possessing a distorted “P,*C*–Pd-P” arrangement, as shown in structure **9**, Figure 3) disappeared and were replaced by a peak at **δ**_P_ 21.4 ppm, indicative of an oxidative addition complex [PdI(C_6_H_4_-*p*-F)(XPhos)] **11** (C, Figure 2).^52^ This is in agreement with the ^31^P NMR spectrum of an authentic sample of **11** synthesized from [Pd(CH_2_Si(CH_3_)_3_)_2_(1,5-cyclooctadiene)] (see the Supporting Information for further details).^56^ LIFDI-MS further supported this structure with [PdI(XPhos)]^+^ and [Pd(C_6_H_4_-*p*-F)(XPhos)]^+^: [M-(C_6_H_4_-*p*-F)]^+^ and [M – I]^+^ fragments, respectively, with the correct isotopic distribution pattern (see the Supporting Information for further details).

Among a variety of bases studied for this activation process, K_3_PO_4_ and Na_2_CO_3_ were able to best generate [Pd^0^(XPhos)_2_] **9** (see the Supporting Information for further details). Employing a stronger base such as *n-*Bu_4_NOH also resulted in the formation of **9**; however, under these conditions, a low concentration of the complex was observed due to phase combination and subsequent complex precipitation. Weak bases, such as KI, could not efficiently generate the Pd^0^ active species. The formation of an active catalyst using a variety of inorganic bases was further tested by the addition of 4-fluorobromobenzene **10b** to investigate the formation of the oxidative addition complex. It was possible to generate the oxidative addition complex [PdBr(C_6_H_4_-*p*-F)(XPhos)] **12**, using Na_2_CO_3_ and K_3_CO_3_ as bases. Water facilitates the solubility of base and promotes the reductive activation mechanism to deliver active Pd^0^, an observation which has been made before with such reductive activation mechanisms.^20,21,29^ However, it should be noted that **9** could also be generated in the absence of intentionally added water, in dried solvent, at elevated temperatures (60 °C).

[Pd^0^(XPhos)_2_] **9** could also be generated by a variety of *para*-substituted phenylboronic acids under identical conditions: 4-methoxyphenyl, phenyl-, and 4-trifluoromethylphenyl boronic acid, indicating a relatively wide electronic tolerance of the precatalyst **1** activation reaction with arylboronic acids. When employing 4-trifluoromethyphenylboronic acid, the formation of **9** in the absence of purposely added H_2_O was observed by ^31^P NMR spectroscopic analysis after *ca*. 20 min. This observation suggests that electron-poor arylboronic acids might be more efficient in this reductive activation compared to electron-rich ones. Notably, this observation contrasts with expectations for reductive elimination at a biaryl Pd^II^ complex.^57^

Having demonstrated that the *trans*-[PdCl_2_(XPhos)_2_] complex **8** is directly formed from the reaction between precatalyst **1** and K_3_PO_4_ (A, Figure 2), it was important to investigate whether treating **8** with 4-fluorophenylboronic acid **3** under identical conditions would lead to the formation of the Pd^0^ complex **9**. However, **9** was not detected following the reaction at room temperature (22 °C), suggesting that the activation of precatalyst **1** under these conditions does not proceed via complex **8**, which is a potential intermediate.

### Buchwald–Hartwig Amination Reaction Conditions

**Scheme 3:**
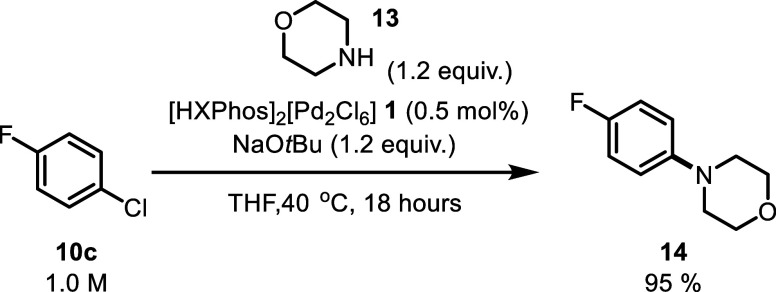
BHA Reaction under Typical Conditions, Coupling of 4-Fluorochlorobenzene **10c** with Morpholine %Yield determined by comparison against a 1,3,5-trimethoxybenzene internal standard.

The Buchwald–Hartwig aryl amination (BHA) reaction has emerged as a powerful tool for the *N*-arylation of nitrogen-containing substrates such as primary and secondary amines, ammonia, and amides.^47^ It is a highly utilized reaction due to the ubiquity of *N*-containing moieties, particularly in pharmaceuticals, agrochemicals, and other bioactive molecules. Thus, this reaction was selected as a model reaction for the investigation of the activation mechanism of precatalyst **1**. In line with the previous (SMCC) reaction model, a set of typical BHA reaction conditions were validated using precatalyst **1** to efficiently couple 4-fluorochlorobenzene **10c** with morpholine **13** in THF, forming C–N-coupled product **14** in a 95% yield (Scheme 3).

**Figure 4:**
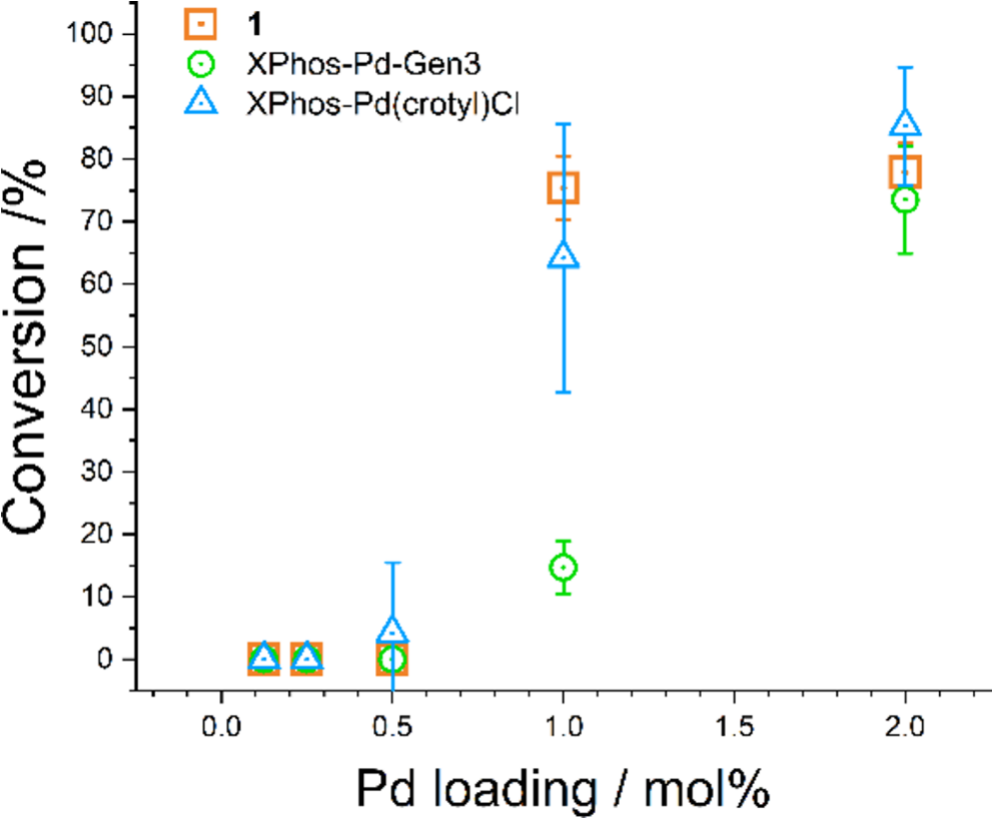
Conversion (%) of 6-chloroquinoline **15** to the C–N coupled product **16** using precatalyst **1** (Scheme 4), XPhos-Pd-Gen3 and XPhos-Pd(crotyl)Cl as precatalyst with different Pd loadings (0.125, 0.25, 0.5, 1.0, 2.0 mol %). Conversions were determined by using LC-MS analysis.

**Scheme 4:**
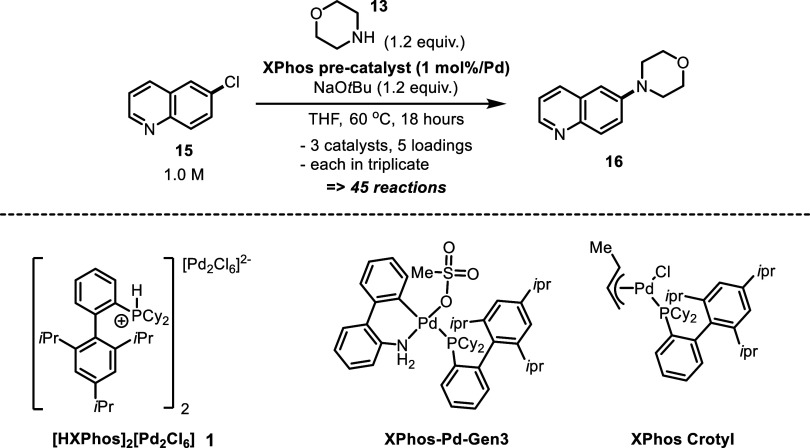
BHA Reaction for HTE Catalyst Screening Using a Chemspeed ISYNTH Robotic System

Related conditions were applied to the coupling of morpholine with the heteroaromatic aryl halide, 6-chloroquinoline **15** forming the C–N-coupled product **16**; however, two other XPhos-containing precatalysts were also tested for this transformation using a Chemspeed ISYNTH robotic system. The activity of the Pd^II^ precatalysts was compared across 45 reactions run in parallel (Scheme 4 and Figure 4). Three control reactions were also carried out with no added Pd^II^ precatalyst, showing no conversion to the BHA product. The screening assessed the following precatalysts: **1**, the Buchwald palladacycle [Pd^II^(OMs)(2-aminobiphenyl)(XPhos)]/“XPhos-Pd-Gen3″, and the π-allyl complex [Pd^II^Cl(η^3^-crotyl)(XPhos)]/“XPhos-Pd(crotyl)Cl”. Each precatalyst was applied to the reaction using five different precatalyst loadings (0.125, 0.25, 0.5, 1.0, 2.0 mol %).

The data reported in Figure 4 show the relative catalytic activity as well as reproducibility data for the three commercial precatalysts, each containing XPhos as a tertiary phosphine ligand. The results show that under the applied reaction conditions at 2.0 mol %/Pd loading, all three precatalysts display high activity, with XPhos-Pd-Gen3 displaying reduced activity at 1.0 mol % Pd loading. No precatalyst showed notable conversion at loadings <1 mol % Pd under the conditions examined. Minimal variance was noted for **1** under these reaction conditions.

There are key differences between the BHA reaction conditions (Schemes 3 and 4) and SMCC reaction conditions (Scheme 2). The BHA reaction generally requires a significantly stronger base, e.g., sodium *tert*-butoxide (NaO*t*Bu).^56,58^ Amine basicity and potential for coordination to the Pd center, thereby influencing (pre)catalytic speciation, is also key.^16,18^

With a set of established reaction conditions in hand, we envisaged utilizing the same approach to examine precatalyst activation of **1** under the BHA reaction conditions. Thus, systematic stoichiometric reactions of precatalyst **1** were carried out with BHA-relevant reagents, derived from operational coupling conditions.

**Figure 5:**
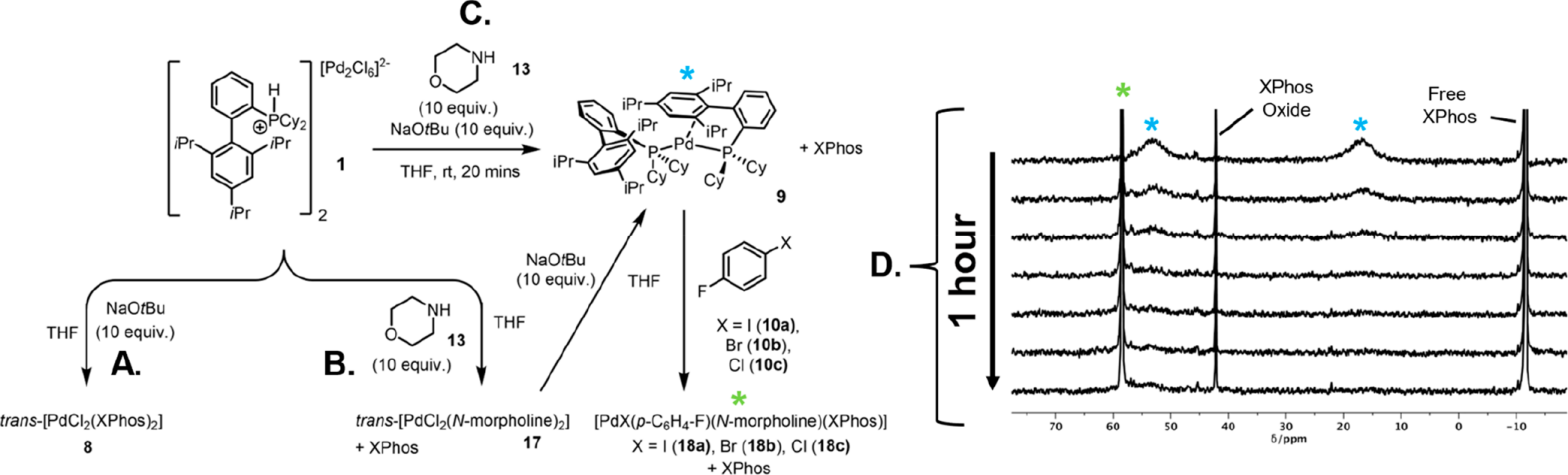
Summary of the reaction between XPhos DyadPalladate precatalyst **1** with various BHA reaction components. Reaction of **1** with (A) NaO*t*Bu (5.0 equiv/Pd) in THF; (B) morpholine (5.0 equiv/Pd) in THF; (C) NaO*t*Bu (5.0 equiv/Pd) and morpholine (5.0 equiv/Pd) in THF; and (D) addition of aryl halides.

First, precatalyst **1** was reacted directly with NaO*t*Bu in THF for 20 min. ^31^P NMR spectroscopy allowed the detection of free XPhos, alongside small amounts of *trans*-[PdCl_2_(XPhos)_2_] **8** and XPhos oxide (A, Figure 5). Second, precatalyst **1** was reacted with morpholine (5.0 equiv/Pd) resulting in the observation of free XPhos and minor unknown species at 57 ppm by ^31^P NMR (B, Figure 5). In this instance, insoluble yellow powder *trans*-[PdCl_2_(*N*-morpholine)_2_] **17** was identified as a major species by IR spectroscopic analysis. These observations indicate that upon deprotonation of the phosphonium cation, XPhos is liberated and morpholine preferentially ligates with Pd to yield **17** as the kinetic product. No activated Pd species was observed from either of these reactions (A or B, Figure 5), as evidenced by the absence of [Pd^0^(XPhos)_2_] **9**, and the expected oxidative addition product was observed upon the addition of 4-fluoroiodobenzene **10a** (or 4-fluorochlorobenzene **10c**) to the reactions of precatalyst **1** with either NaO*t*Bu or morpholine.

When precatalyst **1** was reacted with both NaO*t*Bu and morpholine (both 10 equiv per **1**; 5.0 equiv/Pd), two broad phosphorus signals (**δ**_P_ 16.7, 55.2 ppm) consistent with [Pd^0^(XPhos)_2_] **9** were again detected by ^31^P NMR, alongside free XPhos (C, Figure 5). The formation of **3** was further supported by the LIFDI-MS data. These results indicate that, in a similar fashion to the SMCC activation, a concert of base and nucleophilic coupling partners are needed for catalyst activation to **9**. It is of note that *trans*-[PdCl_2_(*N*-morpholine)_2_] **17** also generates **9**, as observed by ^31^P NMR, when reacted with NaO*t*Bu in the presence of XPhos (2.0 equiv/Pd), identifying **17** as a possible intermediate en route to Pd reduction.

*Trans*-[PdCl_2_(XPhos)_2_] **8** was also reacted with NaO*t*Bu and morpholine under the same reaction conditions utilized for the activation of **1**. However, an active Pd^0^ species was not observed under these conditions. Notably, however, [Pd^0^(XPhos)_2_] **9** could be generated from **2** at an elevated temperature of 60 °C (see the Supporting Information for further information), demonstrating a slower formation of Pd^0^ species from **8** when compared with precatalyst **1**.

Secondary alkyl amines, such as morpholine **13**, have previously been found to be involved in the reductive activation step of Pd^II^ precatalysts by deprotonation of the N–H followed by β-hydride elimination.^59,60^ As it has been shown here that morpholine in tandem with NaO*t*Bu is required for reductive activation of precatalyst **1** to give [Pd^0^(XPhos)_2_] **9**. We ran control experiments to determine which aspects of the morpholine structure were responsible for reduction to Pd(0). Neither the tertiary cyclic amine analogue of morpholine, *N*-methylmorpholine (lacking an N–H-bond), nor aniline (lacking a β–hydrogen) led to the formation of **9** when reacted with **1** in THF, and the presence of NaO*t*Bu at either 22 or 60 °C. Furthermore, the oxidative addition product (e.g., [PdI(C_6_H_4_-*p*-F)(XPhos)]) **11** was not formed upon addition of 4-fluoroiodobenzene **10a** to the solution (confirmed by ^31^P NMR). This observation might suggest that the presence of both N–H and β-hydrogens is required for efficient activation by the amine nucleophile (note: no oxidized byproduct was detected by either NMR or GC-MS). In line with this observation, BHA catalytic reactivity of 4-fluorochlorobenzene **10c** with aniline resulted in 0% conversion to the BHA cross-coupled product under the conditions reported. These results suggest that the inability of anilines to undergo BHA cross-coupling is in part due to the sluggish formation of the active Pd catalyst under the reaction conditions.

When 4-fluoroiodobenzene **10a** was added to [Pd^0^(XPhos)_2_] **9**, generated from the reaction of **1** with NaO*t*Bu and morpholine (D, Figure 5), a new signal was observed at **δ**_P_ 61 ppm (^31^P NMR). This is a significant downfield shift for the expected oxidative addition complex at **δ**_P_ 21 ppm of [PdI(C_6_H_4_-*p*-F)(XPhos)] **11**, indicative of a different species. The identity of this species has been assigned as the morpholine-ligated oxidative addition 16-electron Pd^II^ complex [PdI(C_6_H_4_-*p*-F)(*N*-morpholine)(XPhos)] **18a**. A control experiment to confirm the identity of **18a** was conducted, where a reference sample of **11** was treated with morpholine, resulting in the formation of the same species according to NMR spectroscopic analysis (see the Supporting Information for further details). A similar reaction involving 4-fluorobromobenzene **10b** led to formation of [PdBr(C_6_H_4_-*p*-F)(*N*-morpholine)(XPhos)]**18b**. As aryl chloride bonds are more challenging to activate, the time scale of oxidative addition of [Pd^0^(XPhos)_2_] **9** to the C–Cl bond of 4-fluorochlorobenzene **10c** was slow enough to track by ^31^P NMR spectroscopic analysis at 25 °C, showing loss of the peaks representative of [Pd^0^(XPhos)_2_] **9** (**δ**_P_ 16.7, 55.2 ppm) over time, and the appearance of a new phosphorus signal at **δ**_P_ 61 ppm, representative of the [PdCl(C_6_H_4_-*p*-F)(*N*-morpholine)(XPhos)] **18c**. The oxidative addition reaction is supported by LIFDI-MS analysis with [Pd(C_6_H_4_-*p*-F)(XPhos)]^+^ ([M – Cl – **13**]^+^) and [PdCl(XPhos)]^+^ ([M – aryl – **13**]^+^) peaks being detected (*m*/*z* at 617.2288 and 677.2901, respectively). Although the detection of these ions support the occurrence of an oxidative addition reaction, no morpholine adducts were detected by MS, highlighting the loosely bound nature of this amine ligand.

## Conclusions

In conclusion, the behavior of a model DyadPalladate precatalyst containing the ubiquitous XPhos ligand has been investigated (precatalyst **1**), with a focus on precatalyst activation under established model Suzuki–Miyaura (SMCC) and Buchwald–Hartwig (BHA) reaction conditions. Under both SMCC and BHA-type reaction conditions, the base and nucleophile act in unison to activate precatalyst **1** to deliver [Pd^0^(XPhos)_2_] **9**, which can then react in oxidative addition reactions with aryl halides. In the case of the BHA model, the oxidative addition complex was shown to be ligated to the nucleophilic substrate morpholine, forming [PdX(C_6_H_4_-*p*-F)(*N*-morpholine)(XPhos)] **18**. While no change was observed in the ability to form [Pd^0^(XPhos)_2_] **9** as a function of the assessed substituted arylboronic acid electronics under SMCC conditions, changing the nucleophile to aniline or *N-*methylmorpholine did not form [Pd^0^(XPhos)_2_] **9** under BHA conditions. These results indicate that the substrate is a key factor not only in the cross-coupling step but also in the precatalyst activation step.

It is important to acknowledge that [Pd^II^Cl_2_(XPhos)_2_] **8** can form from **1** when treated with a base (Figure 2). This precatalyst is reported to require more forcing reaction conditions for successful SMCC reactions, than the examples reported herein.^61^

Understanding the activation mechanism of Pd precatalysts is crucial in designing more efficient and sustainable chemical processes.^62^ Having access to this knowledge enhances our ability to better predict the catalyst behavior and develop novel Pd precatalysts with tailored properties for applied processes. Ultimately, the true efficacy of a ligand or Pd precatalyst/ligand system depends on a controlled triggered activation to bring about the formation of the active catalyst species, as a single, clean entity. With the ever-increasing use of high-throughput experimentation in organometallic chemistry and catalysis,^63^ this point requires careful consideration.

Lastly, we believe that the DyadPalladate complexes can complement other means of generating Pd(0)L*_n_* species in situ, and their reactions with aryl halides to generate oxidative addition intermediates.^64^ Indeed, in order to meet net-zero targets, the pragmatic and careful design of simpler and sustainable Pd precatalysts is critical for future utilization of cross-coupling chemistries.

## Supporting Information Available

Catalytic reaction experiments, stoichiometric experiments involving **1**, XRD, and other details for synthesis and characterization of compounds. (PDF)

## Notes

DyadPalladate precatalysts, including **1**, and crotyl Pd precatalysts, are both intellectual property of Johnson-Matthey.

## Abbreviations

BHA: Buchwald–Hartwig amination
CyJohnPhos: 2-(dicyclohexylphosphino)biphenyl
OMs: mesylate (OSO_2_CH_3_)
SMCC: Suzuki–Miyaura cross-coupling reaction
THF: tetrahydrofuran
XPhos: 2-dicyclohexylphosphino-2′,4′,6′-triisopropylbiphenyl
