# Supplementary material for: Catalyst Activation and Speciation Involving DyadPalladate Precatalysts in Suzuki–Miyaura and Buchwald–Hartwig Cross-Couplings
Source: Organometallics. 2025 Feb 24;44(5):654–64. doi: 10.1021/acs.organomet.4c00486 (PMC11898177; doi:10.1021/acs.organomet.4c00486)
Supplement: Supplementary file 1 — om4c00486_si_001.pdf [file om4c00486_si_001.pdf]

Supporting Information

Catalyst Activation and Speciation Involving DyadPalladate pre-catalysts in Suzuki-Miyaura and Buchwald-Hartwig cross-couplings

Neil W. J. Scott,[a] Paula Chirila,[b] Christopher S. Horbaczewskyj,[a] Eric D. Slack,[c] Adrian C. Whitwood,[a] and Ian J. S. Fairlamb[a] *

[a] Department of Chemistry, University of York, Heslington, York, North Yorkshire, YO10 5DD, United Kingdom.

[b] Johnson Matthey PLC, 28 Cambridge Science Park, Milton Road, Cambridge, CB4 0FP, United Kingdom.

[c] Johnson Matthey 2001 Nolte Drive, West Deptford, New Jersey, 08066 United States.

Email:* ian.fairlamb@york.ac.uk

Table of Contents

1. General Information 4

1.1. Compound Preparatory Techniques and Methods for In-laboratory Analysis 4

1.2. Instrument Details and Methods for Compound Characterization 5

2. Experimental Details 7

2.1. General Procedures 7
2.1.1. Air sensitive NMR sampling *via* Schlenk Conditions 7
2.1.2. Suzuki-Miyaura Cross-Coupling Test Reaction Conditions 8
2.1.3. 5-Bromo-2-chloropyirdine/2-thienyl boronic acid – chemoselective Suzuki–Miyaura coupling 11

2.2. Stoichiometric reactivity – Suzuki–Miyaura-type conditions 16
2.2.1. $[HXPhos]_2[Pd_2Cl_6]$ reaction with K_3PO_4 in THF, with and without *para*-fluorophenyl boronic acid; Identification of *trans*-$[PdCl_2(XPhos)_2]$ as a reaction product. 16
2.2.2. Solubility studies of *trans*-$[PdCl_2(XPhos)_2]$ (8) 18
2.2.3. $[HXPhos]_2[Pd_2Cl_6]$ 1 reaction with K_3PO_4 and *para*-fluorophenyl boronic acid in THF 18
2.2.4. $[HXPhos]_2[Pd_2Cl_6]$ 1 reaction with K_3PO_4 and *para*-fluorophenyl boronic acid in THF/H_2O 19
2.2.5. Homocoupled product spiking experiment 23
2.2.6. Identification of $K_nH_{3-n}PO_4$ in the reaction mixture. 23
2.2.7. Generation of $Pd^0(XPhos)_2$ 9 in presence of differing arylboronic acids 24
2.2.8. $[HXPhos]_2[Pd_2Cl_6]$ activation using various bases 25
2.2.9. Synthesis of $Pd(XPhos)_2$ by reaction of $[Pd(CH_2SiMe_3)_2(1,5\text{-cyclooctadiene})]$ with XPhos in THF 26
2.2.10. Variable Temperature (VT) ^{31}P NMR experiment 27
2.2.12 Quantification of oxidative addition complex 11 generated from $[HXPhos]_2[Pd_2Cl_6]$ 1 under Suzuki–Miyaura-type reaction conditions 28
2.2.13. Quantification of oxidative addition complex generated after activation of $[PdCl_2(XPhos)_2]$ under Suzuki–Miyaura-type reaction conditions 29
2.2.14. Synthesis of $[Pd^{II}(I)(p\text{-}C_6H_4F)(XPhos)]$ as an authentic standard 30
2.2.15. $[HXPhos]_2[Pd_2Cl_6]$ 1 reaction with K_3PO_4 and *para*-flurophenyl boronic acid in THF at 60 ˚C. 31

2.3. Stoichiometric reactivity – Buchwald–Hartwig-type conditions 32
2.3.1. Reaction of $[HXPhos]_2[Pd_2Cl_6]$ with NaO*t*Bu (A, Figure S5 main paper) 32
2.3.2. Reaction of $[HXPhos]_2[Pd_2Cl_6]$ 1 with morpholine (B, Figure S5, main paper) 32
2.3.3. Reaction of $[HXPhos]_2[Pd_2Cl_6]$ 1 with NaO*t*Bu and morpholine and oxidative addition reactions therefrom (C, D; Figure S5, Main Paper) 33
2.3.4. Reaction of *trans*-$[PdCl_2(N\text{-morpholine})_2]$ with NaO*t*Bu and XPhos 34
2.3.5. Reaction of authentic $[Pd^{II}(Br)(p\text{-}C_6H_4F)(XPhos)]$ with morpholine. 35
2.3.6. Reaction of $[HXPhos]_2[Pd_2Cl_6]$ 1 with NaO*t*Bu and *N*-methyl morpholine 35
2.3.1. Reaction of $[HXPhos]_2[Pd_2Cl_6]$ 1 with NaO*t*Bu and aniline 36

2.4. Buchwald–Hartwig Catalytic Activity Experiments 36
2.4.1. General procedure for Buchwald–Hartwig test reactions 36
2.4.2. General workup procedures: Buchwald–Hartwig amination reactions 37

2.4.3. Catalyst screening of Buchwald–Hartwig coupling reaction: XPhos-containing pre-catalyst *vs*. concentration 38

3. Compound Characterization Data 44

3.1. 4-Fluoro-1,1'-biphenyl (4) [11] 44

3.2. 4-(4-Fluorophenyl)morpholine (14) [12,13] 44

3.3. 4-(Quinolin-6-yl) morpholine (16) [14] 45

3.4. *trans*-bis(2-Dicyclohexylphosphino-2′,4′,6′-triisopropylbiphenyl) palladium dichloride; trans-[$PdCl_2(XPhos)_2$] (8) [15] 46

3.5. Bis(*N*-morpholine) palladium dichloride (17) [16-17] 49

4. NMR Spectra 50

5. Abbreviations 64

6. References 65

1. General Information

1.1. Compound Preparatory Techniques and Methods for in-Laboratory Analysis

Reagents were purchased from Merck (Sigma-Aldrich), Alfa Aesar, Acros Organics, ThermoFisher Scientific or Fluorochem and used as received unless otherwise stated. $[HXPhos]_2[Pd_2Cl_6]$ (Pd-192) and other DyadPalladate^{TM} pre-catalysts were provided by Johnson Matthey PLC. The pre-catalyst was synthesised using the previously reported procedure.[1, 2] 6-Chloroquinoline was purchased from Fluorochem and further purified by flash-column chromatography (EtOAc/hexane; 30:70). C, H, N elemental analysis: Average of two runs: %C = 66.04, %H = 3.59, %N = 8.49, % remainder = 21.88; theoretical values, for C_9H_6ClN: %C = 66.07, %H = 3.70, %N = 8.56, % remainder = 21.67. Aryl boronic acids were purchased from Tokyo Chemical Industries (TCI), ThermoFisher Scientific or Fluorochem and used as received. Aniline was purchased from Merck, dried over CaH_2, distilled, degassed by bubbling and stored in the dark under aluminium foil. Morpholine, purchased from Merck, was dried over CaH or dried over activated 4 Å molecular sieves before either being freeze-pump-thaw-degassed or deoxygenated by bubbling N_2 for ca. 30 minutes prior to use and distilled by vacuum transfer and stored in an ampoule. *para*-Fluoroiodobenzene was either freeze-pump-thaw-degassed or deoxygenated by bubbling N_2 for ca. 30 minutes prior to use before being stored in an ampoule over activated molecular sieves.

THF was dried by refluxing over finely-sliced sodium metal (2 × 8 hours), before being distilled, transferred to an ampoule *via* cannula and subsequently deoxygenated by bubbling with argon for *ca.* 30 min. Alternatively THF (99.5%, extra dry over Molecular sieves) was purchased from Thermo Scientific and subsequently deoxygenated by bubbling with N_2 for *ca.* 30 min. CD_2Cl_2 was dried for *ca*. 2 days over CaH_2 before being freeze-pump-thaw degassed and distilled into an ampoule and stored under inert gas (Ar or N_2). Petroleum ether (petrol/PET) refers to the fraction of petroleum that distils at 40-60 °C. Brine refers to a saturated, aqueous solution of NaCl.

All reactions requiring air-sensitive techniques were carried out either using an Ar-filled glovebox or using Schlenk techniques (high vacuum, liquid nitrogen trap on a standard in-house built dual line manifold {vacuum and N_2}), to eliminate atmospheric air or adventitious moisture from the reaction systems.[3] Room temperature upper and lower limits are stated as 13-25 °C, but typically 21 °C was recorded. Thin layer chromatography (TLC) was carried out using Merck 5554 aluminium–backed silica plates (silica gel 60 F254) and spots were generally visualized using UV light (at 254 nm). Retention factors (R_f) are reported in parentheses along with the solvent system used. Flash column chromatography was

performed using Sigma-Aldrich 60 Å silica gel (SiO_2, particle size 40–63 μm) and a solvent system as reported in the main text below.

1.2. Instrument Details and Methods for Compound Characterization

NMR spectra were obtained in the solvent indicated in the text below, using a Bruker NMR AVIIIHD 600 Widebore (600 MHz [^{1}H], 564 MHz [^{19}F], 242 MHz [^{31}P] 125 MHz [^{13}C]), Bruker AVIIIHD 500 instrument (500 MHz [^{1}H], 470 MHz [^{19}F], 203 MHz [^{31}P] 125 MHz [^{13}C]) or JEOL ECX400, JEOL ECS400 spectrometer (400 MHz [^{1}H], 101 MHz [^{13}C] and 377 MHz [^{19}F], 162 MHz [^{31}P]) or Bruker Avance III HD (400 MHz [^{1}H], 101 MHz [^{13}C] and 377 MHz [^{19}F], 162 MHz [^{31}P]). For ^{1}H NMR chemical shifts (δ) are reported in parts per million (ppm) and were referenced to the residual non-deuterated solvent of the deuterated solvent used; C*H*Cl_3 : δ ^{1}H = 7.26 and ^{13}C = 77.16 (*C*DCl_3), CD_2Cl_2 :^{1}H = 5.31 (CD*H*Cl_2) and ^{13}C = 54.0, THF-*d₈* δ ^{1}H = 3.59 (OCH_2CH_2), ^{13}C = 67.57 (O*C*H_2CH_2), ^{1}H = 1.73 (OCH_2CH_2) ^{13}C = 25.37 (OCH_2CH_2). Spectra were typically run at a temperature of 298 K (25 ^{o}C). ^{31}P NMR spectra were carried out with proton decoupling, unless otherwise stated. ^{31}P NMR spectra were typically recorded using 128 scans and a spectral window of 300 ppm (δ 250 to -50 ppm). Chemical shifts for ^{31}P resonances were calibrated by externally referencing to an 85% H_3PO_4 in H_2O (w/w). In practice, this was carried out by inserting a sealed, vacuum dried capillary tube containing 85% H_3PO_4 in H_2O (w/w) into an NMR tube containing the sample of interest, collecting a ^{31}P NMR spectrum and setting the H_3*P*O_4 resonance to 0 ppm. Alternatively, triphenyl phosphate and triphenyl phosphine oxide were used as internal standards. All ^{31}P and ^{13}C NMR spectra were obtained with ^{1}H decoupling. All NMR spectra were processed using MestReNova (MNova) software (using versions 12–14) or Bruker Topspin 3.6.5.

HRMS ESI-MS spectra were measured using a Bruker Daltronics micrOTOF MS, Agilent series 1200LC with electrospray ionisation (ESI) or on a Thermo LCQ using electrospray ionisation, with <5 ppm error recorded for all HRMS samples. LIFDI mass spectrometry was carried out using an JEOL AccuTOF GCx-plus instrument (JMS-T200GC), fitted with a probe produced by Linden CMS. The probe was equipped with 13 µm emitters on an AccuTOF. Alternatively, LIFDI-MS was carried out using a Waters GCT Premier MS Agilent 7890A GC instrument. Mass to charge ratios (*m/z*) are reported in Daltons. High resolution mass spectra (HRMS) are reported with <5 ppm error (ESI and LIFDI). For clarity, LIFDI data are reported for ^{106}Pd, the most abundant natural isotope of Pd: the 'exact mass'. Infrared spectra were obtained using a Bruker ALPHA-Platinum FTIR Spectrometer with a platinum-diamond ATR sampling module. Elemental analysis (Carbon, Hydrogen and Nitrogen {CHN} content) was carried out on an Exeter Analytical Inc. CE-440 analyser. Fourier-transform attenuated total

reflection (FT-ATR) spectra were recorded on a Bruker ALPHA-Platinum FTIR spectrometer using a platinum-diamond ATR sampling module, reported in terms of frequency of absorption (cm^{-1}).

For single crystal X-ray crystallographic analysis, Suitable single crystals were selected, and diffraction data were collected at 110 K on an Oxford Diffraction SuperNova diffractometer with dual Cu-K_{α} radiation (λ = 1.54184 Å) using a EOS CCD camera. Diffractometer control, data collection, initial unit cell determination, frame integration and unit-cell refinement was carried out with "Crysalis".[4] Using OLEX2[5] software, the structure was solved with the ShelXS327 structure solution program using Direct Methods and refined with the ShelXL refinement package using Least Squares minimisation. Within OLEX2, the algorithm used for structure solution was "ShelXT dual-space".[6] Crystalmaker® software was used to visualise structures as well as for generating the Figure presented herein, and in the main paper. Generally, solvent molecules and counterions have been omitted for clarity, alongside crystallographic data.

2. Experimental Details

2.1. General Procedures

2.1.1. Air sensitive NMR sampling *via* Schlenk Conditions

Two air sensitive techniques were used for NMR sampling:

1. An NMR Schlenk adapter, fitted with a Youngs NMR tube (without lid) was septum-sealed (SubaSeal®) and attached to a Schlenk line before being evacuated and backfilled with N_2 (three times). A sample of the required solution was taken *via* syringe over a septum and swiftly transferred to the NMR Schlenk adapter. Under a strong positive flow of N_2 from the Schlenk line, a Youngs NMR cap was swiftly used to seal the NMR tube (Figure S1). The sample was then subjected to NMR spectroscopic analysis.

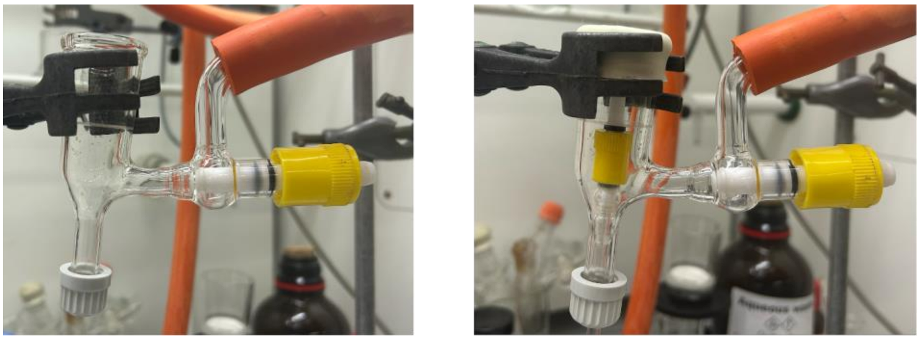

Figure S1: Images demonstrating first setup used for air-sensitive NMR sampling. (Left) Glass Youngs adapter, connected to Schlenk tubing; (Right) The same adapter after sampling, sealed with a capped Youngs NMR tube and a SubaSeal®.

2. A Wilmad® screw-cap NMR tube equipped with a polypropylene cap with PTFE-faced silicone was deoxygenated by sparging with N_2 for ca 30 minutes. A sample of the required solution was taken *via* syringe and transferred to the NMR tube under a positive flow of N_2 (Figure S2). The Sample was then subjected to NMR spectroscopic analysis.

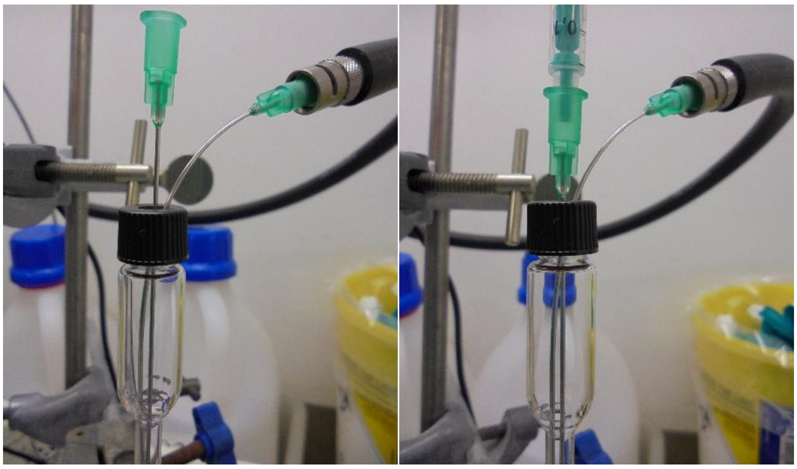

Figure S2: Images demonstrating second setup used for air-sensitive NMR sampling. (Left) NMR tube being deoxygenated by sparging with N_2; (Right) Sample being transferred to NMR tube by syringe.

2.1.2. Suzuki-Miyaura Cross-Coupling Test Reaction Conditions

2.1.2.1. Chlorobenzene/para-fluorophenylboronic acid coupling

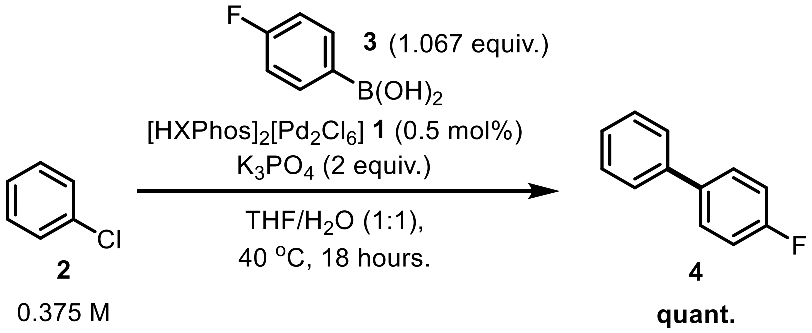

To an oven-dried Schlenk tube, *para*-fluorophenylboronic acid **3** (223 mg, 1.6 mmol, 1.067 equiv.), K_3PO_4 (636 mg, 3.0 mmol, 3.0 equiv.), $[HXPhos]_2[Pd_2Cl_6]$ **1** (10.3 mg, 0.0075 mmol; 0.5 mol%, 1 mol% in Pd) and 1,3,5-trimethoxybenzene (84.1 mg, 0.5 mmol, 0.333 equiv.; internal standard) were added. The flask was sealed (SubaSeal® septum) was evacuated and backfilled with N_2 (three times) before addition of chlorobenzene **2** (152 µL,168 mg, 1.5 mmol, 1.0 equiv). THF (2 mL; dry, degassed) was then added via a syringe over the septum and the subsequent mixture was magnetically stirred for 5 minutes at 40 ^{o}C. After this time, H_2O (2 mL; degassed) was added and the resulting biphasic mixture was stirred at 40 ^{o}C for 18 hours. After this time, the upper THF layer was separated, and the aqueous layer was further extracted with EtOAc (3×5 mL). The combined organics were dried over $MgSO_4$ and filtered over a Celite^{TM} pad (~1.5 cm depth). The filtrate was concentrated in vacuum. The crude product was dissolved in $CDCl_3$ for NMR analysis, which showed quantitative conversion based on comparison of product peaks with that of the 1,3,5-trimethoxybenzene internal standard. This NMR sample was recombined with the rest and the crude product was purified

by flash chromatography (Combiflash®) on silica (dry loaded, eluted with neat hexane). The isolated product 4-fluoro-1,1'-biphenyl appeared as a white powder (258 mg, 84% isolated). See Section 3.1 for Characterization data for the reaction product: 4-fluoro-1,1'-biphenyl (**4**).

2.1.2.2. 4-Fluorobromobenzene/phenylboronic acid coupling. Catalytic activity comparison between $[HXPhos]_2[Pd_2Cl_6]$ and $Pd^{II}I(C_6H_4F)(XPhos)$

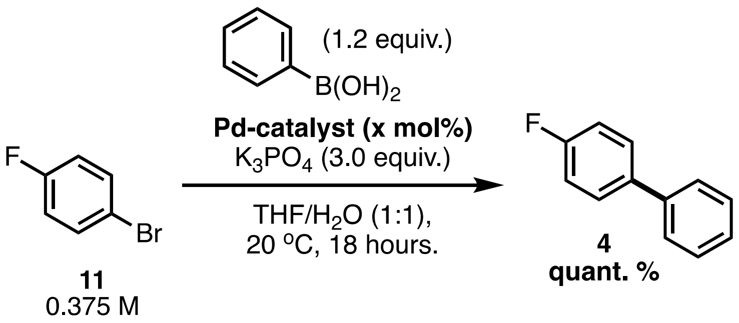

An 8 mL vial equipped with a stir bar and sealed with a polypropylene cap, fitted with PTFE-faced silicone septum was charged with phenylboronic acid (73 mg, 0.6 mmol, 1.2 equiv.), K_3PO_4 (318 mg, 1.5 mmol, 3.0 equiv.), The corresponding Pd-catalyst was added (0.1, 0.25 0.5, 1.0 and 2.0 mol% in Pd, see Table S1).The vial was purged *via* vacuum/N_2 cycles (3 × 1 min each) before the addition of THF (1.5 mL; dry, degassed) and H_2O (1.5 mL; degassed) *via* syringe. The reaction mixture was stirred for 10 minutes at 20 ˚C then 4-fluorobromobenzene (88 mg, 55 µL, 0.5 mmol, 1.0 equiv.) was added to the vial *via* syringe. The reaction was left to stir at 20 ˚C for 18 h. After this time, hexafluorobenzene (93 mg, 55 µL, 0.5 mmol, 1.0 equiv., internal standard) was added to the reaction vial. A sample was taken from the upper layer (THF layer) and analyzed by ^{19}F NMR. The conversion (%) was based on the comparison between the ^{19}F NMR integrations for 4-fluorobromobenzene and for 4-fluoro-1,1'-biphenyl **4**. See Section 3.1 for characterization data for the reaction product: 4-fluoro-1,1'-biphenyl **4**.

Table S1: Quantities used for the catalytic comparison of $[HXPhos]_2[Pd_2Cl_6]$ **1** and Pd(p-C_6H_4F)(I)(XPhos)for the SMCC reaction detailed above.

Pd catalyst (***Pd cat.***)	Pd loading /mol%	Catalyst Loading /mol%	Catalyst Loading /mmol	Catalyst Loading /mg
$[HXPhos]_2$ $[Pd_2Cl_6]$	0.10	0.05	0.0003	0.35
Pd(p-C_6H_4F)(I)(XPhos)	0.10	0.10	0.0005	0.38
$[HXPhos]_2[Pd_2Cl_6]$	0.25	0.13	0.0006	0.86
Pd(p-C_6H_4F)(I)(XPhos)	0.25	0.25	0.0013	0.95
$[HXPhos]_2[Pd_2Cl_6]$	0.50	0.25	0.0013	1.73
Pd(p-C_6H_4F)(I)(XPhos)	0.50	0.50	0.0025	1.90
$[HXPhos]_2[Pd_2Cl_6]$	1.00	0.50	0.0025	3.45
Pd(p-C_6H_4F)(I)(XPhos)	1.00	1.00	0.0050	3.79
$[HXPhos]_2[Pd_2Cl_6]$	2.00	1.00	0.0050	6.91
Pd(p-C_6H_4F)(I)(XPhos)	2.00	2.00	0.0100	7.58

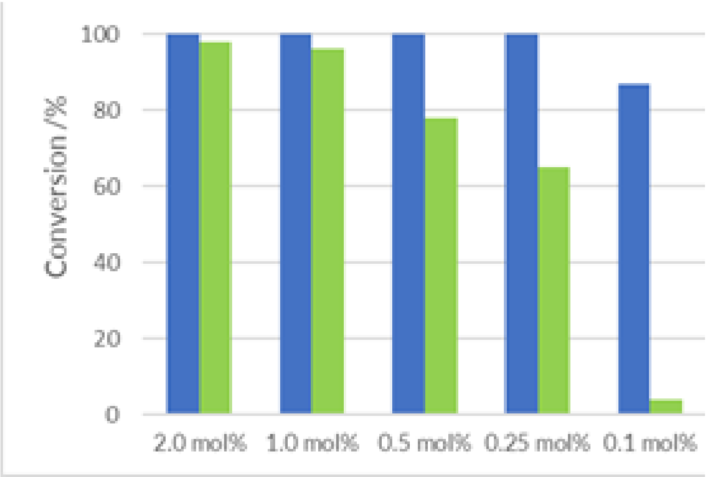

Figure S3: Catalytic activity comparison of $[HXPhos]_2[Pd_2Cl_6]$ (green) and Pd(II)I(C_6H_4F)XPhos (blue) at 2.0–0.1 mol% / Pd loading (% conversion of **11** to **4**, analyzed by ^{19}F NMR spectroscopic analysis).

2.1.3. 5-Bromo-2-chloropyirdine/2-thienyl boronic acid – chemoselective Suzuki–Miyaura coupling

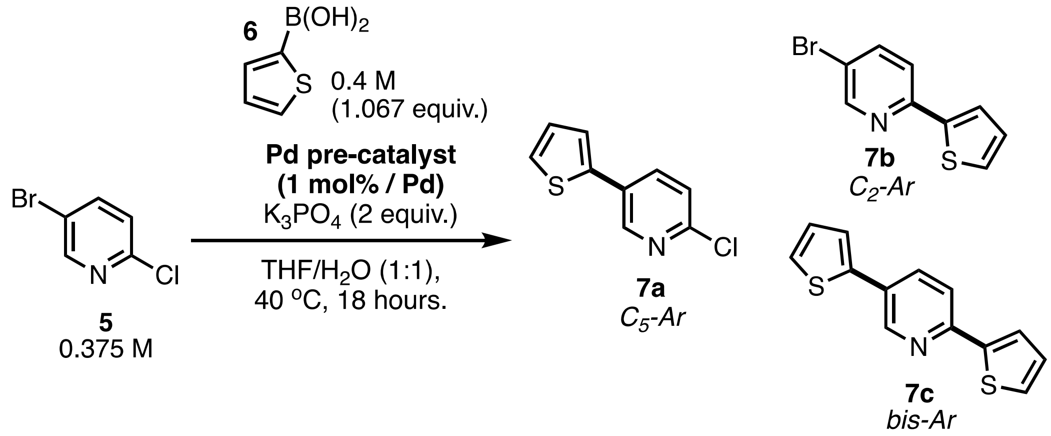

Scheme S1 Chemoselective Suzuki–Miyaura cross-coupling conditions, for catalyst comparison.

To an oven-dried Schlenk tube, 5-bromo-2-chloropyridine **5** (288 mg, 1.5 mmol, 1 equiv.), 2-thienyl boronic acid **6** (204 mg, 1.6 mmol, 1.067 equiv.), K_3PO_4 (636 mg, 3.0 mmol, 3.0 equiv.), the corresponding Pd-catalyst (1 mol% in Pd; Table S2) and 1,3,5-trimethoxybenzene (84.1 mmol, 0.5 mmol, 0.333 equiv.; internal standard) were added. The flask was sealed (SubaSeal® septum) and evacuated and backfilled with N_2 (three times) and THF (2 mL; dry, degassed) was added via a syringe over the septum and the subsequent mixture was magnetically stirred for 5 minutes at 40 °C. H_2O (2 mL; degassed) was then added and the resulting biphasic mixture was stirred at 40 °C for 18 hours. After this time, the upper THF layer was separated and organics were further extracted with EtOAc (3 × 5 mL). The combined organics were dried over $MgSO_4$ and filtered through a Celite™ pad (~1.5 cm depth). The filtrate was concentrated in vacuo. This reaction was analyzed by NMR spectroscopic analysis without further purification (Figure S4). The crude product was dissolved in $CDCl_3$ for analyzed by ^1H NMR spectroscopy against a 1,3,5-trimethoxybenzene internal standard with comparison to literature data and authentic standards of the product (see below).

Table S2: Quantities used for the catalytic comparison of XPhos-containing-pre-catalysts for the SMCC reaction detailed above.

Pd catalyst (***Pd cat.***)	Pd loading /mol%	Catalyst Loading /mol%	Catalyst Loading /mmol	Catalyst Loading /mg
$[HXPhos]_2[Pd_2Cl_6]$	1	0.5	0.0075	10.4
Buchwald-XPhos-Gen3	1	1	0.015	12.7
XPhos Pd(crotyl)Cl	1	1	0.015	10.1
Pd(p-C_6H_4F)(I)(XPhos)	1	1	0.015	12.1

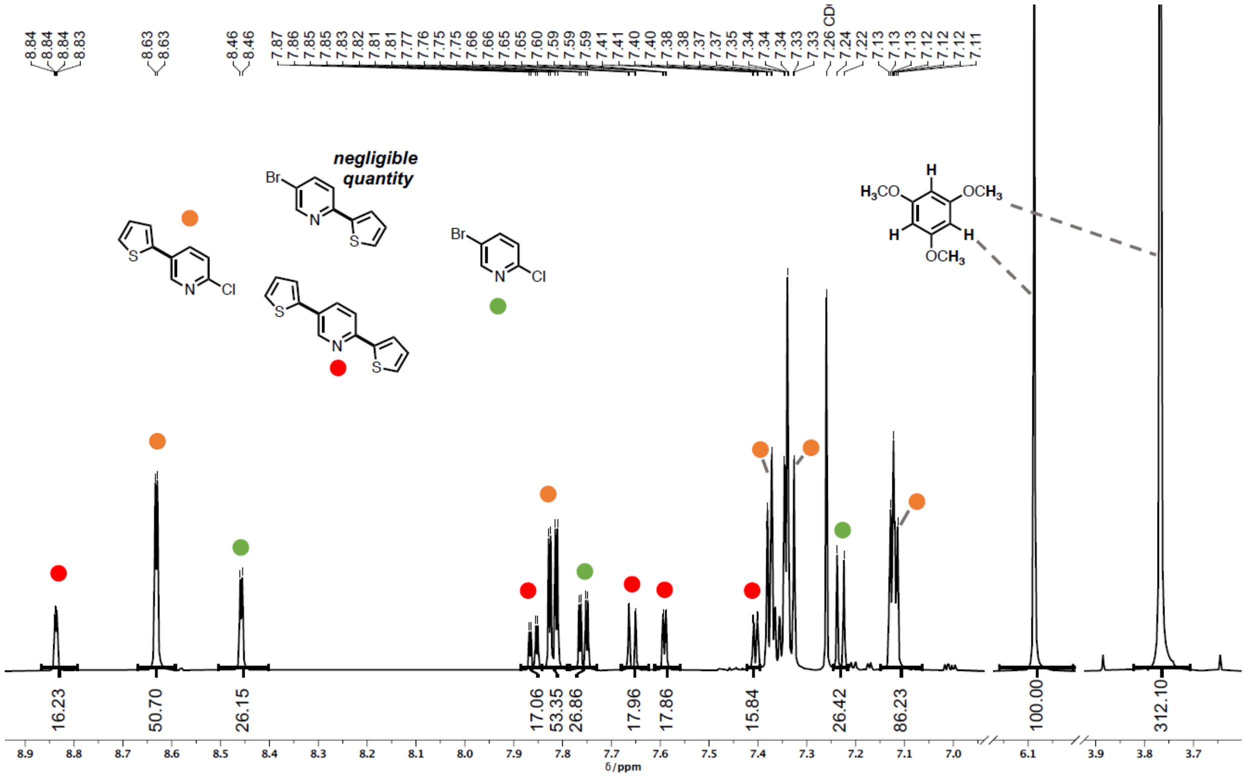

Figure S4: ^{1}H NMR spectrum (600 MHz, $CDCl_3$) allowing analysis of the reaction. Note the cuts to the spectrum, which are in place to allow visualisation of the peaks relevant to reaction quantification.

2.1.3.1. Synthesis of standards for 2,5-disubsituted pyridyl product Identification

2-Chloro-5-(2-thiophenyl) pyridine 7a[7]

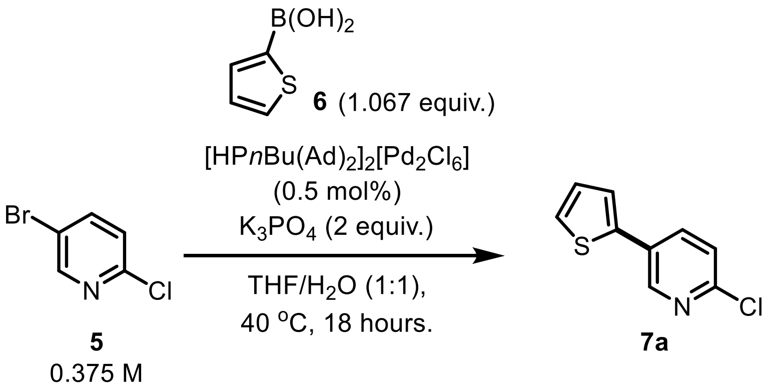

[HP(nBu)(Ad)$_2$]$_2$[Pd$_2$Cl$_6$] was chosen as the pre-catalyst for this process as it was found to be very selective in the activation of the C–Br over the competing C–Cl site of 2-chloro-5-bromopyridine under the following conditions (avoiding the bis-thienylated product).

An oven dried Schlenk flask was charged with [HP(*n*Bu)(Ad)$_2$]$_2$[Pd$_2$Cl$_6$] (11.3 mg, 0.0075 mmol (0.015 mmol in Pd)), 5-chloro-2-bromo-dibromopyridine **5** (289 mg, 1.5 mmol), 2-thiophene boronic acid **6** (1.6 mmol, 1.067 equiv.), K_3PO_4 (636.81, 3.0 mmol, 2 equiv.) and evacuated and backfilled on a Schlenk line. THF (2.5 mL) was added, and the resulting suspension was stirred for 5 mins at 40 ^oC after which time degassed water (2.5 mL) was added. After 18 hours of stirring at 40 ^oC, the aqueous layer was extracted with EtOAc (3 × 5 mL). Organics were combined, dried over $MgSO_4$ and filtered through Celite^{TM} pad (1 cm depth) before being concentrated in vacuo. Product **7a** was isolated as a colourless powder (188.5 mg, 65% yield).

^{1}H NMR (400 MHz, Chloroform-*d*) δ 8.63 (dd, *J* = 2.6, 0.7 Hz, 1H; pyridine H$_6$), 7.82 (dd, *J* = 8.3, 2.6 Hz, 1H), 7.38 (dd, *J* = 5.1, 1.2 Hz, 1H), 7.36 – 7.30 (m, 2H), 7.12 (dd, *J* = 5.1, 3.6 Hz, 1H).^{13}C NMR (101 MHz, Chloroform-*d*) δ 150.06, 146.66, 139.04, 135.85, 129.56, 128.53, 126.56, 124.77, 124.40.HRMS ESI-MS m/z = 195.9982 $[M+H]^+$: C_9H_7ClNS requires; 195.9986 error (ppm) = -1.8. TLC (SiO_2, hexane/Et_2O {9:1}) R_f = 0.22.

5-Bromo-2-(2-thiophenyl) pyridine 7b

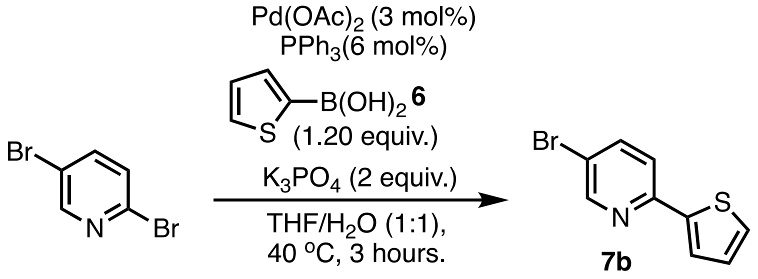

Compound **7b** was synthesised and newly characterized as a standard due to being a novel compound and chromatographic separation issues with the crude reaction; we chose a $Pd(OAc)_2$/2PPh_3 pre-catalyst system to efficiently activate the pyridyl-C_2–Br site of 2,5-dibromopyridine, based on literature precedent. [8]

An oven-dried Schlenk flask was charged with $Pd(OAc)_2$ (10.1 mg, 0.045 mmol), PPh_3 (23.6 mg, 0.090 mmol; 2 equivalents per Pd), 2,5-dibromopyridine (355.5, 1.5 mmol), 2-thiophene boronic acid **6** (1.80 mmol), K_3PO_4 (636.81, 3.0 mmol) and evacuated and backfilled on a Schlenk line. THF (2.5 mL) was added, and the resulting suspension was stirred for 5 mins after which time degassed water (2.5 mL) was added. After 18 hours, the aqueous layer was extracted with EtOAc (3×5 mL). Organics were combined, dried over $MgSO_4$ and filtered through a Celite™ pad (1 cm depth) before being concentrated in vacuo. ^{1}H NMR analysis of this crude material showed three species: C_2–Ar (major), C_5–Ar and diarylated material (^{1}H NMR ratio 1.00:0.09:0.05). The C2-thienylated product **7b**, which appeared as a colourless powder (167.4 mg, 46.5%), could be isolated *via* column chromatography (SiO_2) using a hexane/Et_2O solvent system (10:0.2, v/v).

^{1}H NMR (400 MHz, Chloroform-*d*) δ 8.64 – 8.58 (m, 1H), 7.80 (ddd, *J* = 8.5, 2.4, 0.7 Hz, 1H), 7.59 – 7.52 (m, 2H), 7.42 (dt, *J* = 5.0, 0.9 Hz, 1H), 7.11 (ddd, *J* = 5.0, 3.7, 0.7 Hz, 1H). HRMS ESI-MS m/z = *239.9470* $[M+H]^+$: C_9H_7BrNS requires 239.9477 error (ppm) = *1.8.*
TLC (SiO_2, hexane/Et_2O {10:0.25}) R_f = 0.40.

2,5-Bis-(2-thiophenyl)pyridine 7c [9]

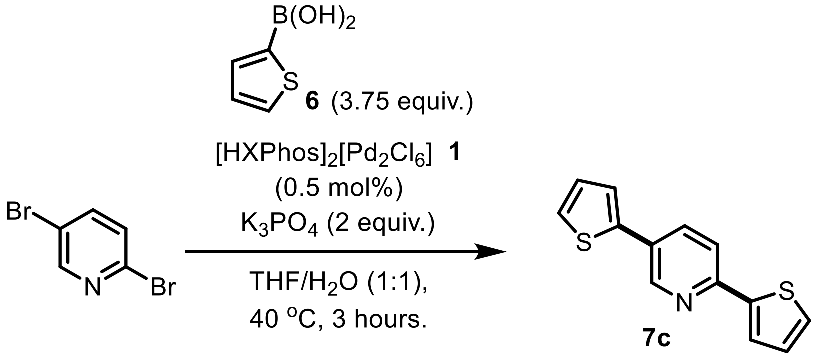

An oven-dried Schlenk flask was charged with $[HXPhos]_2[Pd_2Cl_6]$ **1** (10.3 mg, 0.045 mmol), 2,5-dibromopyridine (237 mg, 1.0 mmol), 2-thiophene boronic acid **6** (479.9, 3.75 mmol, 3.75 equiv.), K_3PO_4 (796.8, 3.75 mmol, 3.75 equiv.) and evacuated and backfilled on a Schlenk line. THF (2.5 mL) was added, and the resulting suspension was stirred for 5 mins after which time degassed water (2.5 mL) was added. After 18 hours, the aqueous layer was extracted with EtOAc (3×5 mL). Organics were combined, dried over $MgSO_4$ and filtered through a Celite™ pad (1 cm depth) before being concentrated in vacuo. The title product, which appeared as a colourless powder (186.4 mg, 77%), could be isolated via column chromatography (SiO_2) using a hexane/EtOAc solvent system (gradient: neat hexane -> hexane/ EtOAc (90:10, v/v).

^{1}H NMR (400 MHz, Chloroform-*d*) δ 8.84 (dd, *J* = 2.4, 0.9 Hz, 1H), 7.87 (dd, *J* = 8.3, 2.4 Hz, 1H), 7.67 (dd, *J* = 8.3, 0.9 Hz, 1H), 7.62 (d, *J* = 3.1 Hz, 1H), 7.41 (dd, *J* = 5.0, 1.1 Hz, 1H), 7.39 – 7.33 (m, 2H), 7.13 (dd, *J* = 5.1, 3.6 Hz, 2H).

^{13}C NMR (101 MHz, Chloroform-*d*) δ 151.66, 146.60, 144.44, 140.49, 133.84, 128.78, 128.50, 128.34, 127.94, 126.00, 124.85, 124.10, 118.91.

HRMS ESI-MS m/z = *244.0245* $[M+H]^+$: $C_{13}H_{10}NS_2$ requires 244.0249 error (ppm) = 1.1.

2.2. Stoichiometric reactivity – Suzuki–Miyaura-type conditions

2.2.1. $[HXPhos]_2[Pd_2Cl_6]$ reaction with K_3PO_4 in THF, with and without *para*-fluorophenyl boronic acid; Identification of *trans*-$[PdCl_2(XPhos)_2]$ as a reaction product.

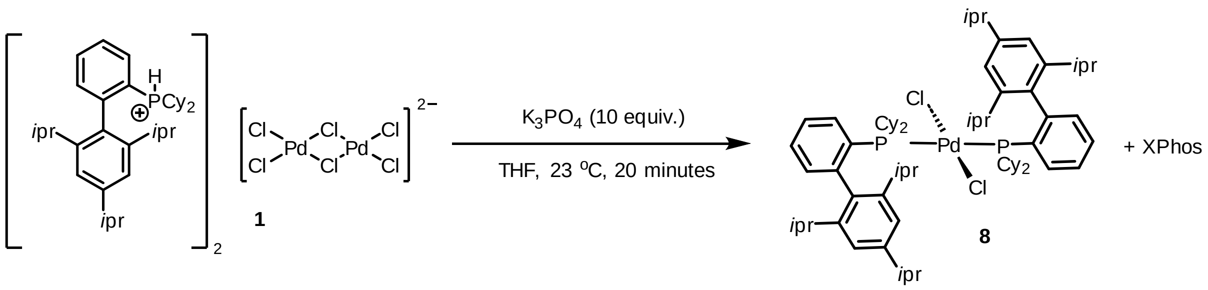

A Schlenk tube was charged with $[HXPhos]_2[Pd_2Cl_6]$ **1** (50 mg, 0.036 mmol) and K_3PO_4 (76 mg, 0.36 mmol; 10 equiv.), *para*-fluorophenyl boronic acid **3** (if applicable: 50 mg, 0.36 mmol; 10 equiv.), before being evacuated and backfilled with N_2 (three times). THF (2 mL; dry degassed) was added and the reaction was stirred for 20 minutes. After this time, a sample of the solution was taken *via* Schlenk, air-sensitive NMR sampling (See Section 2.1.1). NMR Characterization data matched that of literature and a synthesised authentic standard (see section 3.4 for further details). Crystals of complex **8** could be grown by vapour diffusion of pentane onto a benzene solution of the reaction product. To complement the paper, results are shown below in Figure S5, below, which indicate the formation of *trans*-$[PdCl_2(XPhos)_2]$ **8** and free XPhos (ligand) as major products of these reactions.

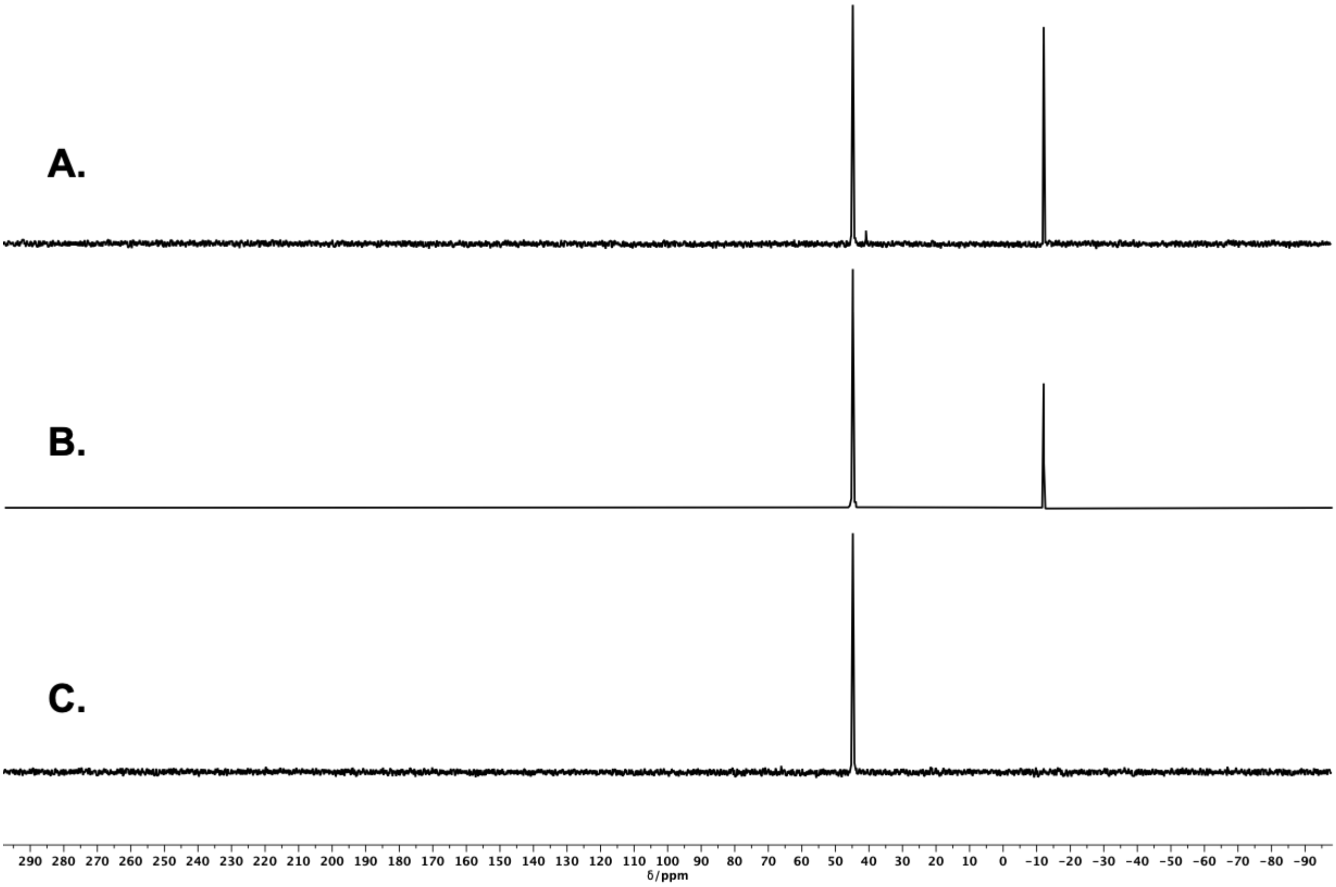

Figure S5: ^{31}P NMR (242 MHz, THF, unlocked) stack of: A. Reaction of $[HXPhos]_2[Pd_2Cl_6]$ **1** with K_3PO_4 (10 equiv.), B. the same reaction but with added *para*-fluorophenylboronic acid **3** (10 equiv.); C. authentic *trans*-$[PdCl_2(XPhos)_2]$ **8**.

2.2.2. Solubility studies of *trans*-[$PdCl_2(XPhos)_2$] (8)

Two separate 4 mL vials equipped with a magnetic stirrer bar, and a polypropylene cap with PTFE-faced silicone septum, were loaded each with *trans*-[$PdCl_2(XPhos)_2$] **8** (56 mg, 0.05 mmol). This was followed by the addition of THF (1.5 mL) to the first vial and THF (1.5 mL) and H_2O (1.5 mL) to the second vial. The two vials were stirred for 30 minutes. After this time, triphenyl phosphine oxide was added to each vial as internal standard (14 mg, 0.05 mmol, 1.0 equiv.). A sample was taken from the first vial and analyzed by ^{31}P NMR {H} (D1 = 60 s). ^{31}P NMR indicated *ca.* 20% **8** in solution. The upper phase of the second vial was also sampled and analyzed by ^{31}P {H} NMR (D1 = 60 s). ^{31}P NMR spectroscopic analysis indicated the there was no remaining **8** present in solution.

2.2.3. [$HXPhos]_2[Pd_2Cl_6$] 1 reaction with K_3PO_4 and *para*-fluorophenyl boronic acid 3 in THF

To an oven-dried Schlenk Flask was added [$HXPhos]_2[Pd_2Cl_6$] **1** (50 mg, 0.036 mmol; 1 equiv.), *para*-fluorophenylboronic acid **3** (51 mg, 0.36 mmol; 10 equiv.) and K_3PO_4 (76 mg, 0.36 mmol; 10 equiv.) which was septum-sealed (SubaSeal) before being evacuated and backfilled with N_2 (three times). THF (2 mL; dry, degassed) was added via a syringe over the septum and the resulting mixture was magnetically-stirred at room temperature for 5 minutes. Over this time, the mixture – initially orange in colour – was seen to turn yellow, with a stirred suspension of undissolved white solid. Here, only *trans*-$PdCl_2(XPhos)_2$ **8** was detectable by ^{31}P NMR spectroscopic analysis.

2.2.4. [HXPhos]$_2$[Pd$_2$Cl$_6$] 1 reaction with K$_3$PO$_4$ and *para*-fluorophenyl boronic acid in THF/H$_2$O

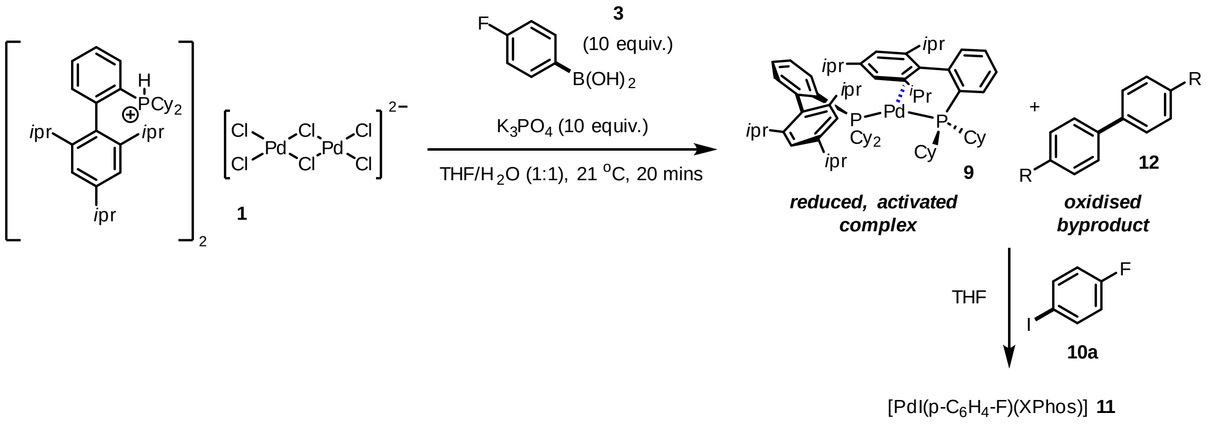

Part 1 – Generation of Pd^0(XPhos)$_2$ complex

To an oven-dried Schlenk Flask was added [HXPhos]$_2$[Pd$_2$Cl$_6$] **1** (50 mg, 0.036 mmol; 1 equiv.), *para*-fluorophenylboronic acid **3** (51 mg, 0.36 mmol; 10 equiv.) and K$_3$PO$_4$ (76 mg, 0.36 mmol; 10 equiv.) which was septum-sealed (SubaSeal) before being evacuated and backfilled with N$_2$ (three times). THF (2 mL; dry, degassed) was added *via* a syringe over the septum and the resulting mixture was magnetically-stirred at room temperature for 5 minutes. Over this time, the mixture - initially orange in colour, was seen to turn yellow – with a stirred suspension of undissolved white solid. H$_2$O (2 mL, degassed) was added and the reaction mixture, observed to immediately darken, was rapidly magnetically stirred at room temperature for 20 minutes. After this time, prior to sampling, the biphasic solution (two solution phases) was allowed to settle; the upper phase was dark (almost black), with a slight greenish tinge while the lower phase was dark orange. The upper phase was sampled air-sensitively on the Schlenk line according to the procedure detailed in Section 2.1.1 for NMR analysis (care must be taken to ensure good phase partition prior to sampling). A like sample was analyzed (ca. 0.5 mL) by LIFDI-mass analysis, showing several intense ions including one at m/z 1058.60405, matching the chemical formula $C_{66}H_{98}P_2Pd$ (calc. 1058.61766), corresponding to the radical cation ([M]·+), [Pd(XPhos)$_2$] (see the main paper for further details). Ions at m/z 476.35719 and m/z 433.29199 were assigned as free XPhos ([M]·; $C_{33}H_{49}P$; m/z 476.35719) and ([M–isopropyl]$^{\cdot+}$; $C_{30}H_{42}P$; m/z 433.30241), respectively.

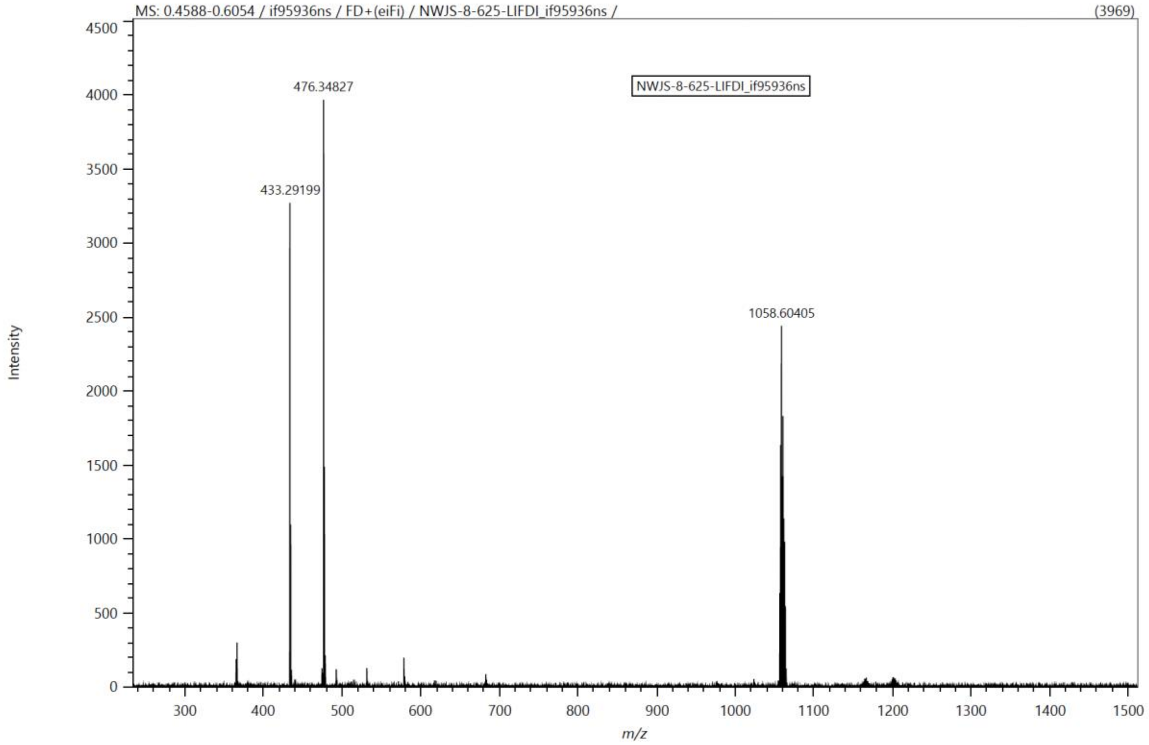

Figure S6: LIFDI-MS spectrum showing radical cation representative of [$Pd^0(XPhos)_2$] **9**.

Part 2 - Addition of para-fluoroiodobenzene

After initial NMR spectroscopic analysis, under Schlenk conditions (Section 2.1.1), *para*-fluoroiodobenzene **3** (ca. 10 μL; excess) was added to the NMR tube and the same NMR tube was sealed and analyzed by NMR spectroscopy (Figure S7). The same sample was subjected to LIFDI-MS analysis (Figure S8). ^{31}P NMR data, supported by LIFDI-MS data, indicated that oxidative addition of *para*-fluoroiodobenzene **10a** to [$Pd^0(XPhos)_2$] **9**, forming [Pd^{II}(I)(*p*-C_6H_4F)(XPhos)] **11**, with ^{31}P NMR data matching that of literature and the authentic standard synthesized in this study (Section 2.2.14).[10]

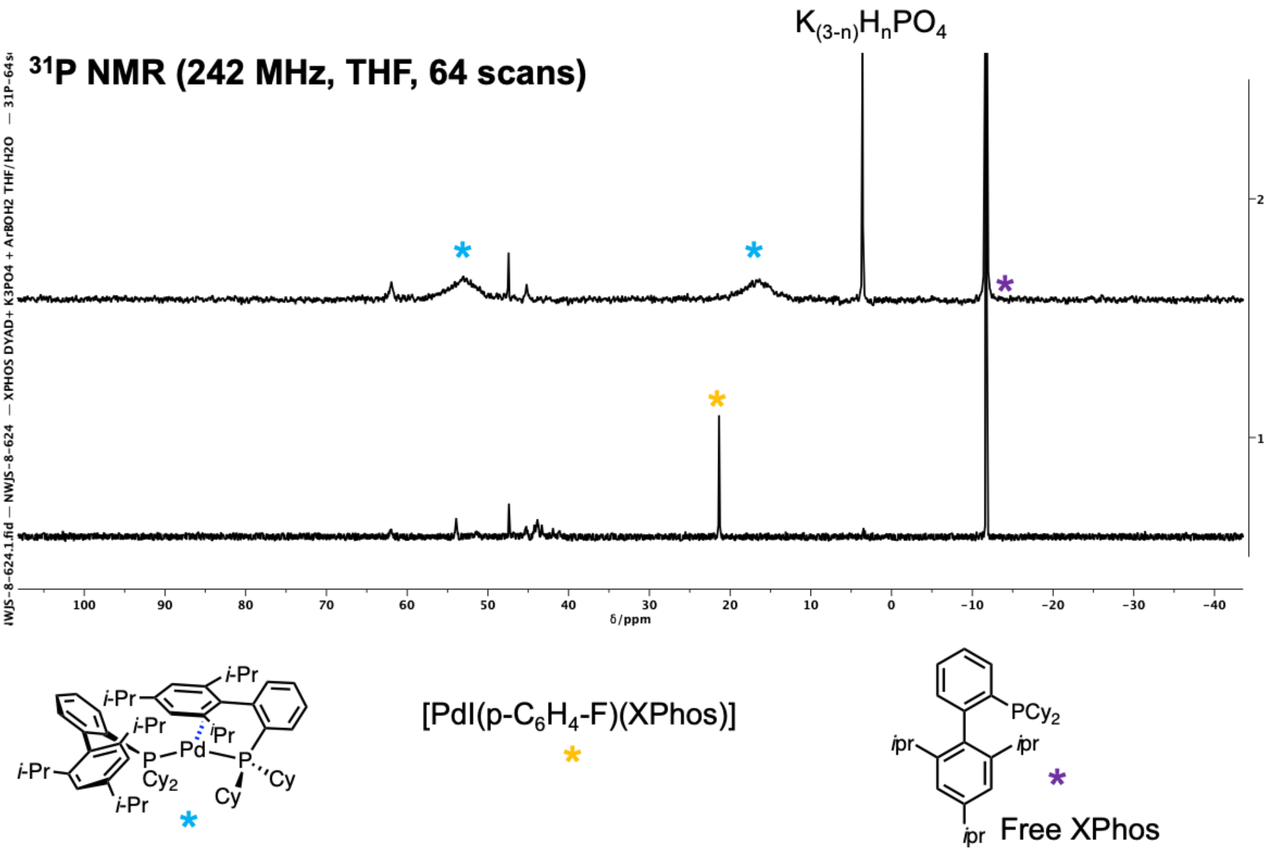

Figure S7: ^{31}P NMR stacked spectra showing reaction of $Pd^0(XPhos)_2$ **9** with *para*-fluoroiodobenzene **10a**.

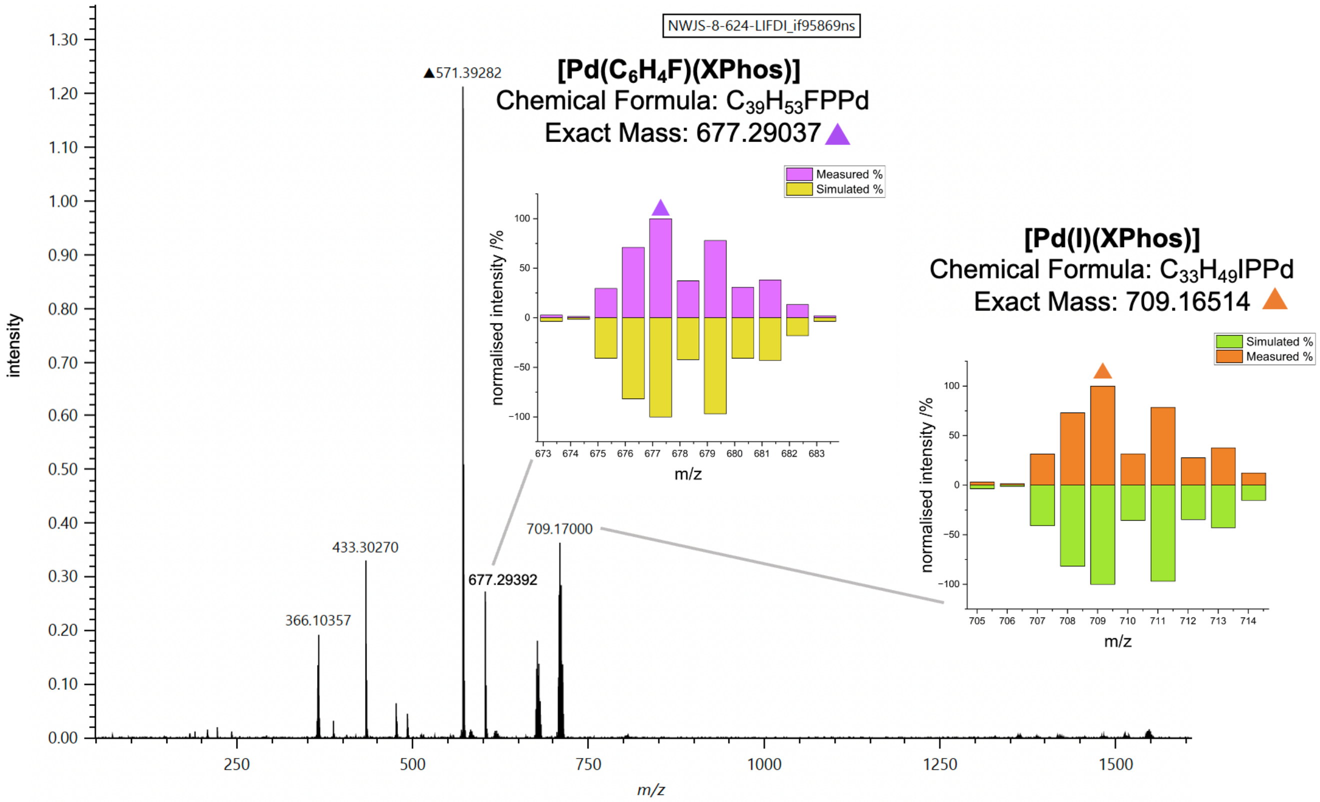

Figure S8: LIFDI-MS spectrum of the solution following activation of $[HXPhos]_2[Pd_2Cl_6]$ **1** and subsequent reaction with *para*-fluoroiodobenzene **10a**.

2.2.5. Homocoupled product spiking experiment

Procedure: to an NMR sample containing the contents of the solution after *Part 1*, Section 2.2.4, a 5 mg sample of 4,4'-difluorobiphenyl **12** was added directly to the NMR tube. ^{19}F NMR spectroscopic analysis was used to determine unequivocally that the peak at δ_F –116.9 ppm was 4,4'-difluorobiphenyl **12**, identified due to its qualitative change in integration directly after spiking (Figure S9). This experiment confirms that 4,4'-difluorobiphenyl **12** is a product of the $[HXPhos]_2[Pd_2Cl_6]$ **1** reaction with *para*-fluorophenylboronic acid **3** as the activating agent.

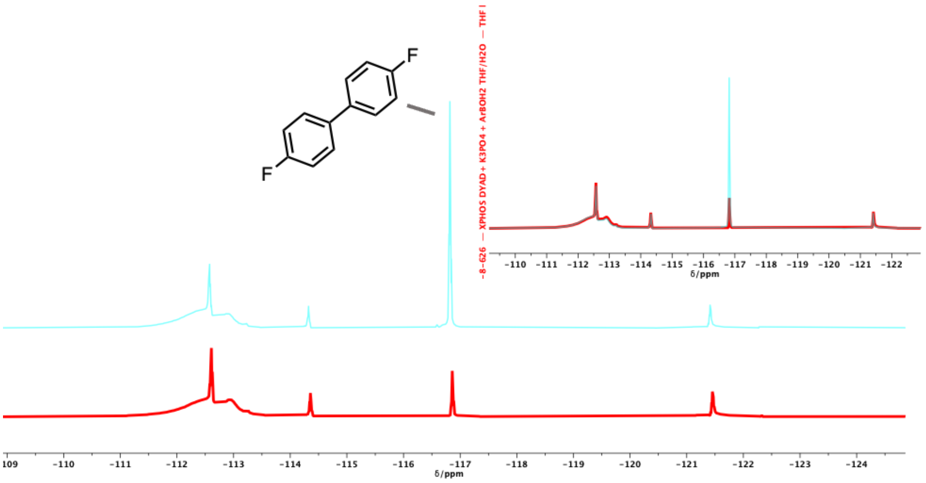

Figure S9: Showing the change in integration of the peak representative of 4,4'-difluorobiphenyl **12** after spiking with an authentic standard. Bottom red spectrum – before the addition of 4,4'-difluorobiphenyl, Top light blue spectrum – after the addition of an authentic sample of 4,4'-difluorobiphenyl.

2.2.6. Identification of $K_nH_{3-n}PO_4$ in the reaction mixture.

A control experiment was designed to confirm identity of the recurrent ^{31}P NMR species at δ_P 3.2 in spectra involving combinations of $[HXPhos]_2[Pd_2Cl_6]$ **1** and K_3PO_4, in combination with other species. Three separate reactions were set up and to each solids (see below for quantities) were added to the Schlenk tube, followed by THF (2 mL) with conditions mirroring the concentrations of the Dyad salt activation experiments (see above). Samples (0.5 mL) were taken from the upper layer of each and promptly analyzed by ^{31}P NMR. The first Schlenk tube (**A**) was charged with K_3PO_4 (76 mg, 0.36 mmol). A second Schlenk tube (**B**) was charged with K_3PO_4 (76 mg, 0.36 mmol; 10 equiv.) and $[HXPhos]_2[Pd_2Cl_6]$ **1** (50 mg, 0.036 mmol; 1 equiv.) (**B**, Figure S10). A third Schlenk tube (**C**) was charged with K_3PO_4 (76 mg, 0.36 mmol), $[HXPhos]_2[Pd_2Cl_6]$ **1** (50 mg, 0.036 mmol; 1 equiv.) and *para*-fluorophenylboronic acid **3** (51 mg, 0.36 mmol)(**C**, Figure S10). The experiment shows that K_3PO_4 exhibits a peak at δ_P 5.1

ppm in the top layer of a THF/H_2O (1:1) medium (**C**, Figure S10). The combination of K_3PO_4 and $[HXPhos]_2[Pd_2Cl_6]$ **1** in the same medium resulted in upfield peak migration to δ_P 4.0 ppm (**B**, Figure S10), migrating further to δ_P 3.2 ppm in the presence of *para*-fluorophenylboronic acid (**C**, Figure S10). Hence, as acidic components ($[HXPhos]_2[Pd_2Cl_6]$; *para*-fluorophenyl boronic acid **3**) were incrementally added this peak was seen to migrate upfield, this is consistent with progressive acidification the $[PO_3]^{2-}$ anion, forming $K_{(3-n)}H_nPO_4$ in progression towards H_3PO_4 (δ_P 0 ppm) in the reaction medium.

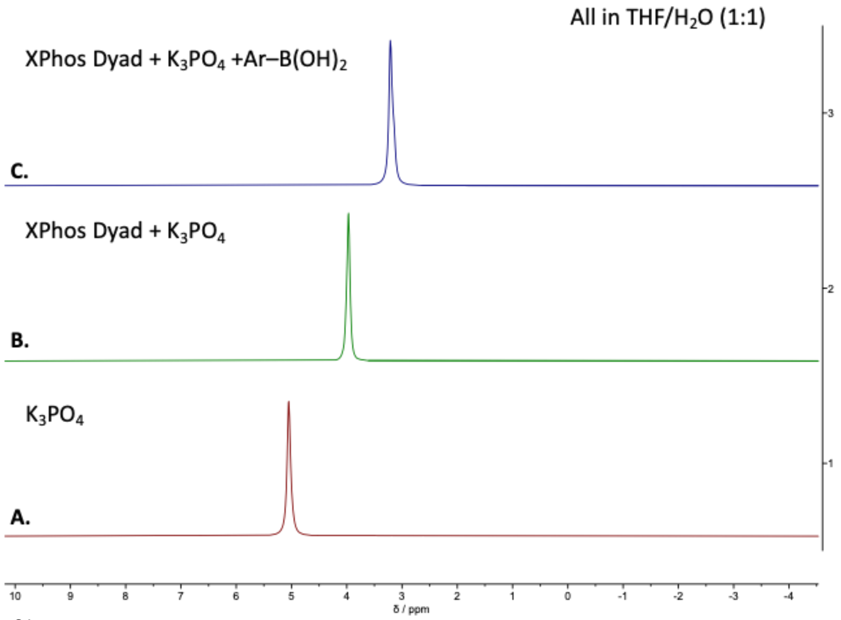

Figure S10: ^{31}P NMR 242 MHz of samples from control experiments focusing on phosphate chemical shift identification.

2.2.7. Generation of $Pd^0(XPhos)_2$ 9 in presence of differing arylboronic acids

For experiments examining the reduction of $[HXPhos]_2[Pd_2Cl_6]$ **1** in the presence of differing substituted phenylboronic acids, the same general procedure from Section 2.2.4 was followed. However, in this case substituting the *para*-fluorophenyl boronic acid **3** for the same molar equivalence (10 equivalents per $[HXPhos]_2[Pd_2Cl_6]$ **1**) using either *para*-methoxyphenylboronic acid or *para*-trifluoromethylphenylboronic acid. In these cases, $[Pd^0(XPhos)_2]$ complex **9** was seen to form in both cases, characterized by ^{31}P NMR as broad resonances at δP 16.7 and 55.2 ppm.

2.2.8. $[HXPhos]_2[Pd_2Cl_6]$ activation using various bases

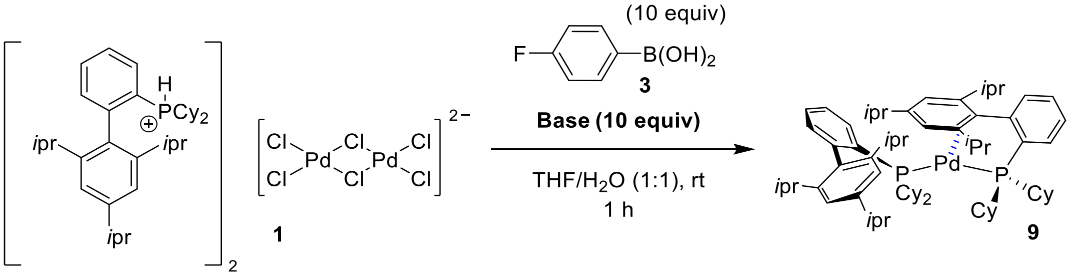

A 4 mL vial was equipped with a magnetic stirrer bar and a polypropylene cap fitted with a with PTFE-faced silicone septum. It was charged with $[HXPhos]_2[Pd_2Cl_6]$ **1**, the desired base and *para*-fluorophenylboronic acid **3**. The vial was deoxygenated *via* vacuum/N_2 cycles (3 × 1 min each). After THF (degassed and dry) and H_2O (degassed), were successively added to the vial *via* syringe, the reaction mixture was stirred for 30 minutes before a sample was charged into a deoxygenated NMR tube and analyzed by ^{31}P NMR, with $Pd^0(XPhos)_2$ **9** being detected or not by ^{31}P NMR, as broad resonances at δ_P 16.7 and 55.2 ppm (Table S3).

Table S3: Investigation of base effect for the reduction of $[HXPhos]_2[Pd_2Cl_6]$ 1 to generate $Pd^0(XPhos)_2$ [a]

Entry	Base	Generation of complex 3
1	K_3PO_4	✓
2	Na_2CO_3	✓
3	NaOH	×
4	NaOAc	×
5	CsOPiv	×

[a] General conditions: 0.027 mmol $[HXPhos]_2[Pd_2Cl_6]$, 0.27 mmol base, 0.27 mmol *para*-fluorophenylboronic acid **3**, 1.5 mL THF, 1.5 mL H_2O, 20 °C, 30 min. Due to the nature of the broad peaks, consistent with complex **9**, line broadening (set to 5) was used during the NMR spectral processing.

2.2.9. Synthesis of $Pd(XPhos)_2$ by reaction of [$Pd(CH_2SiMe_3)_2$(1,5-cyclooctadiene)] with XPhos in THF

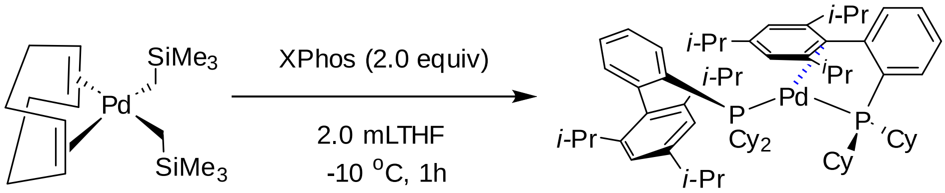

A 8 mL vial equipped with a stir bar and a polypropylene cap fitted with a PTFE-faced silicone septum was loaded with bis(trimethylsilylmethyl)-(cycloocta-1,5-diene) palladium(II) (54 mg, 0.139 mmol, 1.0 equiv.) and XPhos (132 mg, 0.278 mmol, 2.0 equiv.). The vial was sealed and was evacuated and backfilled with N_2 (three times) before addition of THF (2.0 mL; dry, degassed). The vial was cooled to –10 ˚C and the reaction mixture was left to stir for 1h. After this time a bright-green solution was observed. A Wilmad® screw-cap NMR tube equipped with a polypropylene cap with PTFE-faced silicone was deoxygenated by sparging with N_2 for ca 30 minutes. A sample of the reaction mixture was charged into the degassed NMR tube *via* syringe and the sample was analyzed by ^{31}P NMR spectroscopic analysis without further purification (Figure S11).

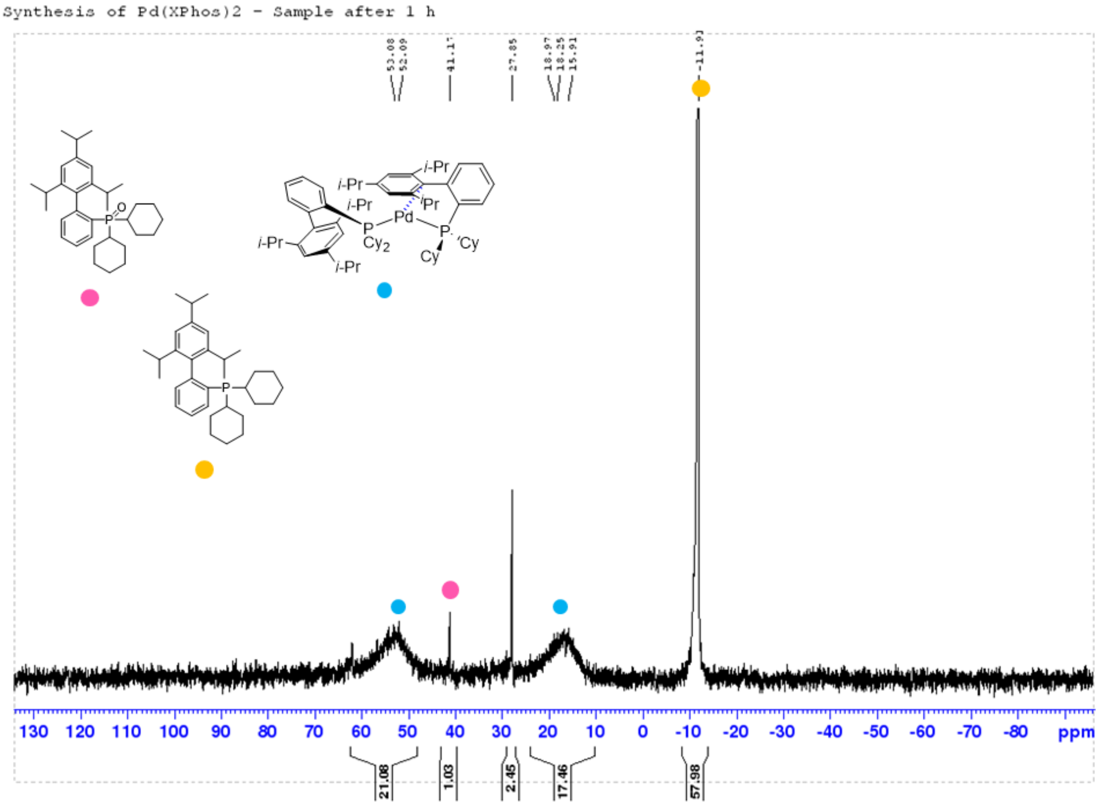

Figure S11: ^{31}P NMR spectrum (162 MHz, THF) allowing analysis of the reaction between $Pd(CH_2Si(CH_3)_3)_2$(1,5-cyclooctadiene) and XPhos.

2.2.10. Variable Temperature (VT) ^{31}P NMR experiment

In an Ar-filled glovebox, XPhos (12.3 mg, 0.026 mmol, 2 equiv.) was weighed into a 2.0 mL vial and dissolved in THF (0.5 mL; dry, degassed). [Pd(CH_2SiMe_3)$_2$(1,5-cyclooctadiene)] (5 mg, 0.013 mmol, 1 equiv.) (stored at –30 °C) was swiftly weighed into a separate 2.0 mL vial and the THF solution of XPhos was added, with immediate vigorous shaking. The light-yellow solution was transferred into a Youngs NMR tube which was sealed, removed from the glovebox and promptly analyzed by NMR spectroscopic analysis with the first data collection (^{31}P NMR, 202 MHz, 64 scans, 298 K) approximately 10 minutes after the reaction initiation. Subsequent spectroscopic analyses (^{31}P NMR, 202 MHz, 64 scans) were collected at 298, 283, 278, 273, 268, 263, 253 and 243 K (Figure S12).

Notes and Observations:

- Peaks representative of different ^{31}P environments of [Pd^0(XPhos)$_2$] were seen to resolve into two doublets δ_P 16.7 and 55.2 ppm with matched coupling constants of $J_{P–P}$ 235 Hz.
- In addition of XPhos and XPhos oxide several unknown species were detected (resonances at 29.1, 28.0 and 26.2 ppm were detected) these may constitute intermediates en route to [Pd^0(XPhos)$_2$] product formation.
- The peak representative of free XPhos was seen to migrate upfield as a function of reduced temperature (–11.7 to –12.5 ppm over the temperature range examined).

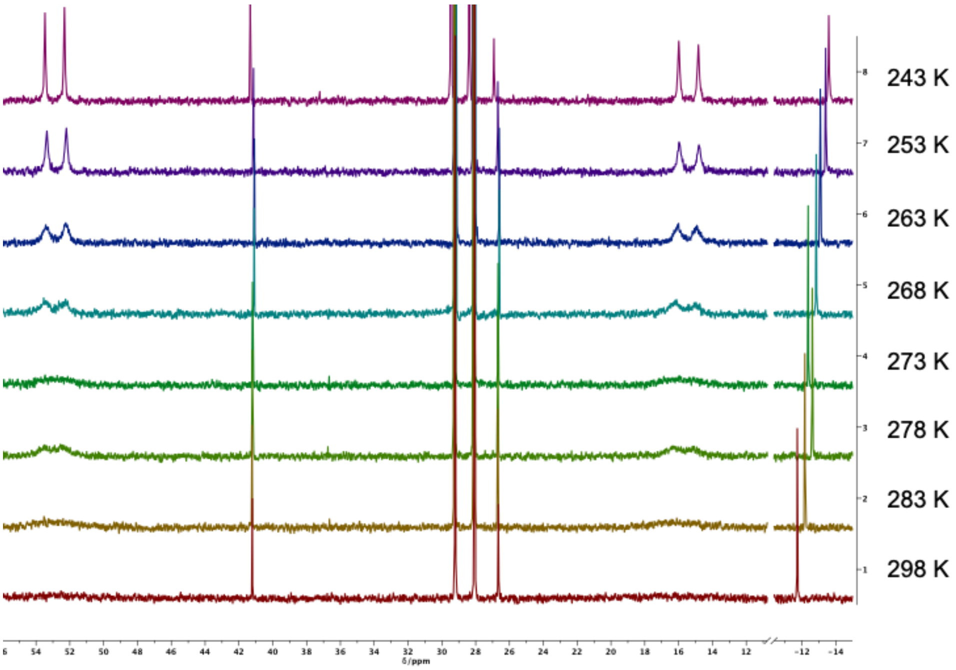

Figure S12: Stacked spectra from ^{31}P VT NMR experiment, showing resolution of dynamic behaviour at low temperature.

2.2.12 Quantification of oxidative addition complex 11' generated from $[HXPhos]_2[Pd_2Cl_6]$ 1 under Suzuki–Miyaura-type reaction conditions

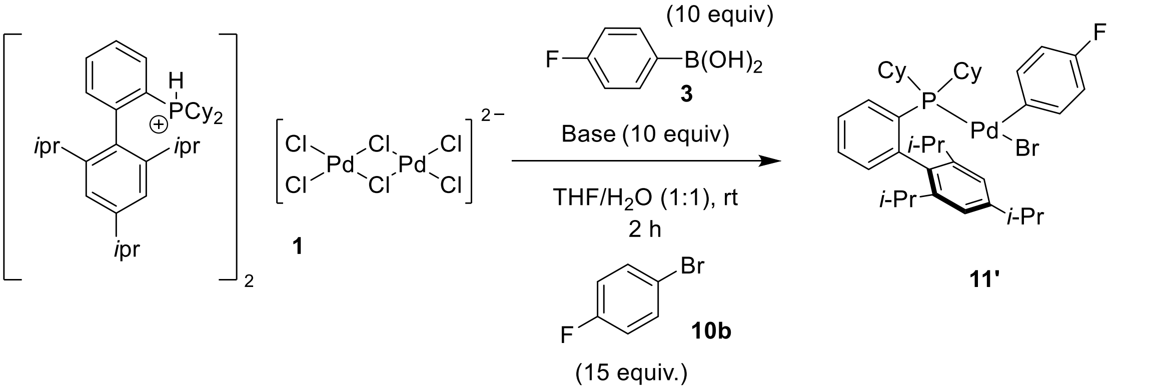

A 20 mL Schlenk flask equipped with a stir bar and a septum was charged with $[HXPhos]_2[Pd_2Cl_6]$ **1** (37 mg, 0.03 mmol, 1.0 equiv.), the corresponding base (0.27 mmol, 10 equiv.; See Table S4 for the identity of base) and *para*-fluorophenylboronic acid **3** (33 mg, 0.27 mmol, 10 equiv.). The vial was deoxygenated *via* vacuum/N_2 cycles (3×1 min each). After, *para*-fluorobromobenzene **10b** (71 mg, 45 µL, 0.41 mmol, 15 equiv.) was added to the flask followed by the addition of THF (1.5 mL; dry, degassed) and H_2O (1.5 mL; degassed) *via* syringe. The reaction mixture was left to stir for 2h. After this time, a degassed solution of triphenylphosphine oxide (10 mg, 0.035 mmol, 1.3 equiv., in 0.5 mL THF; internal standard) was added to the flask. A Wilmad® screw-cap NMR tube equipped with a polypropylene cap with PTFE-faced silicone was deoxygenated by sparging with N_2 for ca 30 minutes. A sample of the reaction mixture was charged into the deoxygenated NMR tube *via* syringe and the sample was analyzed by $^{31}P\{H\}$ NMR (d1 = 30s) spectroscopic analysis without further purification.

Table S4: Observed oxidative addition complex formation using Suzuki-Miyaura reaction conditions with various inorganic bases.

Entry	Base	Generation of complex 2
1	K_3PO_4	21%
2	Na_2CO_3	53%
3	NaOH	16%

[a] General conditions: 0.03 mmol $[HXPhos]_2[Pd_2Cl_6]$ **1**, 0.27 mmol base, 0.27 mmol *para*-fluorophenylboronic acid **3**, 1.5 mL THF, 1.5 mL H_2O, 0.41 mmol 4-fluoro-bromobenzene **11**, 20 °C, 2h. Due to the nature of the peaks consistent with complex **2**, line-broadening (5) was used during the spectral processing to improve signal-to-noise ratio.

2.2.13. Quantification of oxidative addition complex generated after activation of $[PdCl_2(XPhos)_2]$ 8 under Suzuki–Miyaura-type reaction conditions

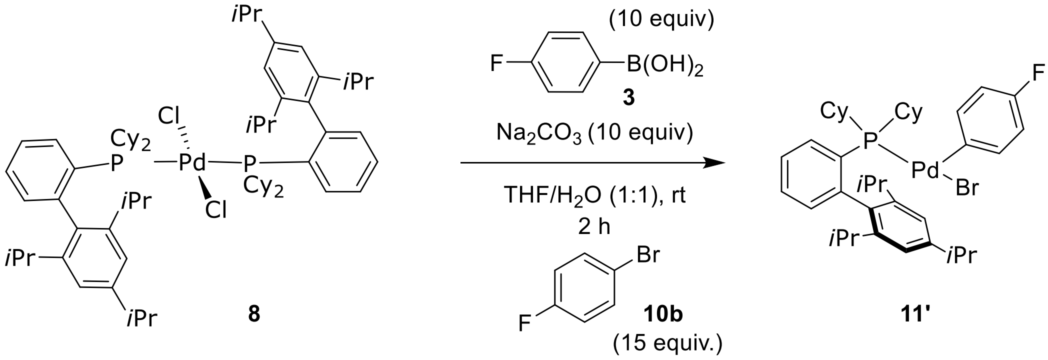

A 20 mL Schlenk flask equipped with a stir bar and a septum was charged with *trans*-$[PdCl_2(XPhos)_2]$ **8** (61 mg, 0.05 mmol, 1.0 equiv.), Na_2CO_3 (57 mg, 0.54 mmol, 10 equiv.) and *para*-fluorophenylboronic acid **3** (66 mg, 0.54 mmol, 10 equiv.). The vial was deoxygenated *via* vacuum/N_2 cycles (3×1 min each). After, 4-fluoro-bromobenzene **10b** (142 mg, 89 µL, 0.81 mmol, 15 equiv.) was added to the flask followed by the addition of THF (3.0 mL; dry, degassed) and H_2O (3.0 mL; degassed) *via* syringe. The reaction mixture was left to stir for 2h. After this time, a degassed solution of triphenyl phosphine oxide (13.9 mg, 0.05 mmol, 1.0 equiv., in 0.5 mL THF, internal standard) was added to the flask. A Wilmad® screw-cap NMR tube equipped with a polypropylene cap with PTFE-faced silicone was deoxygenated by sparging with N_2 for ca 30 minutes. A sample of the reaction mixture was charged into the degassed NMR tube *via* syringe and the sample was analyzed by $^{31}P\{H\}$ NMR (d1=30s) spectroscopic analysis without further purification. A 2% yield of the oxidative addition product was observed by NMR by comparison with the internal standard.

2.2.14. Synthesis of [Pd^{II}(I)(*p*-C_6H_4F)(XPhos)] as an authentic standard

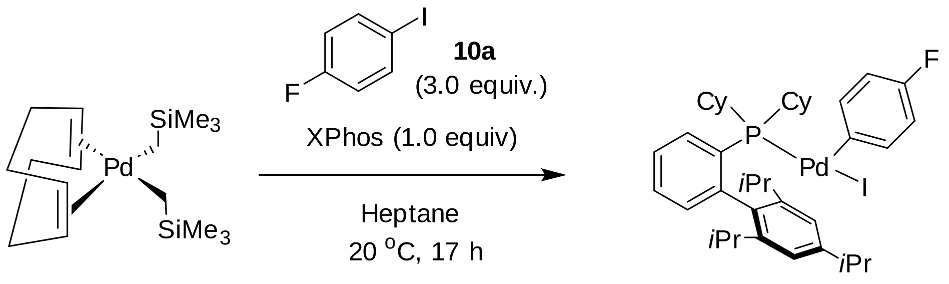

11

A 20 mL Schlenk flask equipped with a stir bar and a septum was charged with XPhos (610 mg, 1.29 mmol, 1.0 equiv.). The flask was deoxygenated *via* vacuum/N_2 cycles (3×1 min each). After, heptane (30 mL) and 4-fluoroiodobenzene **10a** (86 mg, 44 µL, 3.86 mmol, 3.0 equiv.) The white suspension was stirred for 10 minutes at 20 ˚C before adding bis(trimethylsilylmethyl)-(cycloocta-1,5-diene) palladium(II) (500 mg, 1.28 mmol, 1.0 equiv.) This reaction mixture was stirred under N_2 for 17 h at 20 ˚C. After this time a white yellow solution with off-white precipitate was observed. The solid was isolated by vacuum filtration and washed with heptane (3 × 10 mL) to give an off-white powder (770 mg, 75%), matching the literature data for this compound.[10]

2.2.15. $[HXPhos]_2[Pd_2Cl_6]$ 1 reaction with K_3PO_4 and *para*-fluorophenyl boronic acid in THF at 60 ˚C.

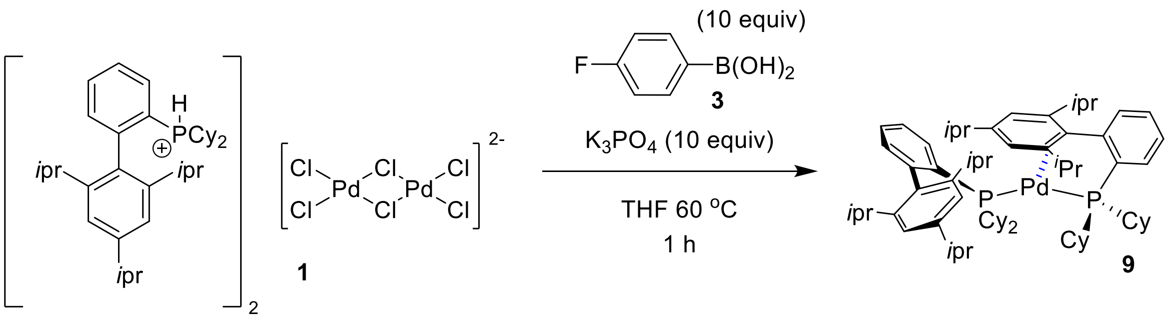

A 4 mL vial equipped with a stir bar and a polypropylene cap fitted with a with PTFE-faced silicone septum was charged with $[HXPhos]_2[Pd_2Cl_6]$ **1** (37 mg, 0.03 mmol, 1.0 equiv.), K_3PO_4 (57 mg, 0.27 mmol, 10 equiv.) and *para*-fluorophenylboronic acid **3** (33 mg, 0.27 mmol, 10 equiv.). The vial was deoxygenated *via* vacuum/N_2 cycles (3 × 1 min each). After, THF (1.5 mL; dry, degassed) was added to the vial *via* syringe. The reaction mixture was left to stir for 1h at 60 ˚C. A Wilmad® screw-cap NMR tube equipped with a polypropylene cap with PTFE-faced silicone was deoxygenated by sparging with N_2 for ca 30 minutes. A sample of the reaction mixture was charged into the degassed NMR tube *via* syringe and the sample was analyzed by $^{31}P\{H\}$ NMR spectroscopic analysis without further purification.

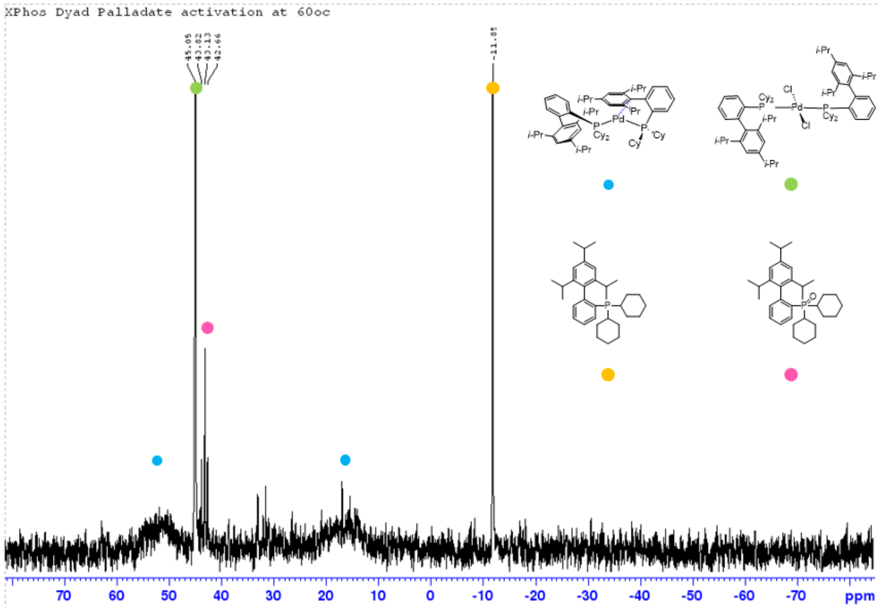

Figure S13: ^{31}P NMR spectrum (THF, 162 MHz) showing molecular speciation upon reaction between $[HXPhos]_2[Pd_2C_{l6}]$ **1** and *para*-fluorophenylboronic acid **3** under the above conditions.

2.3. Stoichiometric reactivity – Buchwald–Hartwig-type conditions

2.3.1. Reaction of $[HXPhos]_2[Pd_2Cl_6]$ 1 with NaO*t*Bu (A, Figure 5 main paper)

A Schlenk tube charged with $[HXPhos]_2[Pd_2Cl_6]$ **1** (50 mg, 0.036 mmol) and NaO*t*Bu (10 equiv.) was evacuated and backfilled with N_2 (3 times). THF (2 mL dry, degassed) was added, and the resulting mixture was magnetically stirred at room temperature for 20 minutes. After this time, a sample was taken for NMR analysis according to the procedure described in Section 2.1.1.

2.3.2. Reaction of $[HXPhos]_2[Pd_2Cl_6]$ 1 with morpholine (B, Figure 5, main paper)

A Schlenk tube charged with $[HXPhos]_2[Pd_2Cl_6]$ **1** (50 mg, 0.036 mmol) was evacuated and backfilled with N_2 (3 times). THF (2 mL dry, degassed) was added, followed, 1 minute later by morpholine (31 uL, 0.36 mmol; 10 equivalents), and the resulting mixture was magnetically stirred at room temperature for 20 minutes. After this time, a sample was taken for NMR analysis according to the procedure detailed in Section 2.1.1.

The solution was concentrated *in vacuo* and IR analysis (ATR, solid-state) was carried out without further purification. The IR spectrum of this crude product was overlaid with that of the *trans*-$[PdCl_2(N\text{-morpholine})_2]$ complex **17** (Figure S14). The synthesis and characterization data for **17** can be found in Section 3.5.

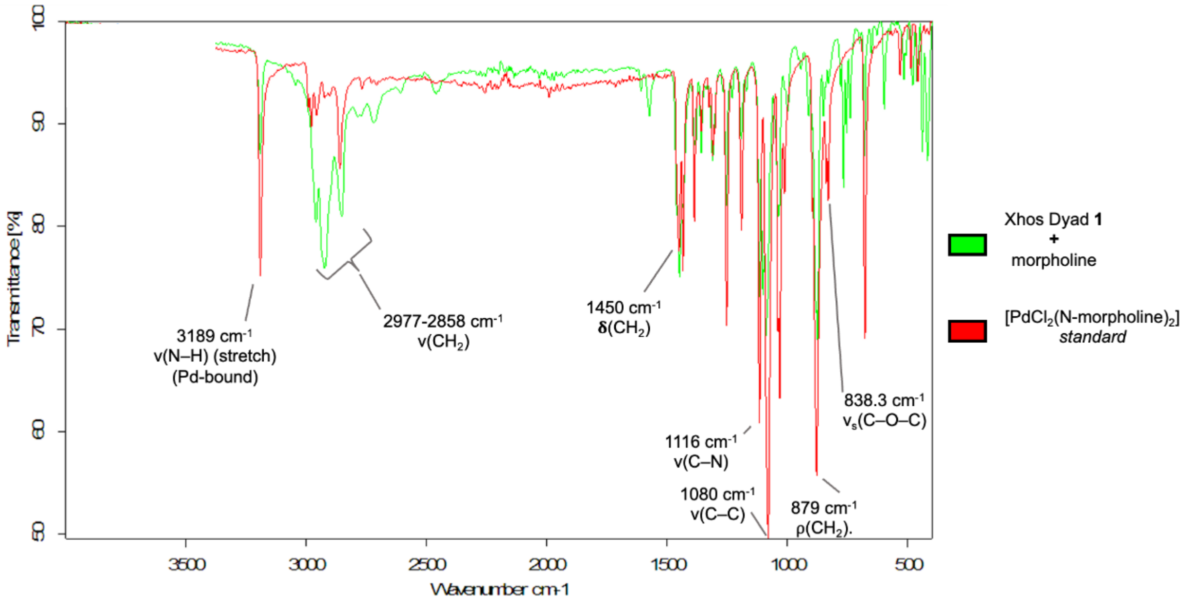

Figure S14: IR spectrum (ATR) of the product of the reaction of $[HXPhos]_2[Pd_2Cl_6]$ **1** with morpholine (green), overlaid with an authentic sample of *trans*-$[PdCl_2(morpholine)_2]$ (red). Key absorption bands have been assigned.

2.3.3. Reaction of $[HXPhos]_2[Pd_2Cl_6]$ 1 with NaO*t*Bu and morpholine and oxidative addition reactions therefrom (C, D; Figure 5, Main Paper)

Generation of $[Pd^0(XPhos)_2]$

A Schlenk tube charged with $[HXPhos]_2[Pd_2Cl_6]$ **1** (50 mg, 0.036 mmol) and NaO*t*Bu (10 equiv.) was evacuated and backfilled with N_2 (3 times). THF (2 mL dry, degassed) was added, followed, 1 minute later by morpholine (31 uL, 0.36 mmol; 10 equivalents), and the resulting mixture was magnetically stirred at room temperature for 20 minutes. After this time, a sample was taken for NMR analysis according to the procedure detailed in Section 2.1.1.

Oxidative Addition Reaction

To the above NMR sample, containing **2**, aryl halide (10 µL: *para*-fluorochlorobenzene **10c**, 12.26 mg, 0.094 mmol, ~ 8 equiv.; or *para*-fluoroiodobenzene **10a**, 19.3 mg, 0.086, ~ 7 equiv.) was added neat via a microsyringe. The sample was subsequently subject to NMR analysis: incomplete conversion of **2** to $[Pd^{II}Cl(p\text{-}C_6H_4\text{-}F)(XPhos)(N\text{-morpholine})]$ was evident on the reaction of *para*-fluorochlorobenzene **10c** after *ca.* 10 minutes. See main paper for stacked NMR spectra of the timecourse.

Additionally, LIFDI-MS data was obtained for the oxidative addition reaction product of $Pd^0(XPhos)_2$ and *para*-fluorochlorobenzene **10c** (Figure S15). Here two key ions have been identified, most likely representative showing evidence for oxidative addition, breaking the C–Cl bond, which was detected by NMR spectroscopic analysis, although no *N*-morpholino adducts were detected in this case, it is presumed that the morpolinyl ligand is labile under MS conditions, particularly considering the steric environment around the phosphine-bound Pd complex. Other identifiable ions in the spectrum below are $[XPhos\text{–}isopropyl]^{\cdot+}$ (m/z 433.30580; calc. for $C_{30}H_{42}P$ m/z 433.30241; common fragment), $[XPhos{=}O]^{\cdot+}$(m/z 492.35179; calc. for $C_{33}H_{49}OP$, m/z 492.35210), and $[Pd(XPhos)]^+$(m/z (m/z 582.26303; calc. for $C_{33}H_{49}PPd$, m/z 582.26067).

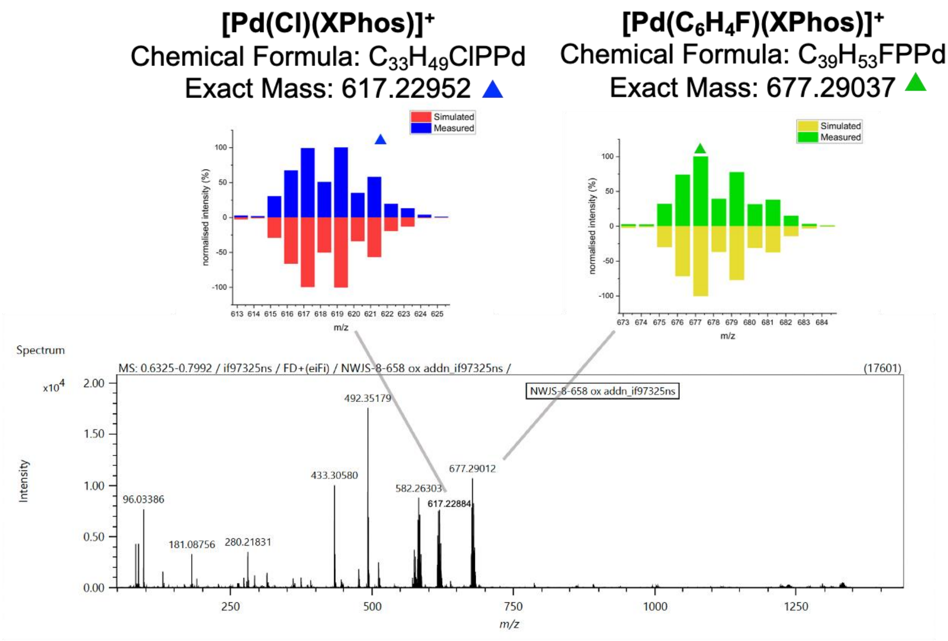

Figure S15: LIFDI MS Spectrum with expansions featuring simulated-measured isotopic distribution comparisons for key ions representative of oxidative addition products of $[Pd^0(XPhos)_2]$ and *para*-fluorochlorobenzene **10c**.

2.3.4. Reaction of *trans*-$[PdCl_2$(*N*-morpholine)$_2]$ with NaO*t*Bu and XPhos

Trans-$[PdCl_2$(*N*-morpholine)$_2]$ **17** (25.3 mg, 0.036 mmol) was added to a Schlenk tube alongside NaO*t*Bu (34.6 mg, 0.72 mmol; 20 equiv.) and XPhos (34.3 mg, 0.072 mmol). The flask was evacuated and backfilled with N_2 (3 times), THF (2 mL dry, degassed) was added, and the resulting mixture was magnetically stirred at room temperature for 20 minutes. After this time, a sample was taken for NMR analysis (see Figure S16) according to the procedure detailed in Section 2.1.1.

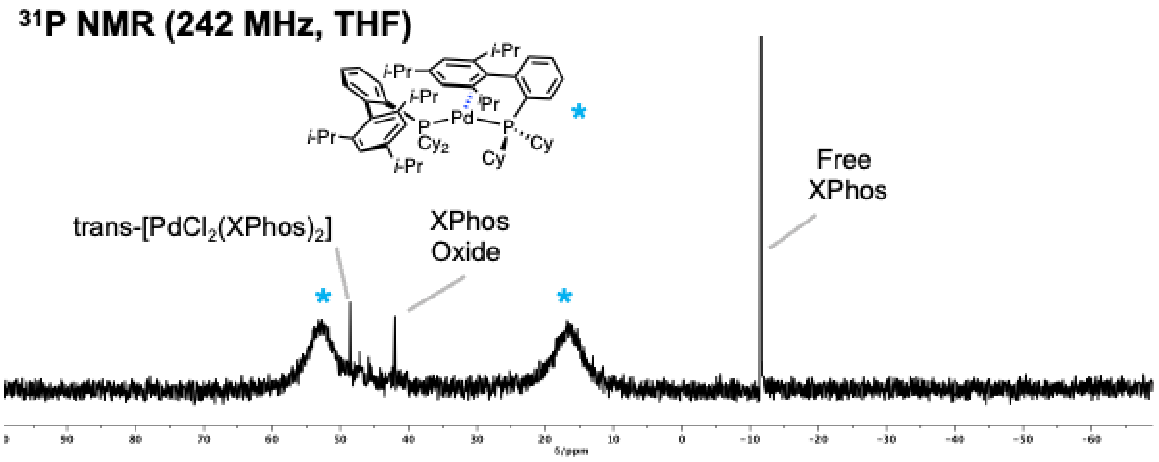

Figure S16: ^{31}P NMR spectrum, showing peaks characteristic of $[Pd^0(XPhos)_2]$ **9**, generated from *trans*-$[PdCl_2$(N-morpholine)$_2]$ **17** in the presence of XPhos.

2.3.5. Reaction of authentic [Pd^{II}(Br)(*p*-C_6H_4F)(XPhos)] 11' with morpholine.

A 20 mL Schlenk flask equipped with a magnetic stirrer bar and a septum was charged with [PdBr(*p*-C_6H_4F)(XPhos)] **11'** (100 mg, 0.13 mmol, 1.0 equiv.)). The flask was deoxygenated *via* vacuum/N_2 cycles (3×1 min each). After, heptane (4 mL) and morpholine (115.8 mg, 115 µL, 1.33 mmol, 10 equiv.) The reaction mixture was stirred under N_2 for 3 h at 50 ˚C. After this time a clear solution with white precipitate was observed. The solid was isolated by vacuum filtration and washed with heptane (3×2 mL) to give 76 mg (65% yield) of a white powder.

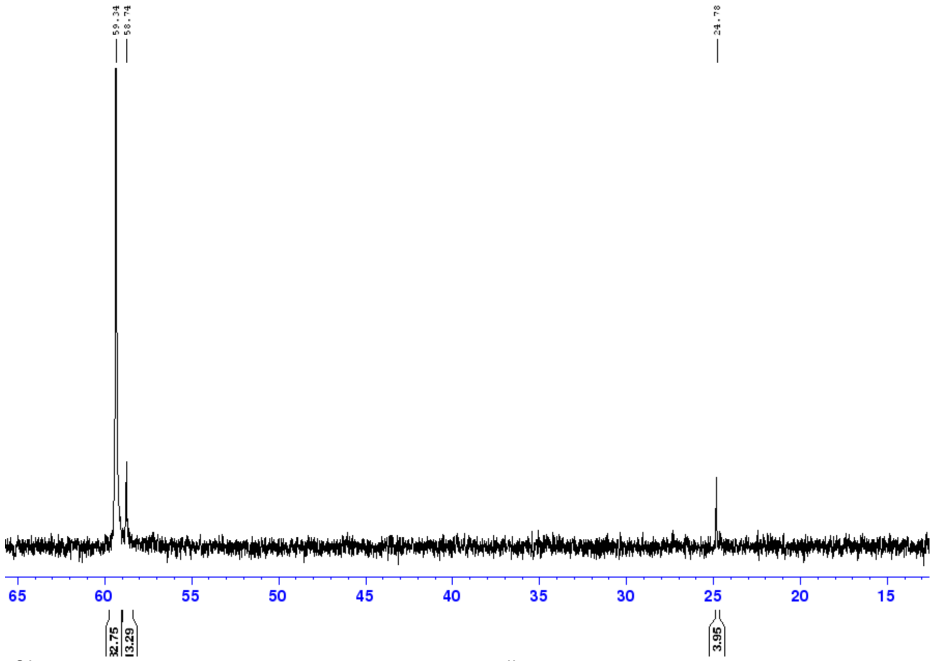

Figure S17: ^{31}P NMR spectrum of synthesized [Pd^{II}(Br)(*p*-C_6H_4–F)(*N*-morpholine)(XPhos)] **18b**.

2.3.6. Reaction of [HXPhos]$_2$[Pd$_2$Cl$_6$] 1 with NaO*t*Bu and *N*-methyl morpholine

A 20 mL Schlenk flask equipped with a stir bar and a septum was charged with XPhos Dyad Palladate (69 mg, 0.05 mmol, 1.0 equiv.) and NaO*t*Bu (40 mg, 0.5 mmol, 10 equiv.). The flask was deoxygenated *via* vacuum/N_2 cycles (3×1 min each). After, THF (2.8 mL; dry, degassed) was added to the flask *via* syringe. The reaction was left to stir for 1 minute before adding *N*-methyl morpholine (51 mg, 51 µL, 0.5 mmol, 10 equiv.). The reaction mixture was left to stir for 1h at 22 ˚C. A Wilmad® screw-cap NMR tube equipped with a polypropylene cap with PTFE-faced silicone was deoxygenated by sparging with N_2 for *ca.* 30 minutes. A sample of the reaction mixture was charged into the degassed NMR tube *via* syringe and the sample was analyzed by ^{31}P NMR spectroscopic analysis without further purification.

2.3.1. Reaction of $[HXPhos]_2[Pd_2Cl_6]$ 1 with NaO*t*Bu and aniline

A Schlenk tube charged with $[HXPhos]_2[Pd_2Cl_6]$ **1** (50 mg, 0.036 mmol) and NaO*t*Bu (10 equiv.) was evacuated and backfilled with N_2 (3 times). THF (2 mL dry, degassed) was added, followed, 1 minute later by aniline (33 μL, 0.36 mmol; 10 equivalents), and the resulting mixture was magnetically stirred at room temperature for 20 minutes. The same reaction was carried out at 60 °C, and in presence of additive H_2O (10 μL, degassed) at room temperature. In all cases no Pd^0 complex was detected by ^{31}P NMR spectroscopic analysis.

2.4. Buchwald–Hartwig Catalytic Activity Experiments

2.4.1. General procedure for Buchwald–Hartwig test reactions

2.4.1.1. Test reactions involving 6-chloroquinoline (Schlenk conditions)

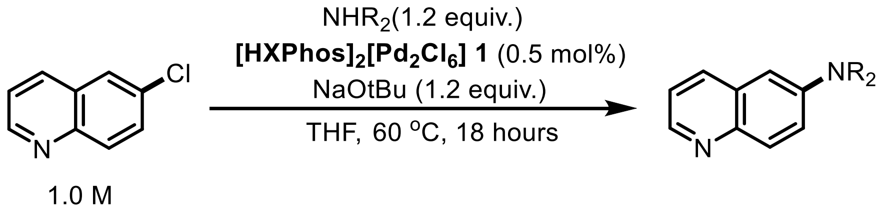

An oven-dried Schlenk tube was charged with 6-chloroquinoline (327.2 mg, 2.0 mmol, 1 equiv.), NaO*t*Bu (230.6 mg, 2.4 mmol, 1.2 equiv.), $[HXPhos]_2[Pd_2Cl_6]$ **1** (13.81, 0.01 mmol, 0.5 mol%) and 1,3,5-trimethoxybenzene (112.2 mg, 0.667 mmol, 0.333 equiv.; internal standard). The reaction was sealed in a Schlenk tube (SubaSeal®) and evacuated and backfilled with N_2 (3 times) before THF (2 mL; dry, degassed) was added via a syringe over the septum. The resulting mixture was magnetically stirred for 1 minute at 60 °C. before the amine (2.4 mmol, 1.2 equiv, see Table S5) was rapidly added via a syringe (care taken make sure all drops drop directly into the stirred reaction mixture) over the septum and the resulting mixture was stirred at this temperature for the specified reaction time. See Section 3.3 for characterization data for the 4-(quinolin-6-yl) morpholine product.

2.4.1.2. Reactions involving *para*-fluorochlorobenzene as substrate

An oven-dried Schlenk tube was charged with NaO*t*Bu (230.6 mg, 2.4 mmol, 1.2 equiv.), $[HXPhos]_2[Pd_2Cl_6]$ **1** (13.81, 0.01 mmol, 0.5 mol%) and 1,3,5-trimethoxybenzene (112.2 mg, 0.667 mmol, 0.333 equiv.; internal standard). The reaction was sealed in a Schlenk tube (SubaSeal®) and evacuated and backfilled with N_2 (3 times) before THF (2 mL; dry, degassed) was rapidly added, followed by *para*-fluorochlorobenzene **12c** (261.1 mg, 212 μL, 2,0 mmol, 1.0 equiv.) *via* a syringe over the septum. The resulting mixture was magnetically stirred for 1

minute at 40 ^{o}C. before the amine (2.4 mmol, 1.2 equiv., see 5) was rapidly added *via* a syringe (care taken make sure all drops drop directly into the stirred reaction mixture) over the septum and the resulting mixture was stirred at this temperature for 18 hours. See section 3.2 for full Characterization data for the 4-(4-fluorophenyl)morpholine product.

Table S5: Showing quantities (mass and volume) of amine used in Buchwald–Hartwig amination test reactions alongside conversion results. Conversion was determined by ^{1}H NMR against a 1,3,5-trimethoxybenzene internal standard, according to the general procedure reported in 2.1.3

**Amine**	**Quantity**		**Conversion/%**
	**m/mg**	**V/µL**	
morpholine	209	206	95
aniline	223	216	0

2.4.2. General workup procedures: Buchwald–Hartwig amination reactions

After the specified reaction time, H_2O (5 mL, deionised) was added, and the organic layer was separated. The bottom layer was extracted with EtOAc (3×5 mL) and the combined organic layers were dried over $MgSO_4$ before being filtered over Celite™ (1 cm depth on a sinter funnel). This filtrate was concentrated *in vacuo*. The residue could be analyzed by ^{1}H NMR spectroscopy. The product could be purified by flash column chromatography on SiO_2. Reactions could additionally be monitored by sampling (100 µL,), quenching by dilution in NH_4Cl (1 mL; aq. sat.) and extracting on small scale with EtOAc (3×1 mL). The combined organic extracts were dried over $MgSO_4$ and filtered through Celite™ (1 cm depth) in a pipette supported with cotton wool. Crude mixtures could be purified – see sections 3.2 and 3.3 for chromatographic details.

2.4.3. Catalyst screening of Buchwald–Hartwig coupling reaction: XPhos-containing pre-catalyst *vs*. concentration

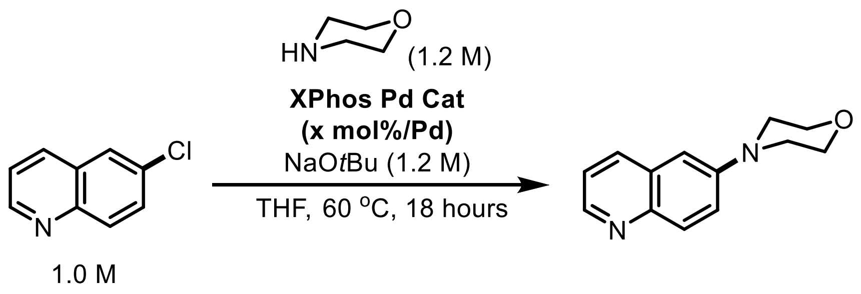

Internal standard: 1,3,5-trimethoxybenzene x= 2.0, 1.0, 0.5, 0.25, 0.125

XPhos Pd Cat = $[HXPhos]_2[Pd_2Cl_6]$, XPhos-Pd-Gen3, XPhos Crotyl

Figure S18: General Reaction for Pd-XPhos-catalysed Buchwald-Hartwig reactions.

2.4.3.1. Process Overview

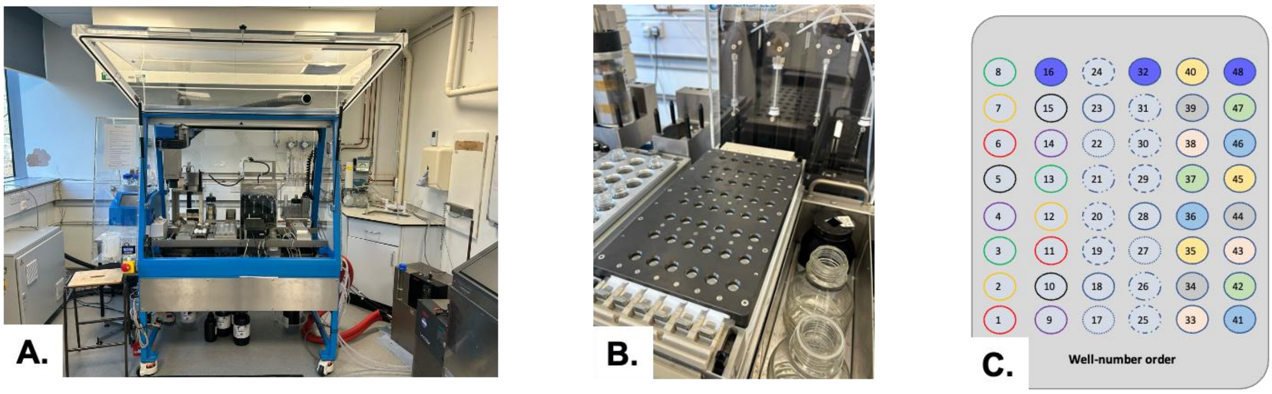

Figure S19: The Chemspeed® Robotic Platform: A. The entire platform; B. The ISYNTH reactor, showing the 48 wells; C. A schematic showing a birds eye view of the reaction wells of the ISYNTH reactor.

A medium-throughput screening of 48 parallel reaction-setups was performed using an ISYNTH reactor (part of a Chemspeed® robotic platform (Figure S19)). Reaction preparation was automated using the Chemspeed® robotic platform with each reaction analyzed using at-line HPLC and off-line LCMS analyses. 48 reaction vials were prepared, featuring the following: 0.5 mmol of 6-chloroquinoline (limiting reagent) in 0.5 mL of THF solvent (1.0 M, with respect to 60 °C 6-chlroroquinoline starting material). The reaction was setup as follows:

- 3× catalysts ($[HXPhos]_2[Pd_2Cl_6]$ **1**, XPhos Buchwald Gen3 & XPhos Crotyl).
- 3× different catalyst loadings with each catalyst (0.125, 0.25, 0.5, 1, 2 mol%).
- Each was performed in triplicate.

=> **Subtotal = 45 reactions**

- + 3 × control reactions with no added catalyst - no conversion seen.

⇨ **total of <u>48 reactions</u>**

2.4.3.2. Operations

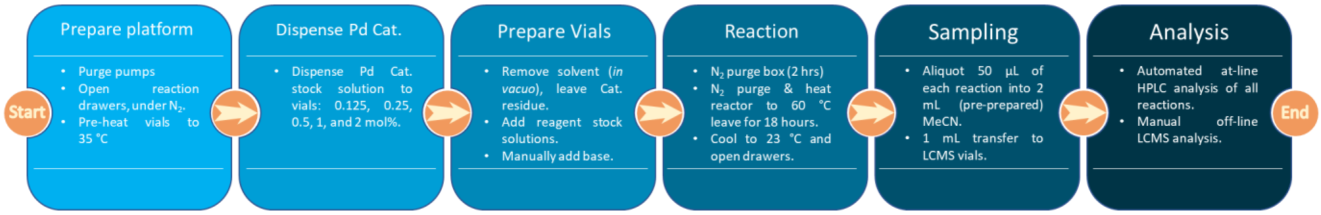

Figure S20: Flow chart summary for automated reaction screening of a palladium catalysed Buchwald Hartwig amination.

Unless otherwise stated all operations were performed by the Chemspeed® robotic platform in an automated, or semi-automated fashion.

A. Catalyst Preparation

1) Initial CH_2Cl_2 catalyst stock solutions were made up manually (See Section 2.4.3.4) and diluted in series by the Chemspeed® robotic platform, according to the following protocol:
    a. Take 500 µL of stock solution (2 mol% catalyst solution)
    b. Take 250 µL and add to 250 µL of CH_2Cl_2 (1 mol% solution)
    c. Take 250 µL and add to 250 µL of CH_2Cl_2 (0.5 mol% solution)
    d. Take 250 µL and add to 250 µL of CH_2Cl_2 (0.25 mol% solution)
    e. Take 250 µL and add to 250 µL of CH_2Cl_2 (0.125 mol% solution)
    f. Take 250 µL discarded (waste)

   This serial dilution process was run in triplicate for each catalyst.
2) All vials (8 mL total volume, diameter = 17 mm, height = 67 mm) were sealed in the ISYNTH (inbuilt PTFE cone seals), and all of the solvent (CH_2Cl_2) evaporated *in vacuo* at 35 °C, leaving the catalyst as a residue in each c.

B. Base/Substrates/Internal Standard Addition

3) All vials were removed from Chemspeed® robotic platform, sealed (using screw-cap lids and PTFE/silicone septa) and NaO*t*Bu (base) was added manually (as solid, pre-weighed, see Table S6 for quantities).
4) Once added, all vials were returned to the ISYNTH reactor, in the Chemspeed® robotic platform, and each vial was sealed and purged with an N_2 atmosphere.
5) 500 µL of substrate/internal standard stock solution - containing substrates 6-chloroquinoline, morpholine, and 1,3,5-trimethoxybenzene (internal standard) - was transferred into each vial (see Table S6 for quantities) on top of the catalyst residue in all 48 reaction vials. The atmosphere of the entire robotic platform was purged with N_2 for 1 hour.

Continued.

C. *Reaction*

6) The ISYNTH was heated to 60 ^{o}C (heater oil temperature set to 70 °C) under an atmosphere of N_2 with vortex mixing. Once the ISYNTH and contained vials were at thermal equilibrium (*ca.* 40 minutes)
7) The reaction was shaken vigorously (600 rpm) at 60 ^{o}C for 18 hours.
8) The ISYNTH was cooled to 23 ^{o}C before reactions were sampled.

D. *Sampling for HPLC analysis*

9) Aliquots of each reaction mixture (50 µL) were taken (whilst gentle mixing), and individually transferred into an 8 mL sample vial containing MeCN (3 mL).
10) 1 mL of diluted sample was into transferred in int 1.5 mL septum-sealed sample vials for HPLC analysis.
11) The HPLC column used for separation was an Ascentis® Express C18 (53826-U), 5 cm x 4.6 mm, 2.7 µm.

2.4.3.3. Quantities of substrates, internal standard and base used

A stock solution was made up manually in 60 mL glass screw-capped vial fitted with a PTFE septum. 6-chloroquinoline and 1,3,5-trimethoxybenzene (1,3,5-TMOB) were weighed in to the vial. The vial was sealed before being evacuated and backfilled with N_2 from a Schlenk line. Morpholine (previously degassed) was added via syringe over the sealed septum under N_2 flow. (See Table S7 for details on quantities). THF (27.5 mL; dry, degassed) was then syringed into the vial under flow of N_2, and the vial was sealed and introduced to the Chemspeed® robotic platform for aliquoting.

NaO*t*Bu (base) was weighed in individually and as a solid to the vials (See above: section 2.4.3.2) (machine-dried THF; degassed).

Table S6: Quantities of substates, base and internal standard. Mass required for the 48 reactions in 24 mL of stock solution (0.5 mL per reaction); [b] Total mass weighed in to make up the total stock solution (27.5 mL), allowing for excess volume (practical reasons – 'dead' volume needed for aliquoting); [c] manually weighed into the vials.

Compound	m/mg	M_w/gmol^{-1}	n/mmol	Concentration / M	Total mass used: 24 mL[a] / mg	Total mass weighed :27.5 mL[b] stock solution / mg (Reactions + 'dead' volume)
6-chloroquinoline	81.8	163.60	0.5	1.0	3926	4499
morpholine	52.3	87.12	0.6	1.2	2510	2877
1,3,5-TMOB	28.0	168.19	0.167	0.333	864	1542
NaO*t*Bu[c]	57.7	96.1	0.6	1.2	(2767)	N/A

2.4.3.4. Catalyst Stock Solutions

To 8-mL vial screw-capped vial equipped with a PTFE septum, the pre-catalyst was weighed in (see Table S7 for quantities). The vial was evacuated and backfilled with N_2 three times on a Schlenk line adapted with a needle. The pre-catalyst was then dissolved in CH_2Cl_2 which was syringed in with a needle over the septum under a flow of N_2.

Table S7: Precatalyst quantities used to make up stock solutions for automated dispensing by the Chemspeed Robotic system.

		Quantity per reaction (2 mol% loading)		Quantity used to make 3 mL stock solution		
**Pre-Catalyst**	**Mw /gmol^{-1}**	**m/mg**	**n/mmol**	**n/mmol**	**m/mg**	**Concentration of 3 mL stock solution /M**
**$[HXPhos]_2[Pd_2Cl_6]$**	1381.01	6.91	0.005	0.06[a]	**82.86**	0.02
**XPhos Gen3**	846.45	8.46	0.01	0.12	**101.57**	0.04
**XPhos Crotyl**	671.68	6.71	0.01	0.12	**80.60**	0.04

2.4.4.4. HPLC Analysis for Chemspeed Reaction Screening

Chromatographic Details: A Dionex Rapid Separation 3000 HPLC System was used, featuring a PDA-3000 photodiode array UV-Vis detector. An Ascentis® Express C18 (53826-U) (Reverse-phase), 5 cm x 4.6 mm, 2.7 µm. Chromatography was run at 25 ^{o}C. Products were identified by running isolated standards against the crude material, as well as identification by tandem MS. MeCN in H_2O mobile phase. Constant flow rate 400 µL/min, gradient of 40-90% MeCN in H_2O gradient over 4 minutes, 2 minute hold at 90% MeCN. 90-40% MeCN gradient, 30 seconds to return to 40%. 1 minute hold at 40% MeCN.

Reaction conversions were determined using HPLC analysis against the 1,3,5-trimethoxybenzene internal standard. Single absorbance wavelength of 254 nm used for starting material (S.M.), product and internal standard detection and quantification.

Starting material and product calibration for reaction quantification was carried out by preparing a stock solution containing both 6-chloroquinoline (Starting material) and 6-(N-morpholine)-quinoline, performing serial dilutions to obtain a range of differing concentrations, using a 0.333 M 1,3,5-trimethoxybenzene MeCN solution as the diluent, assuring the same concentration of the internal standard as the reaction aliquots. The analyte (S.M. or P) response peak integral was divided by the IS peak integral, to obtain the response factor which was graphed against concentrations. Each data set was subject to linear fits and straight-line equations were obtained for each analyte (Graph 1). Dilutions were considered to arrive at the final concentration.

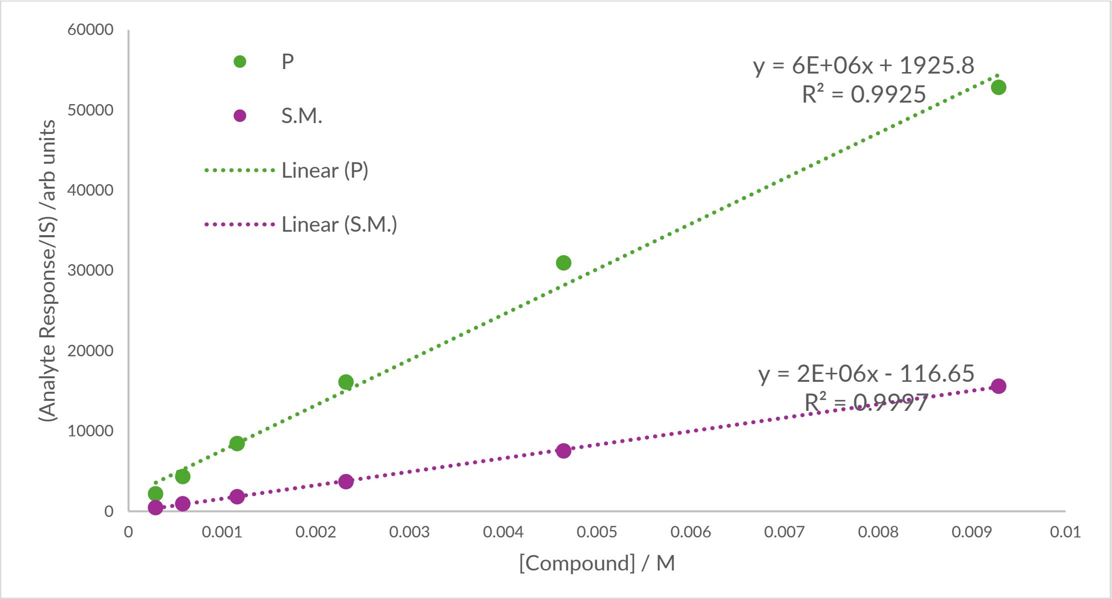

Graph 1: Calibration Curve for quantification of 6-chloroquinoline (S.M.) and 6-(morpholino)-quinoline (P).

3. Compound Characterization Data

3.1. 4-Fluoro-1,1'-biphenyl (4) [11]

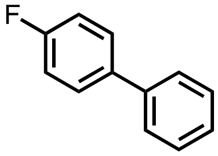

The compound, which appeared as a white powder a synthesised according to procedures reported in sections (2.1.2 and 2.1.2.2).

^{1}H NMR (400 MHz, Chloroform-*d*) δ 7.61 – 7.50 (m, 4H), 7.49 – 7.40 (m, 2H), 7.40 – 7.30 (m, 1H), 7.17 – 7.09 (m, 2H).
^{13}C NMR (101 MHz, Chloroform-*d*) δ 162.61 (d, $J_{C–F}$ = 246.1 Hz), 140.41, 137.48 (d, $J_{C–F}$ = 3.4 Hz), 128.97, 128.83 (d, $J_{C–F}$= 8.1 Hz), 127.29 (d, $J_{C–F}$ = 23.6 Hz), 115.76 (d, $J_{C–F}$= 21.3 Hz).
^{19}F NMR (376 MHz, Chloroform-*d*) δ -115.76 (m).
Infrared Spectroscopy (v_{max} /cm^{-1}): 3060 (w), 3039 (w), 2923 (w), 2853 (w), 1596 (m), 1572(s), 1518 (s), 1481 (s), 1452 (m), 1233 (s), 1193 (s), 1162 (s), 1105 (m), 1105 (m).
HRMS-(GC-)EI m/z = 172.06828 [M·]$^{+}$: $C_{12}H_{9}F$ requires 172.06861; error (pm =1.91 ppm).
R_f (hexane, neat) = 0.32.

3.2. 4-(4-Fluorophenyl)morpholine (14) [12,13]

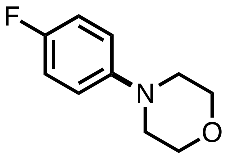

The title compound appeared as a light-yellow oil, isolated in 75% yield, according to the procedure detailed in Section 2.1.1.

^{1}H NMR (400 MHz, Chloroform-*d*) δ 7.03 – 6.92 (m, 2H), 6.93 – 6.82 (m, 2H), 3.90 – 3.81 (m, 4H), 3.13 – 3.00 (m, 4H).
^{13}C NMR (101 MHz, Chloroform-*d*) δ 157.45 (d, $J_{C–F}$ = 239.2 Hz), 148.06 (d, $J_{C–F}$ = 2.3 Hz), 117.61 (d, $J_{C–F}$ = 7.7 Hz), 115.75 (d, $J_{C–F}$ = 22.1 Hz), 67.06, 50.46.
^{19}F NMR (376 MHz, Chloroform-*d*) δ -124.11 (tt, *J* = 8.6, 4.5 Hz).
HRMS ESI-MS m/z = 182.0976 [M+H]+: $C_{10}H_{13}FNO$ requires; 182.0976 error (ppm) = -0.1.

Infrared Spectroscopy (v_{max} /cm^{-1}): 2061(w), 2854 (w), 2821 (w), 1507 (s), 1449 (m), 1378 (m), 1332 (w), 1330 (w), 1259 (m), 1229 (s), 1163 (m), 1118 (s), 927 (s), 858 (s), 815 (s), 713 (m), 527 (m), 496 (m).

R_f (SiO_2; EtOAc/hexane; 85:15) = 0.3. (Conditions used for purification by flash column chromatography).

3.3. 4-(Quinolin-6-yl) morpholine (16) [14]

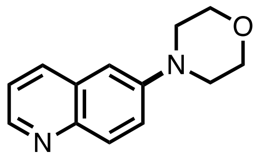

This compound was synthesised and isolated according to general procedure detailed in procedure Section 2.1.1.

^{1}H NMR (600 MHz, Chloroform-d) δ 8.72 (dd, J = 4.2, 1.7 Hz, 1H), 8.32 – 7.80 (m, 2H), 7.48 (dd, J = 9.3, 2.8 Hz, 1H), 7.32 (dd, J = 8.3, 4.2 Hz, 1H), 7.02 (d, J = 2.7 Hz, 1H), 3.98 – 3.82 (m, 4H), 3.33 – 3.19 (m, 4H).

^{13}C NMR (151 MHz, Chloroform-d) δ 149.30, 147.84, 144.10, 134.69, 130.25, 129.37, 122.05, 121.45, 109.01, 66.85, 49.45.

HRMS ESI-MS m/z = 215.1182 [M+H]$^{+}$: $C_{13}H_{15}N_2O$ requires; 215.1184 error (ppm) = -1.3.

Infrared Spectroscopy (v_{max} /cm^{-1}): 2954.5 (m), 2893.4 (m), 2865.1 (m), 1615.6 (m), 1585.8 (m), 1499.3 (m), 1455.2 (m), 1366.6 (m), 1261.7 (s), 1112.5 (vs), 1031.1 (m),959.6 (m), 930.8 (s), 871.9 (s), 826.3 (vs), 798.8 (m).

R_f (SiO_2; EtOAc/hexane; 80:20) = 0.21. (Conditions used for purification by flash column chromatography).

3.4. *trans*-bis(2-Dicyclohexylphosphino-2′,4′,6′-triisopropylbiphenyl) palladium dichloride; trans-[$PdCl_2(XPhos)_2$] (8) [15]

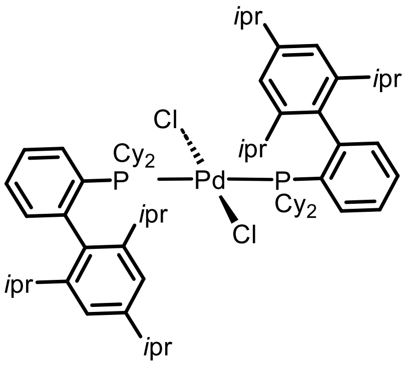

In a Schlenk flask $PdCl_2$ (100 mg, 0.564 mmol) was suspended in dimethyl sulfoxide (10 mL; anhydrous) with magnetic stirring. The liquid was freeze-pump-thaw-degassed using the Schlenk vacuum, refilling with N_2 after each pump-cycle. The mixture was then heated until the $PdCl_2$ had dissolved into a clear solution (*ca*. 135 °C; minimizing time heating at this maximum temperature). 2-Dicyclohexylphosphino-2′,4′,6′-triisopropylbiphenyl (XPhos; 538 mg, 1.128 mmol, 2 equivalents) was added and the reaction mixture immediately turned from a reddish-orange solution to a bright yellow stirred suspension. The flask was cooled to 90 °C and magnetically stirred for 1 hour after which time the reaction mixture was cooled to room temperature before being washed with dimethyl sulfoxide (2 × 2 mL) and diethyl ether (3 × 4 mL), the product, which appeared as a bright yellow powder was dried in vacuum (400 mg, 0.353; 63% yield).

^1H NMR* (600 MHz, Chloroform-d) δ 8.25 (br s, 2H), 7.37 (t, J = 7.6 Hz, 2H), 7.30 (t, J = 7.4 Hz, 1H), 7.01 (s, 2H), 6.99 (dd, J = 7.7, 1.7 Hz, 1H), 3.17–0.23 (m, 60H; very broad signals) 2.91 (hept, J = 6.9 Hz, 1H), 1.55 (s, 5H), 1.28 (d, J = 6.9 Hz, 5H), 1.20 (d, J = 6.5 Hz, 5H), 1.09 (s, 24H), 0.88 (t, J = 7.0 Hz, 0H), 0.81 (s, 5H). Broadness was evident for certain alkyl signals of trans-PdCl2(XPhos)2 at 25 °C indicating exchange processes in the ^1H and ^{13}C NMR timescale. This has been indicated in prior-published data peaks that were resolvable under the conditions have been quoted.[15a]

^{31}P NMR (243 MHz, Chloroform-d) δ 45.53.

Infrared Spectroscopy (vmax /cm^{-1}): 2960.4 (m), 2923.4 (m), 2848.8 (m), 1605.8 (w), 1463.0 (br, m), 1360.3 (m), 1320.8 (m), 1300.9 (m), 1196.9 (m), 1179.9 (m), 1123.4 (m), 1004.11., 872.7 (m), 849.0.

LIFDI-MS (+ve mod) m/z = 1128.55787 [M·]+: $C_{66}H_{98}Cl_2P_2Pd$ requires; 1128.55556 error (ppm) = 2.53.

* With this compound, the broadness of the ^1H NMR spectrum, particularly in the aliphatic-region, made accurate integration/separation of environments challenging. The mismatches

in ^{1}H NMR integration values are likely related due to this. A low temperature experiment was precluded because of the low solubility of **8**.

XRD Data: Single crystals (pale-yellow) could be grown by vapour diffusion of pentane onto a benzene solution of the reaction product.

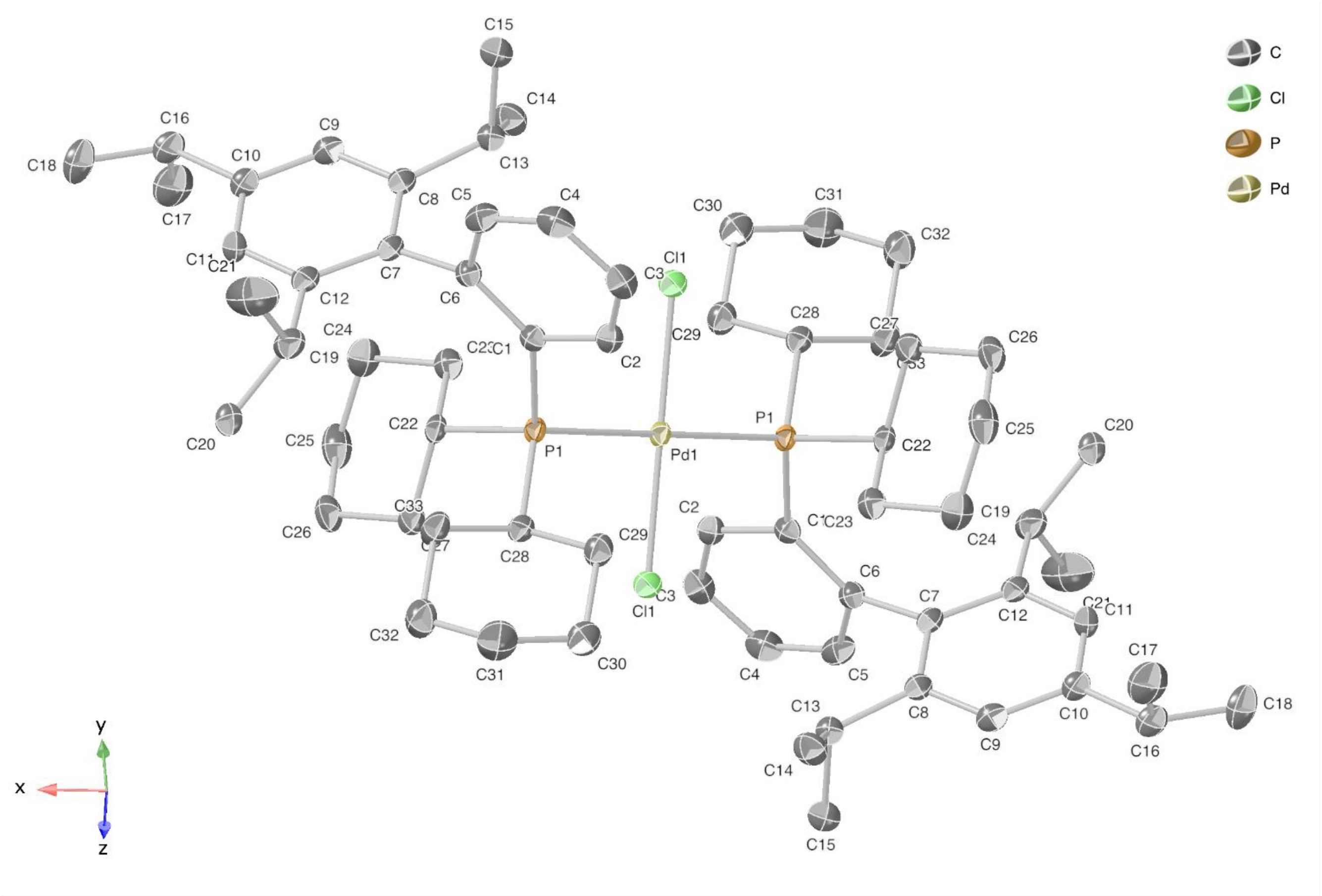

Figure S21: Single crystal X-ray diffraction structure of bis(2-dicyclohexylphosphino-2′,4′,6′-triisopropylbiphenyl) palladium(II) dichloride (**8**). Selected bond distances/Å: Pd1–P1 2.343, Pd1–Cl1 2.300, P1–C1 (sp^2) 1.836, P1–C28(sp^3) 1.856, P1–C22(sp^3) 1.848; Selected bond angles /^{o} P1–Pd–Cl 90.726, 89.274.

Note that the single crystal XRD structure of **8** has been reported elsewhere.[15b] The dataset described here is of higher quality than that reported[15b] (R2, R1, wR2, wR1, Goof 0.0780: 0.0566, 0.1643, 0.1508, 1.197, respectively).

Table S8: Key crystallographic data and structure refinement for ijsf21060.

CCDC number	2394940
Identification code	ijsf21060
Empirical formula	$C_{66}H_{98}Cl_2P_2Pd$
Formula weight	1130.68
Temperature/K	110.05(10)
Crystal system	monoclinic
Space group	$P2_1/c$
a/Å	15.53050(16)
b/Å	18.88103(19)
c/Å	10.35060(9)
α/°	90
β/°	92.8967(9)
γ/°	90
Volume/Å^3	3031.25(5)
Z	2
ρ_{calc}g/cm^3	1.239
μ/mm^{-1}	4.054
F(000)	1208.0
Crystal size/mm^3	0.177 × 0.082 × 0.046
Radiation	Cu Kα (λ = 1.54184)
2Θ range for data collection/°	7.376 to 134.134
Index ranges	-18 ≤ h ≤ 18, -22 ≤ k ≤ 22, -8 ≤ l ≤ 12
Reflections collected	21821
Independent reflections	5416 [R_{int} = 0.0262, R_{sigma} = 0.0238]
Data/restraints/parameters	5416/0/518
Goodness-of-fit on F^2	1.054
Final R indexes [I>=2σ (I)]	R_1 = 0.0252, wR_2 = 0.0621
Final R indexes [all data]	R_1 = 0.0314, wR_2 = 0.0646
Largest diff. peak/hole / e Å^{-3}	0.62/-0.39

3.5. Bis(*N*-morpholine) palladium dichloride (17) [16-17]

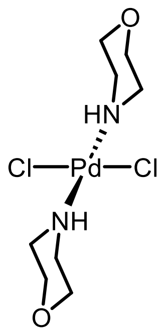

A Schlenk tube charged with $PdCl_2$ (200 mg, 1.12 mmol) was septum-sealed (SubaSeal) and brought under inert conditions by evacuating and backfilling with N_2. Acetonitrile (5.6 mL; dry, degassed) was added via syringe and the resulting mixture was placed under a cold finger and heated to reflux (82 ^oC) with magnetic stirring, yellow solid gradually precipitated from the red solution ([$PdCl_2(NCMe)_2$] intermediate). Once the brown solid, indicative of undissolved $PdCl_2$ had been solubilised, morpholine (196 uL, 2.26 mmol) was added over a septum and the mixture immediately changed from an orange solution to a stirred suspension featuring a yellow powder (solid immediately crashing out). The solvent was removed in vacuum and the filter was washed with Et_2O (3×5 mL). The pale-yellow powder residue was dried in vaccum (353 mg; 1.12 mmol; quant. yield). Extremely low organic-solvent solubility precluded solution-based handling and characterization (*e.g.* by NMR).

Infrared Spectroscopy (v_{max} /cm^{-1}): 3188.9 (s, typical of M–N coordinated N–H stretches[18,19]), 2976.6 (w, CH_2), 2956.1 (w, v(CH_2)), 2858.3 (w, v(CH_2)), 1449.6 (m, **δ**(CH_2)), 1443.6 (m **δ**(CH_2)), 1386.7 (m), 1358.0 (w), 1252.3 (m), 1190.8 (m), 1116.0 (s), 1079.5 (s), 878.5 (vs, ρ(CH_2)). 838.3 (w, v_s(C–O–C)).

C, H, N elemental analysis: Average of two runs: %C = 27.11, %H = 4.79, %N = 7.90, % remainder = 60.206; Theoretically for $C_8H_{18}Cl_2N_2O_2Pd$: %C = 27.33, %H = 5.16, %N = 7.97, % remainder = 61.54.

Characterization data matched that previously published in the literature.[16,17]

4. NMR Spectra

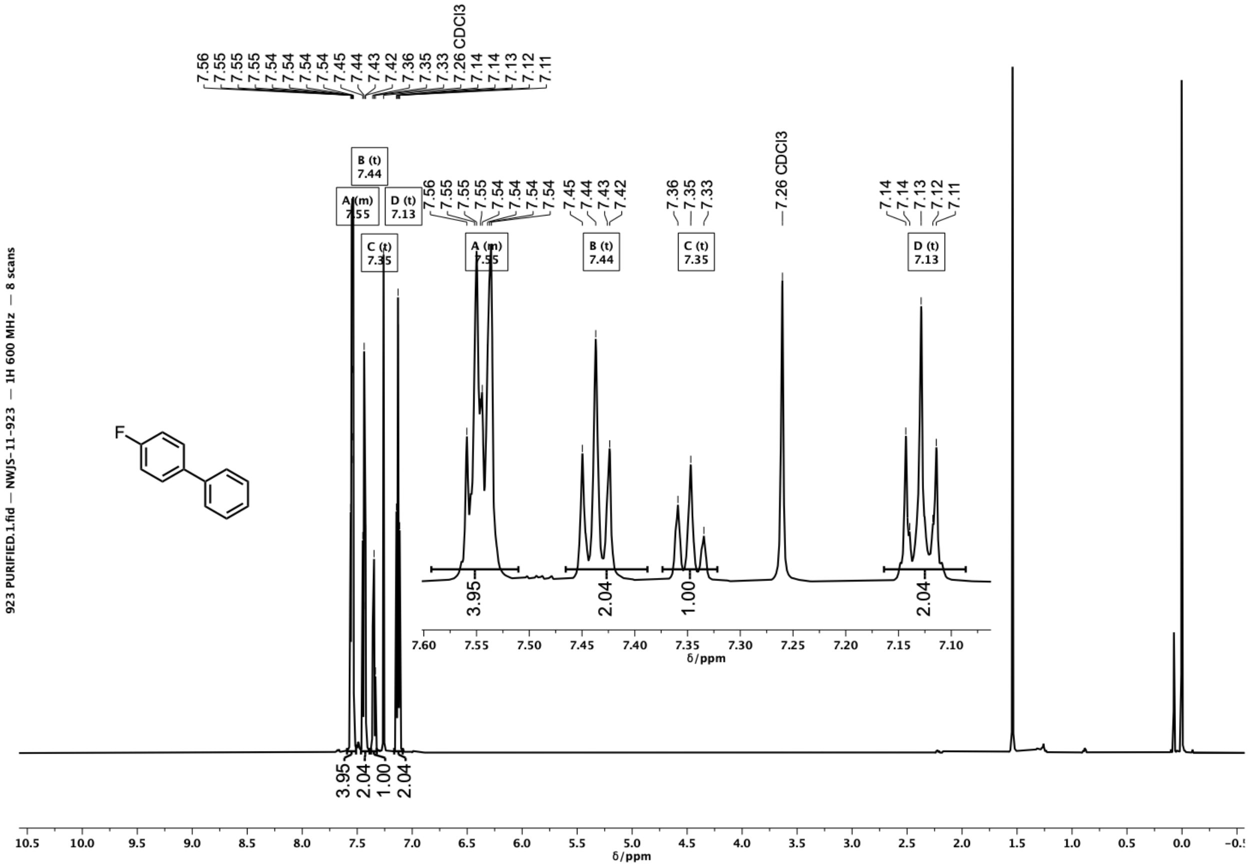

Figure S22: ^{1}H NMR ($CDCl_3$, 600 MHz, 8 scans) spectrum of 4-fluoro-1,1'-biphenyl (**4**).

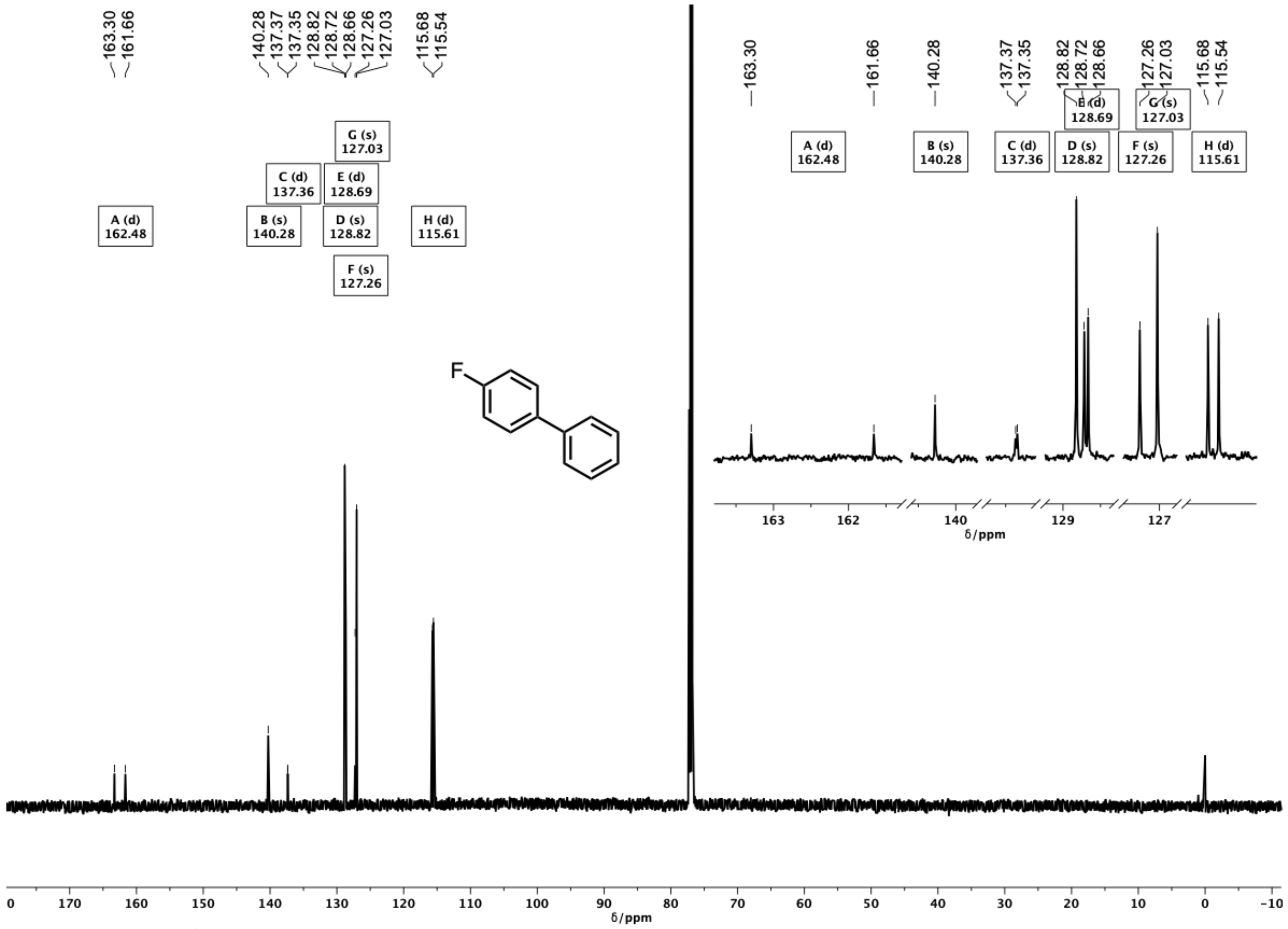

Figure S23: ^{13}C NMR ($CDCl_3$, 151 MHz, 1024 scans) spectrum of 4-fluoro-1,1'-biphenyl (**4**).

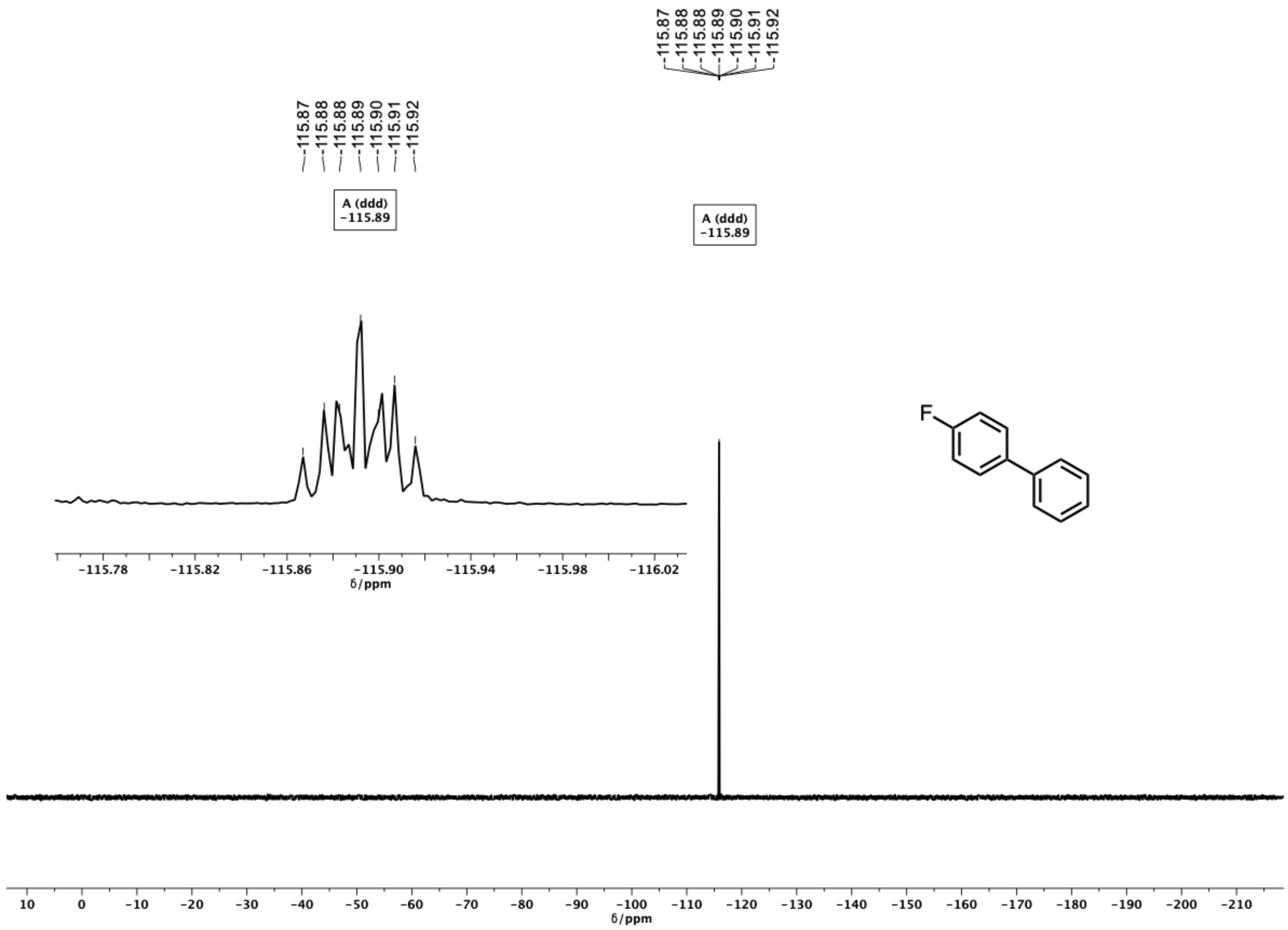

Figure S24: ^{19}F NMR ($CDCl_3$, 565 MHz, 8 scans) of 4-fluoro-1,1'-biphenyl (**4**).

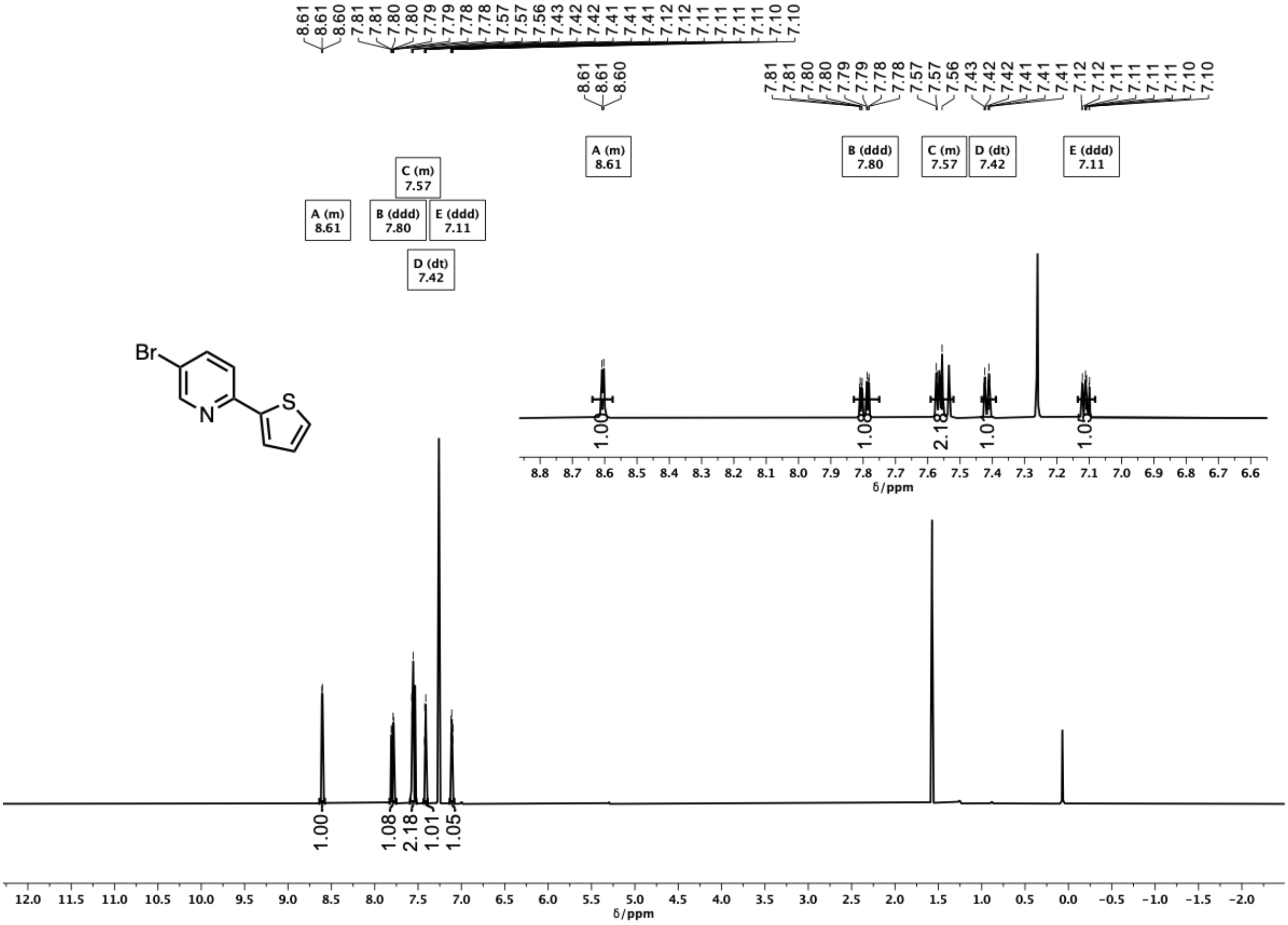

Figure S25: ^{1}H NMR ($CDCl_3$, 600 MHz, 8 scans) spectrum of 5-bromo-2-(2-thiophenyl) pyridine (**7b**).

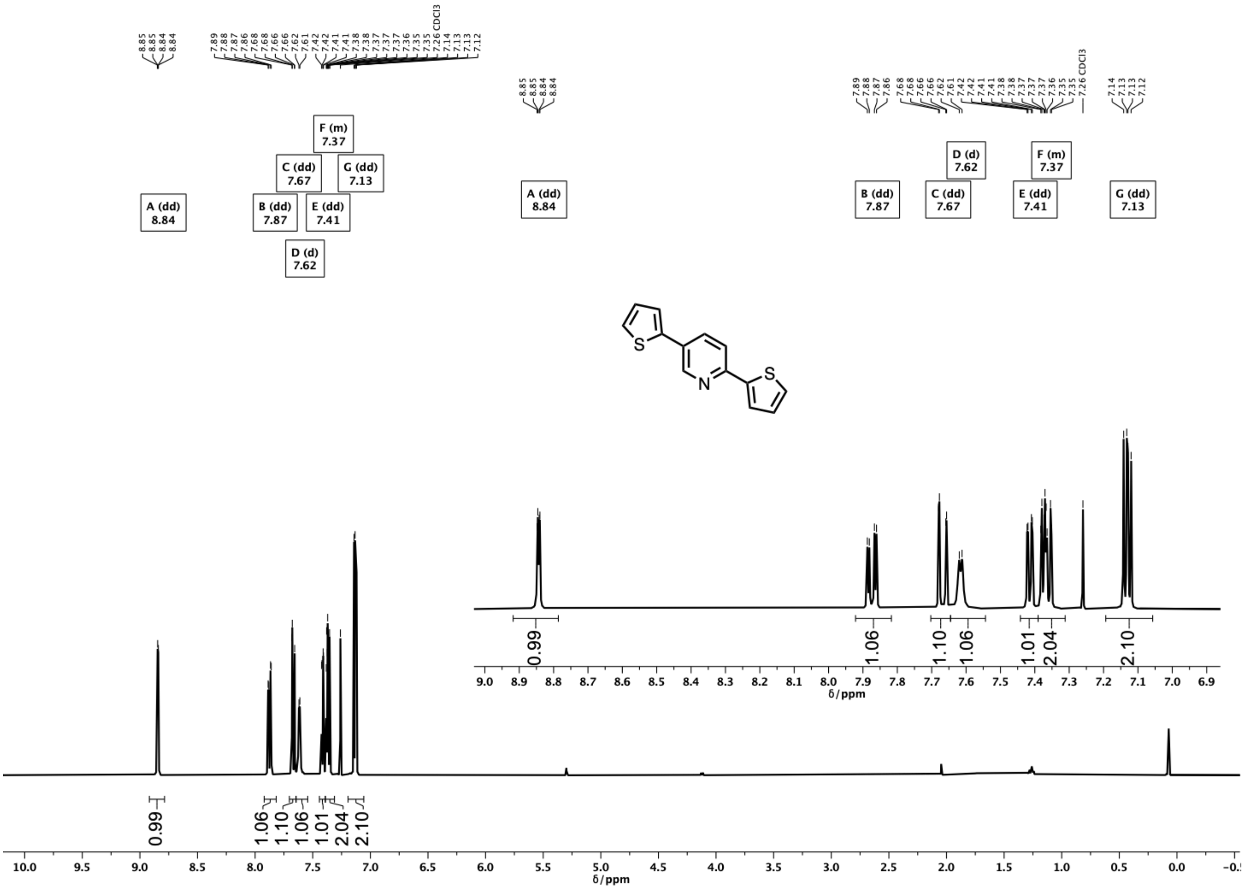

Figure S26: ^{1}H NMR ($CDCl_3$, 600 MHz, 8 scans) spectrum of bis-2,5-(2-thiophenyl) pyridine (**7c**).

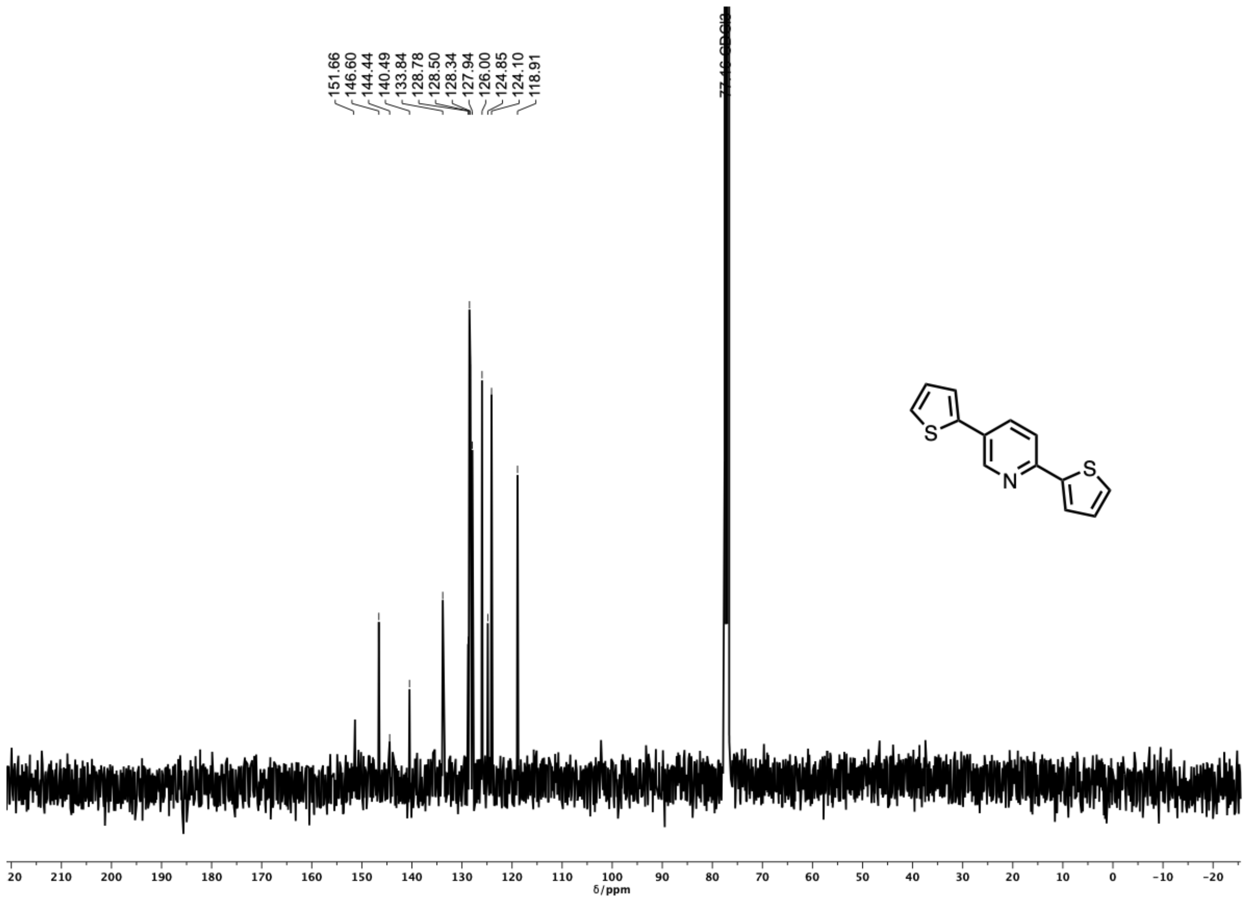

Figure S27: ^{13}C NMR spectrum ($CDCl_3$, 101 MHz, 1024 scans) of 5-bromo-2-(2-thiophenyl) pyridine (**7c**).

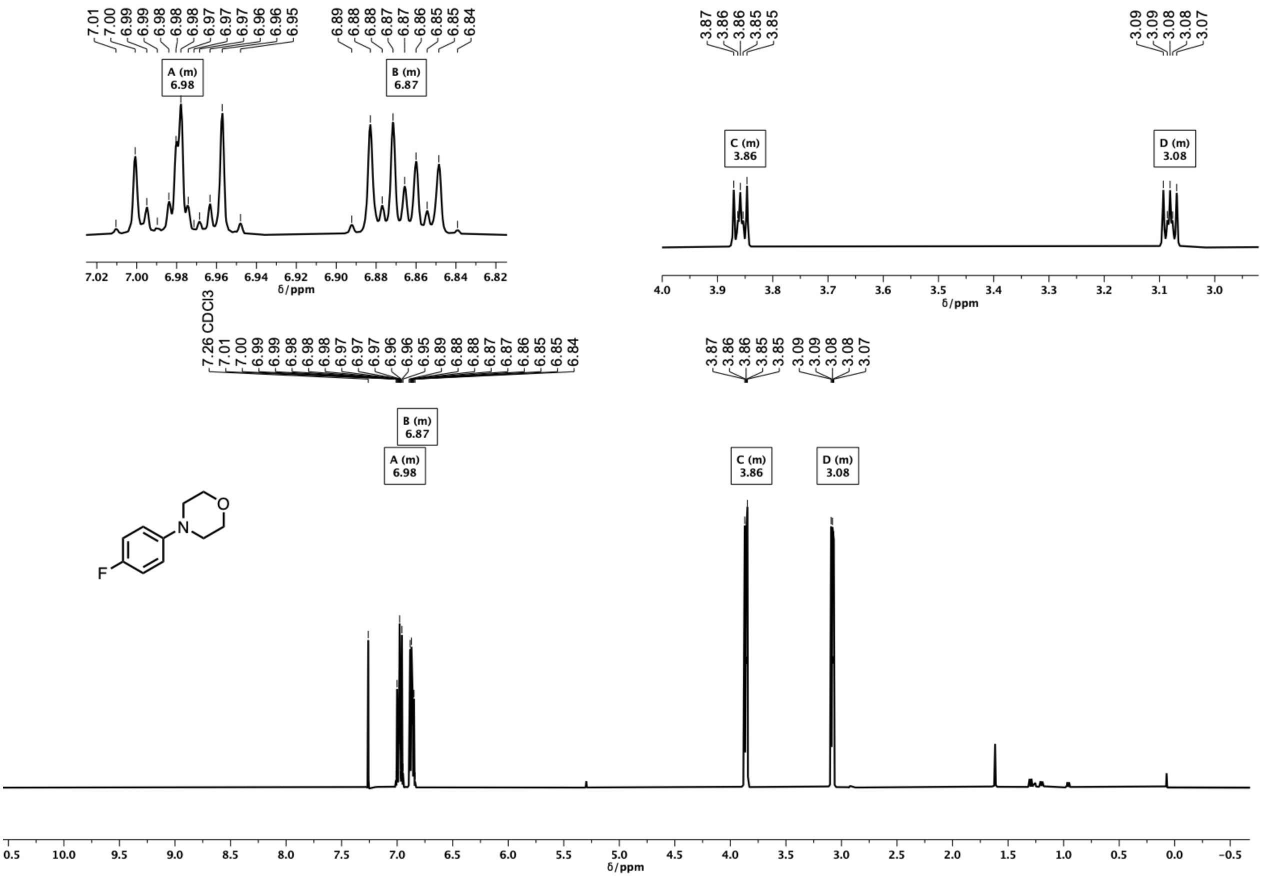

Figure S28: ^{1}H NMR spectrum ($CDCl_3$, 400 MHz, 8 scans) of 4-(4-fluorophenyl) morpholine (**14**).

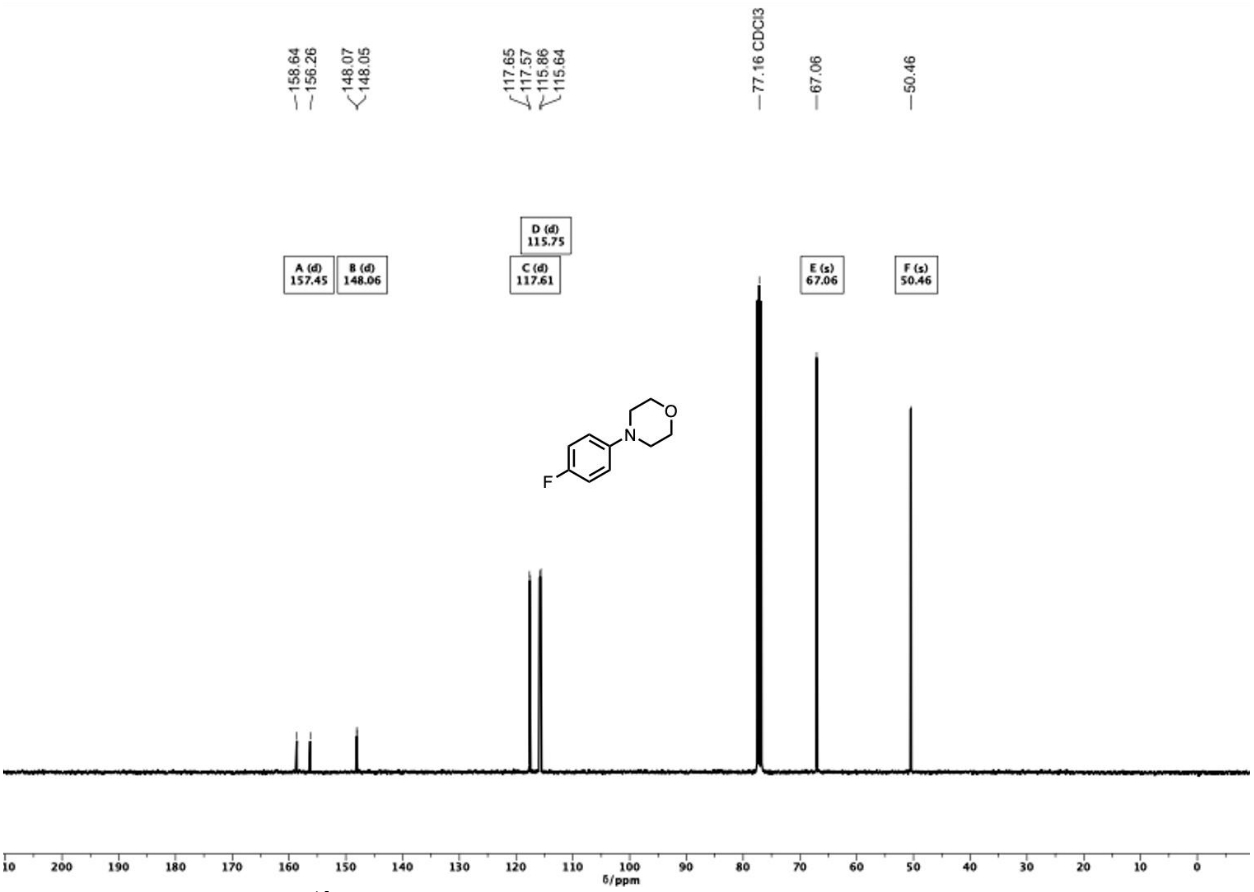

Figure S29: ^{13}C NMR spectrum ($CDCl_3$, 101 MHz, 1024 scans) of 4-(4-fluorophenyl)morpholine (**14**).

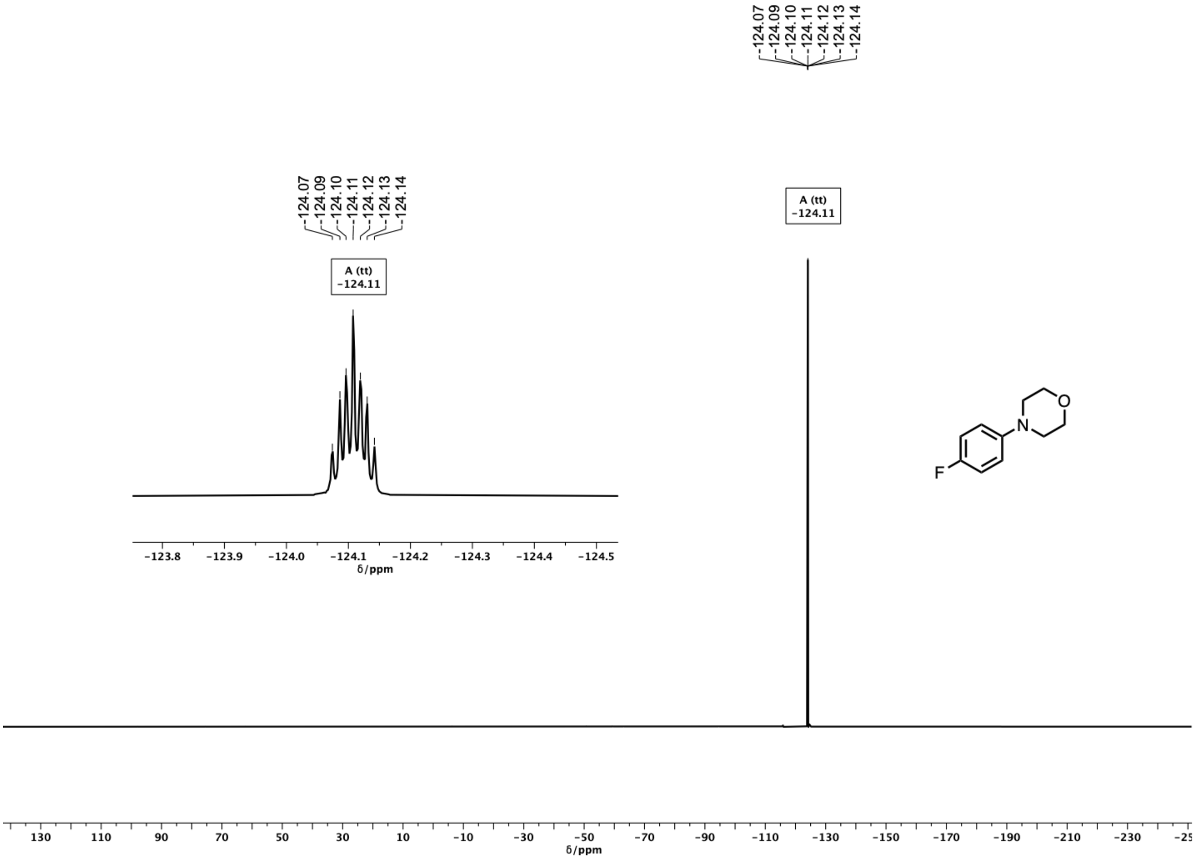

Figure S30: ^{19}F NMR spectrum ($CDCl_3$, 565 MHz, 8 scans) of 4-(4-fluorophenyl) morpholine (**14**).

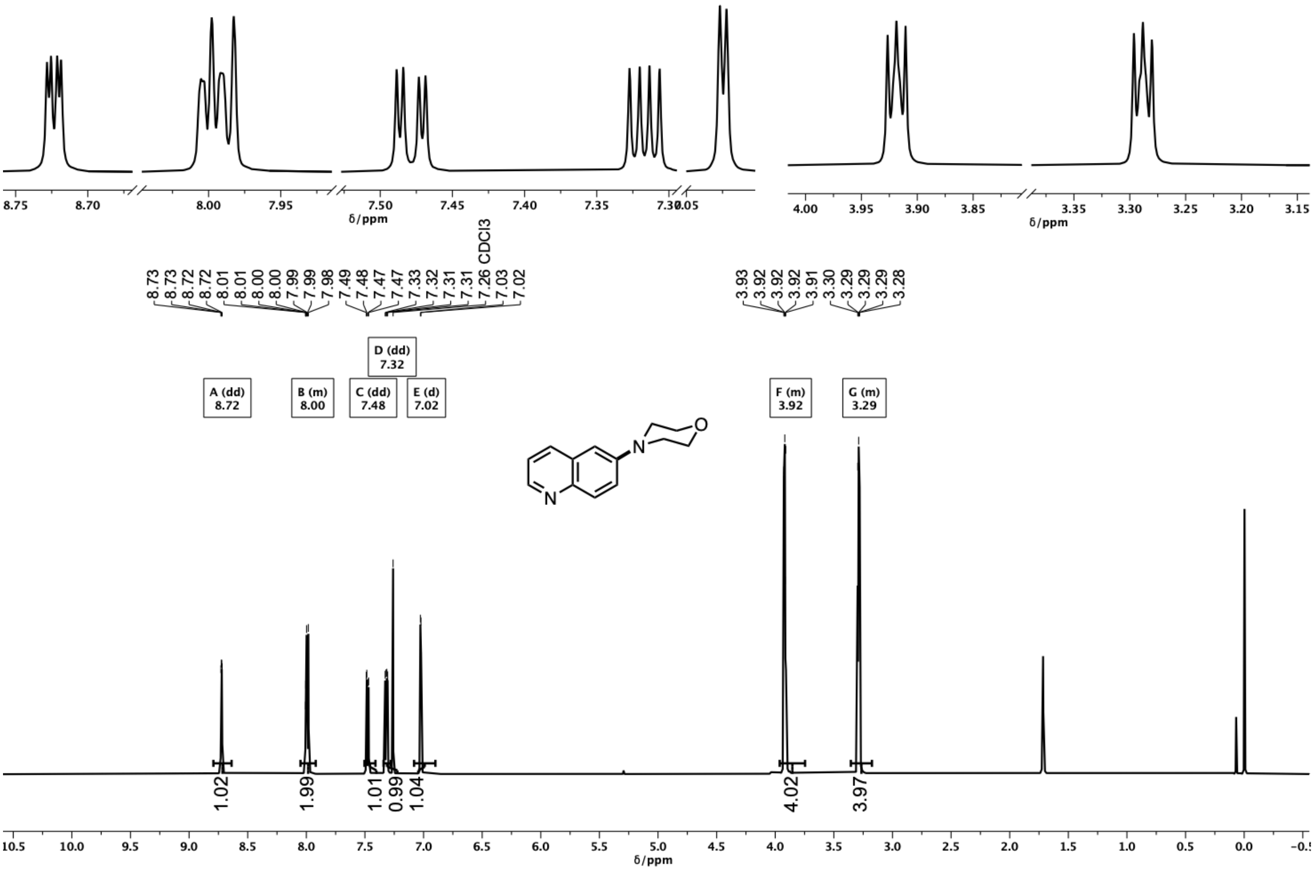

Figure S31: ^{1}H NMR spectrum ($CDCl_3$, 400 MHz, 8 scans) of 4-(quinolin-6-yl) morpholine (**16**).

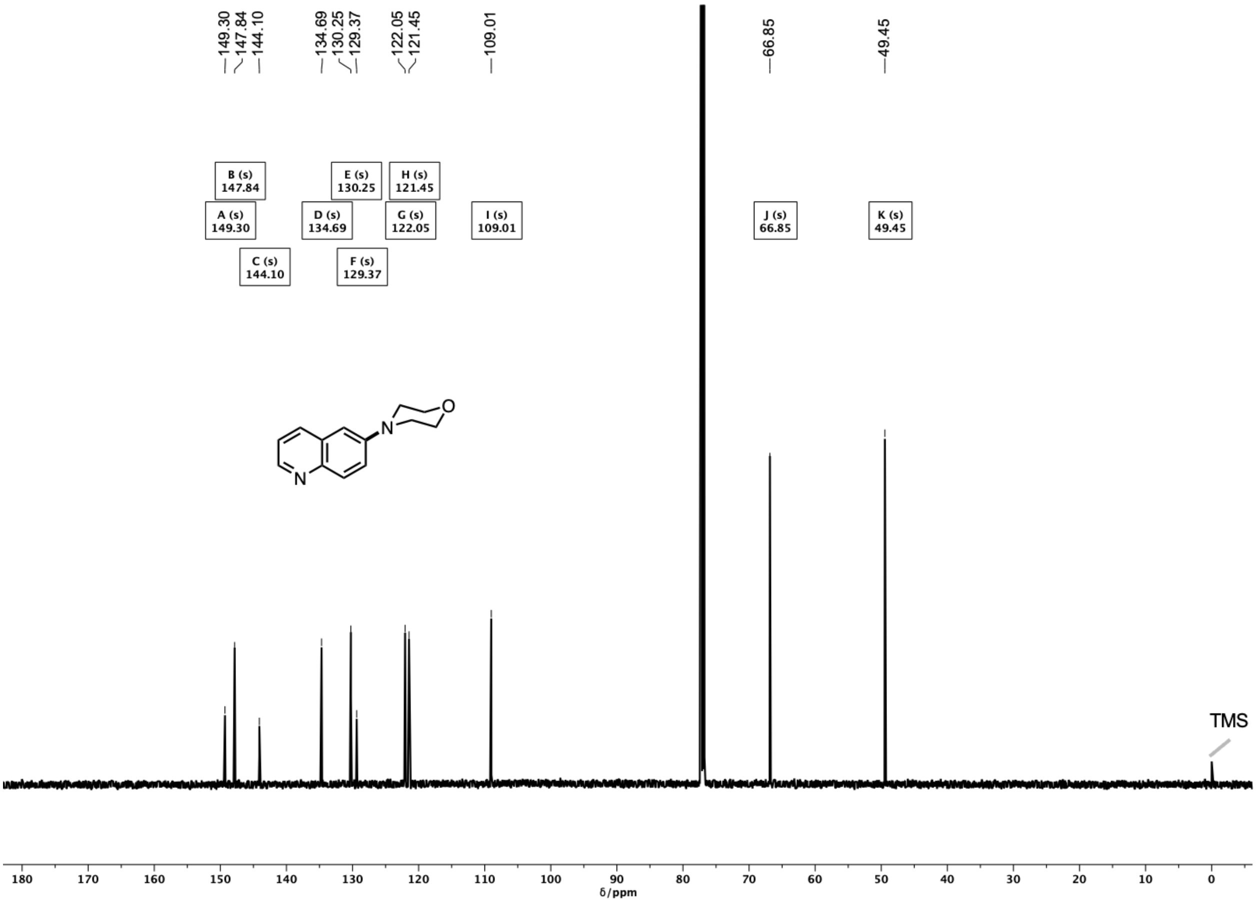

Figure S32: ^{13}C NMR spectrum ($CDCl_3$, 101 MHz, 1024 scans) 4-(quinolin-6-yl) morpholine (**16**).

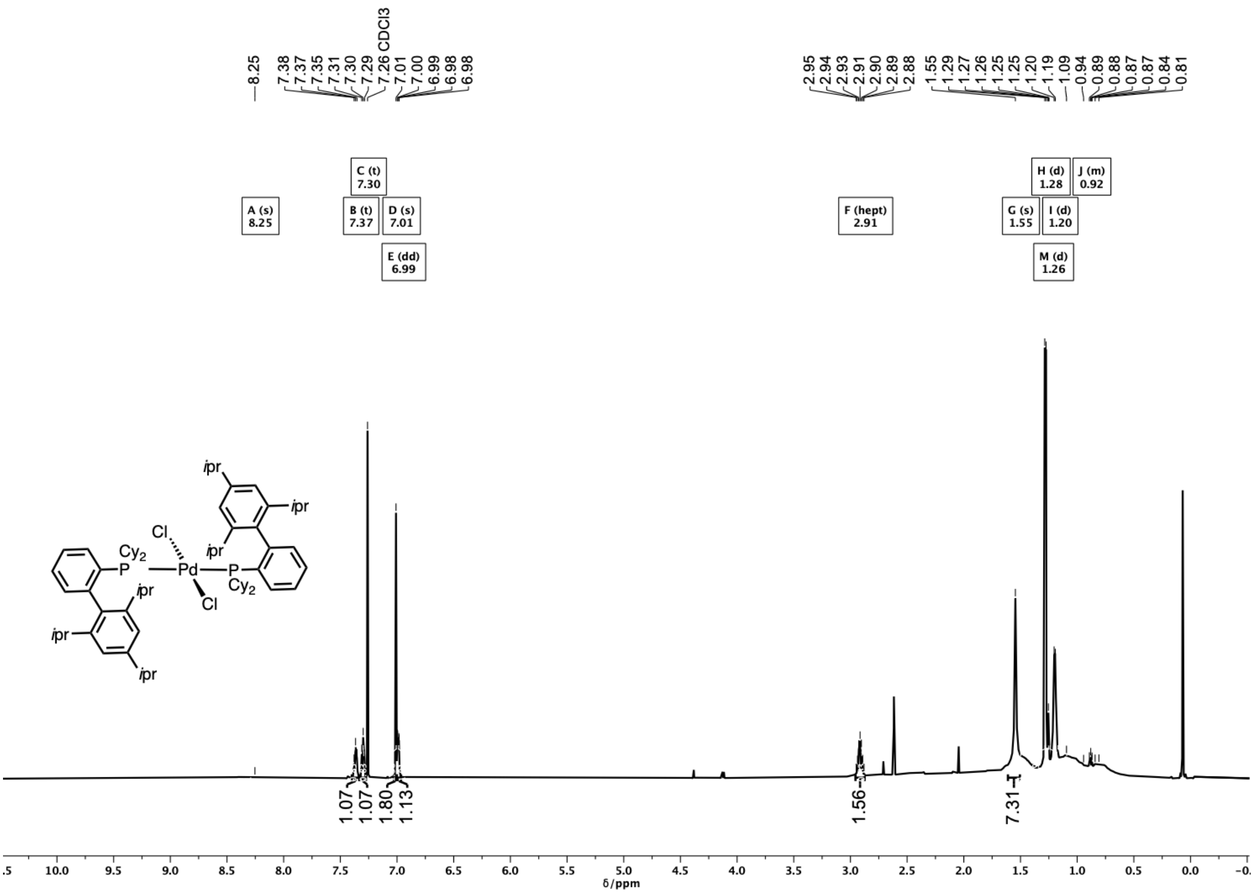

Figure S33: ^{1}H NMR spectrum ($CDCl_3$, 600 MHz, 8 scans) of *bis*-2-dicyclohexylphosphino-2′,4′,6′-triisopropylbiphenyl palladium dichloride (**8**).

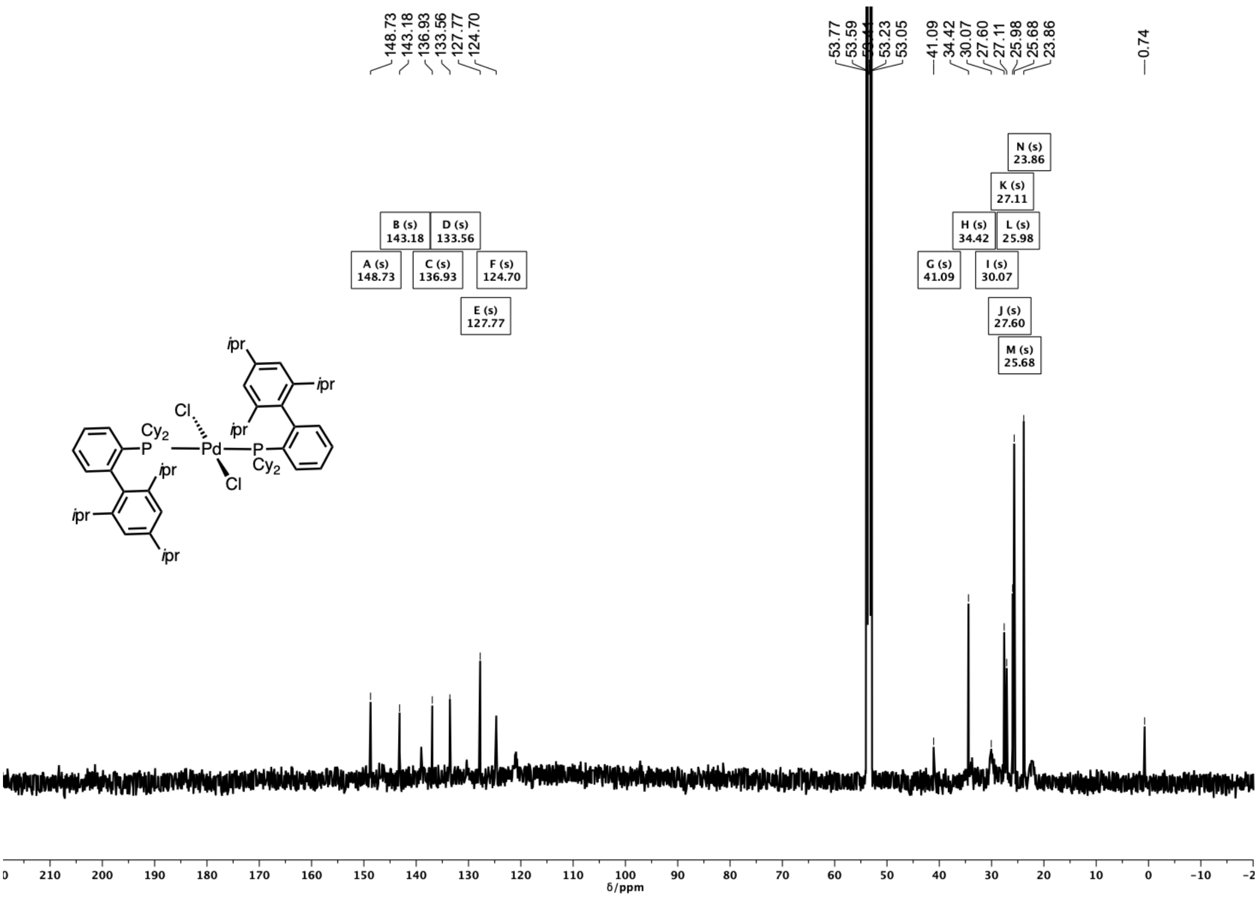

Figure S34: ^{13}C NMR spectrum ($CDCl_3$, 151 MHz, 1024 scans) of *bis*-2-dicyclohexylphosphino-2′,4′,6′-triisopropylbiphenyl palladium dichloride (**8**).

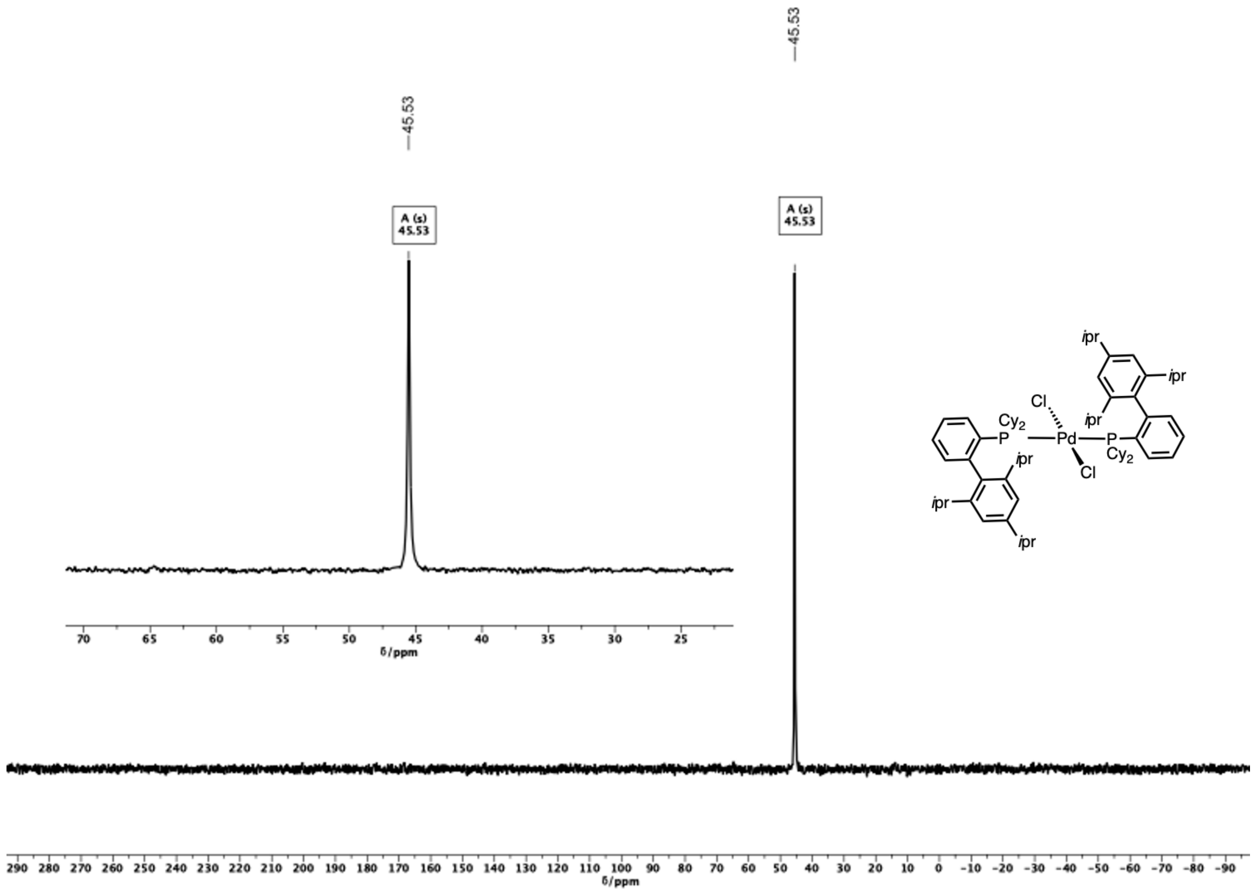

Figure S35: ^{31}P NMR spectrum ($CDCl_3$, 242 MHz, 128 scans) of *bis*-2-dicyclohexylphosphino-2′,4′,6′-triisopropylbiphenyl palladium dichloride (**8**).

5. Abbreviations

BHA	Buchwald–Hartwig amination
Aq.	Aqueous
Sat.	Saturated (solution)
SMCC	Suzuki–Miyaura Cross-Coupling
XPhos	2-Dicyclohexylphosphino-2′,4′,6′-triisopropylbiphenylphosphine
Quant.	Quantitative (yield)

6. References

1. Kahan, R. P., Mateusz Seechurn; Carin. WO 2020/229848 A1, 2020.
2. For commercial information relating to the dyad palladate salts, see: https://matthey.com/products-and-markets/pgms-and-circularity/pgm-chemicals-and-catalysts/catalysts/homogenous/dyad-palladate-kit
3. Borys, A. M. An Illustrated Guide to Schlenk Line Techniques. *Organometallics* **2023**, *42*, 182-196.
4. CrysAlisPro, Oxford Diffraction Ltd. Version 1.171.34.41
5. Dolomanov, O. V.; Bourhis, L. J.; Gildea, R. J.; Howard, J. A. K.; Puschmann, H. OLEX2: a complete structure solution, refinement and analysis program. *J. Appl. Cryst.* **2009**, *42*, 339-341.
6. Sheldrick, G. SHELXT - Integrated space-group and crystal-structure determination. *Acta Crystallogr. A* **2015**, *71*, 3-8.
7. Sinha, N.; Heijnen, D.; Feringa, B. L.; Organ, M. G. Murahashi Cross-Coupling at −78 °C: A One-Pot Procedure for Sequential C−C/C−C, C−C/C−N, and C−C/C−S Cross-Coupling of Bromo-Chloro-Arenes. *Chem. Eur. J.* **2019**, *25*, 9180-9184.
8. Scott, N. W.; Ford, M. J.; Schotes, C.; Parker, R. R.; Whitwood, A. C.; Fairlamb, I. J. S. The ubiquitous cross-coupling catalyst system 'Pd $(OAc)_2$'/2PPh_3 forms a unique dinuclear Pd I complex: an important entry point into catalytically competent cyclic Pd 3 clusters. *Chem. Sci.* **2019**, *10*, 7898-7906.
9. Hemgesberg, M.; Ohlmann, D. M.; Schmitt, Y.; Wolfe, M. R.; Müller, M. K.; Erb, B.; Sun, Y.; Gooßen, L. J.; Gerhards, M.; Thiel, W. R. Simple Access to Sol–Gel Precursors Bearing Fluorescent Aromatic Core Units. *Eur. J. Org. Chem.* **2012**, 2142-2151.
10. Wagschal, S.; Perego, L. A.; Simon, A.; Franco-Espejo, A.; Tocqueville, C.; Albaneze-Walker, J.; Jutand, A.; Grimaud, L. Formation of XPhos-Ligated Palladium(0) Complexes and Reactivity in Oxidative Additions. *Chem. Eur. J.* **2019**, *25*, 6980-6987.
11. Sather, A. C.; Lee, H. G.; De La Rosa, V. Y.; Yang, Y.; Müller, P.; Buchwald, S. L. A Fluorinated Ligand Enables Room-Temperature and Regioselective Pd-Catalyzed Fluorination of Aryl Triflates and Bromides. *J. Am. Chem. Soc.* **2015**, *137*, 13433-13438.
12. Lim, C.-H.; Kudisch, M.; Liu, B.; Miyake, G. M. C–N Cross-Coupling via Photoexcitation of Nickel–Amine Complexes. *J. Am. Chem. Soc.* **2018**, *140*, 7667-7673.
13. Sengmany, S.; Daili, F.; Kribii, I.; Léonel, E. Electrogenerated Nickel Catalyst for C–N Cross-Coupling. *J. Org. Chem.* **2023**, *88*, 675-683.
14. Dong, Z.; Lu, G.; Wang, J.; Liu, P.; Dong, G. Modular ipso/ortho Difunctionalization of Aryl Bromides via Palladium/Norbornene Cooperative Catalysis. *J. Am. Chem. Soc.* **2018**, *140*, 8551-8562.
15. (a) Wakioka, M.; Hatakeyama, K.; Sakai, S.; Seki, T.; Tada, K.-i.; Mizuhata, Y.; Nakazato, T.; Koguchi, S.; Shibuya, Y.; Maruyama, Y.; Ayabe, M. Mixed-Ligand Approach to Palladium-Catalyzed Direct Arylation of Heteroarenes with Aryl Chlorides: Controlling Reactivity of Catalytic Intermediates via Dynamic Ligand Exchange. *Organometallics* **2023**, *42*, 3454-3465. (b) Sirindil, F; Weibel, J.-M.; Pale, P.; Blanc, A. Total Synthesis of Rhazinilam through Gold-Catalyzed Cycloisomerization–Sulfonyl Migration and Palladium-Catalyzed Suzuki–Miyaura Coupling of Pyrrolyl Sulfonates. *Org. Lett.* **2019**, *21*, 5542-5546.
16. Singh, R. B.; Mitra, S.; Kundu, P. Thermal and stereochemical studies of some cyclic ligand complexes of palladium(II) in the solid state. *Thermochim. Acta* **1996**, *285*, 191-197.
17. Santamaría, N.; Velasco, C.; Marín, M.; Maya, C.; Nicasio, M. C. $LPdCl_2$(amine) complexes supported by terphenyl phosphanes: applications in aryl amination reactions. *Dalton Trans.* **2022**, *51*, 15734-15740.

18. Bray, Joshua (2016) Rationalising Pd-Precatalyst Design for Efficient Arylcyanation Reactions. PhD thesis, University of York.
19. Khan, S. R. A.; Guzman-Jimenez, I.; Whitmire, K. H.; Khokhar, A. R. Synthesis and characterization of platinum(II) complexes with 3-methylpiperidine: crystal and molecular structure of [Pt(3-methylpiperidine)$_2$(malonato)]·H_2O. *Polyhedron* **2000**, *19*, 983-989.
